# NOTCH1 acts as a tumor suppressor that induces early differentiation in head and neck cancer

**DOI:** 10.1172/jci.insight.202414

**Published:** 2026-04-16

**Authors:** Chenfei Huang, Shhyam Moorthy, Qiuli Li, Kazi M. Ahmed, Kalil Saab, Defeng Deng, Jiping Wang, Xiayu Rao, Jiexin Zhang, Yuanxin Xi, Jing Wang, Zhiyi Liu, Noriaki Tanaka, David A. Wheeler, Eve Shinbrot, Rami Saade, Curtis R. Pickering, Tong-Xin Xie, Adel K. El-Naggar, Abdullah A. Osman, Kunal Rai, Patrick A. Zweidler-McKay, John V. Heymach, Lauren A. Byers, Faye M. Johnson, Vlad C. Sandulache, Jeffrey N. Myers, Pedram Yadollahi, Mitchell J. Frederick

**Affiliations:** 1 Bobby R. Alford Department of Otolaryngology-Head and Neck Surgery, Baylor College of Medicine, Houston, Texas, USA.; 2 Celularity Inc., Florham Park, New Jersey, USA.; 3 Department of Head and Neck Surgery, University of Texas MD Anderson Cancer Center, Houston, Texas, USA.; 4 Merck, Rahway, New Jersey, USA.; 5 Department of Head and Neck, Sun Yat-sen University Cancer Center, Guangzhou, China.; 6 Department of Integrative Medicine, and; 7 Department of Bioinformatics and Computational Biology, University of Texas MD Anderson Cancer Center, Houston, Texas, USA.; 8 LC Sciences, Houston, Texas, USA.; 9 Department of Oral and Maxillofacial Surgery, Osaka University School of Dentistry, Suita City, Osaka, Japan.; 10 Human Genome Sequencing Center, Baylor College of Medicine, Houston, Texas, USA.; 11 Department of Computational Biology, St. Jude Children’s Research Hospital, Memphis, Tennessee, USA.; 12 Department of Otolaryngology, Lebanese American University, Beirut, Lebanon.; 13 Department of Surgery-Otolaryngology, Yale School of Medicine, New Haven, Connecticut, USA.; 14 Department of Pathology,; 15 Department of Genomic Medicine and MDACC Epigenomics Therapy Initiative,; 16 Department of Pediatric Leukemia and Lymphoma, and; 17 Department of Thoracic/Head & Neck Medical Oncology, University of Texas MD Anderson Cancer Center, Houston, Texas, USA.; 18 The University of Texas Graduate School of Biomedical Sciences, Houston, Texas, USA.; 19 ENT Section, Operative Care Line, and; 20 Center for Translational Research on Inflammatory Diseases, Michael E. DeBakey Veterans Affairs Medical Center, Houston, Texas, USA.

**Keywords:** Cell biology, Oncology, Head and neck cancer, Stem Cells, Tumor suppressors

## Abstract

Inactivating *NOTCH1* mutations in head and neck squamous cell carcinoma (HNSCC) were described over a decade ago, suggesting a tumor suppressor function — unlike its oncogenic role in other tumors. Today, much debate persists regarding a putative oncogenic role in HNSCC as well, with reports that NOTCH1 signaling drives tumor growth and a cancer stem cell (CSC) phenotype. In this work, comprehensive experiments unequivocally demonstrate that *NOTCH1* is a tumor suppressor in HNSCC regardless of mutation or activation status and that it reduces CSC frequency. We developed a signature of NOTCH1 activation showing the pathway is associated with very early differentiation, an altered tumor microenvironment, and better prognosis. Clarifying whether *NOTCH1* occasionally functions as an oncogenic driver in HNSCC is crucial to prognosis and personalized therapy. The results presented unify the field, reconcile conflicting data, and provide critical insights into the biological and clinical significance of *NOTCH1*, with broader implications in other squamous carcinomas with *NOTCH1* mutations.

## Abstract 2

Our study shows the NOTCH1 gene acts as a tumor suppressor in head and neck cancer, limiting cancer stem cells and linking pathway activity to better prognosis.

## Introduction

The genomic landscape of head and neck squamous cell carcinoma (HNSCC) is predominated by tumor suppressors (1–4), posing challenges for the development of molecularly targeted therapies. We identified frequent inactivating *NOTCH1* mutations in HNSCC (1, 3) and aggressive cutaneous SCC (cSCC) (5), indicating a potential tumor suppressive role. Subsequent investigations have confirmed similar mutation patterns in HNSCC (4, 6, 7), cSCC (8), and SCCs of the lung (LUSC) (9) and esophagus (ESCC) (10) through The Cancer Genome Atlas (TCGA) and independent studies, solidifying *NOTCH1* as one of the most commonly mutated genes across various SCCs. The presence of mutations in extracellular ligand binding domains and truncating mutations throughout the *NOTCH1* gene in SCCs aligns with its proposed tumor suppressor function (11), supported by earlier mouse studies demonstrating increased skin tumors upon conditional *NOTCH1* knockout (KO) (12). Conversely, *NOTCH1* is an oncogenic driver in T cell acute lymphoblastic leukemia, where activating missense mutations cluster in the heterodimerization (HD) domain and truncating mutations occur in the C-terminal PEST sequence, leading to increased *NOTCH1* activation (11, 13). This oncogenic role has also been reported in adenoid cystic carcinomas originating from the salivary gland (14), highlighting NOTCH1’s context-specific dual role in cancer biology.

Although NOTCH1 plays opposing roles in cancers from different tissues, multiple studies suggest a dual function within HNSCC of the same histology, including an oncogenic role (7, 15–19). Activating mutations in the HD and Abruptex regions have been reported in Asian HNSCC cohorts (20, 21), although subsequent cloning of an Abruptex mutation later revealed that it was inactivating (22). Additionally, NOTCH1 RNA and protein overexpression has been correlated with poor prognosis (15, 17, 18, 23), and pharmacological inhibition or gene knockdown has linked NOTCH1 activation to proliferation (16, 19, 23), tumor growth (18, 24), spheroid formation (19, 24–26), and resistance to chemotherapy (25). In contrast, we demonstrated that ectopic expression of activated intracellular NOTCH1 (ICN1) expression inhibits proliferation and tumor growth in *NOTCH1*-mutant HNSCC lines (2), supported by a report that nuclear cleaved NOTCH1 by IHC correlated with better patient survival (27).

Clarifying whether the NOTCH1 pathway occasionally functions as an oncogenic driver in HNSCC is crucial not only for academic discourse but also for the prognosis and personalized therapy of SCC patients with either wild-type (WT) or mutated *NOTCH1*. While NOTCH1 inhibition has been proposed for HNSCC tumors where the pathway is deemed oncogenic, we reported that HNSCC cell lines harboring inactivating *NOTCH1* mutations are highly sensitive to PI3K inhibitors (28–30), underscoring the complex interplay of signaling pathways in SCC. Here, we present a systematic and comprehensive analysis demonstrating that *NOTCH1* functions as a tumor suppressor in HNSCC regardless of mutation or activation status, despite its potential to induce pseudo–stem cell–like properties in vitro. We further demonstrate that restoration or activation of NOTCH1 signaling induces a program of very early differentiation — also manifested in human HNSCC primary tumors — that reshapes the tumor microenvironment and may influence which tumor dependencies can be clinically targeted successfully. Our findings challenge prevailing models that consider the dedifferentiated state of SCCs to be irreversible due to genetic mutations, offering deeper insights into the mechanisms of tumor plasticity.

## Results

### NOTCH1 restoration inhibits growth in 2-dimensional cultures and alters morphology of HNSCC cell lines harboring inactivating NOTCH1 mutations.

To understand the consequences of NOTCH1 signaling in HNSCC lines harboring *NOTCH1* inactivating mutations (Supplemental Table 1; supplemental material available online with this article; https://doi.org/10.1172/jci.insight.202414DS1), we reexpressed WT full-length *NOTCH1* receptor (NFL1) using a bicistronic retroviral vector harboring an IRES-EGFP tag. This allowed purification and testing of NFL1-expressing cells while avoiding artifacts of long-term selection. Following NFL1 overexpression, cells were continuously cultured (1–10 days) on plates precoated with either recombinant NOTCH1 ligand Jagged1 fused to an Fc receptor (JAG1) or immobilized control Fc protein. We detected cleaved/activated intracellular NOTCH1 (cl-NOTCH1) protein only in *NOTCH1* mutants infected with NFL1 and not empty vector control virus (MigR1), which increased substantially after just 16 hours of growth on JAG1 compared with Fc control protein (Supplemental Figure 1A) and confirmed that endogenous *NOTCH1* mutations were indeed inactivating. NFL1 overexpression alone, without external JAG1, modestly decreased colony formation in both UMSCC47 and UMSCC22A (Supplemental Figure 1, B and C) over a 10-day period. However, when grown in the presence of immobilized JAG1 ligand, NFL1 expression led to a significant reduction in colony formation in 4 different *NOTCH1*-mutant cell lines when compared with growth on control Fc protein (Supplemental Figure 1, B and C).

Reduced colony growth in *NOTCH1*-mutant UMSCC22A expressing WT NFL1 that were exposed to JAG1 was accompanied by the onset of profound morphological changes after 3 to 5 days, which included a vast reduction in cell size and compact growth as loosely attached spheroids (Supplemental Figure 1D). A fraction of *NOTCH1*-mutant HN4 and HN31 cells expressing NFL1 became spindle-shaped after 3 to 5 days of growth on JAG1 and positive for the senescent marker β-galactosidase (β-Gal) (Supplemental Figure 2, A and B). Likewise, the loosely formed spheroids produced by *NOTCH1*-mutant UMSCC22A cells that express NFL1 and were grown on JAG1 showed considerable β-Gal staining (Supplemental Figure 2A). Collectively, reactivating NOTCH1 signaling in mutant tumors profoundly inhibited cell growth in 2-dimensional cultures and led to altered morphology and senescence.

### HNSCC cell lines harboring WT NOTCH1 show similar patterns of growth inhibition and altered morphology following NOTCH1 activation.

**Figure 1:**
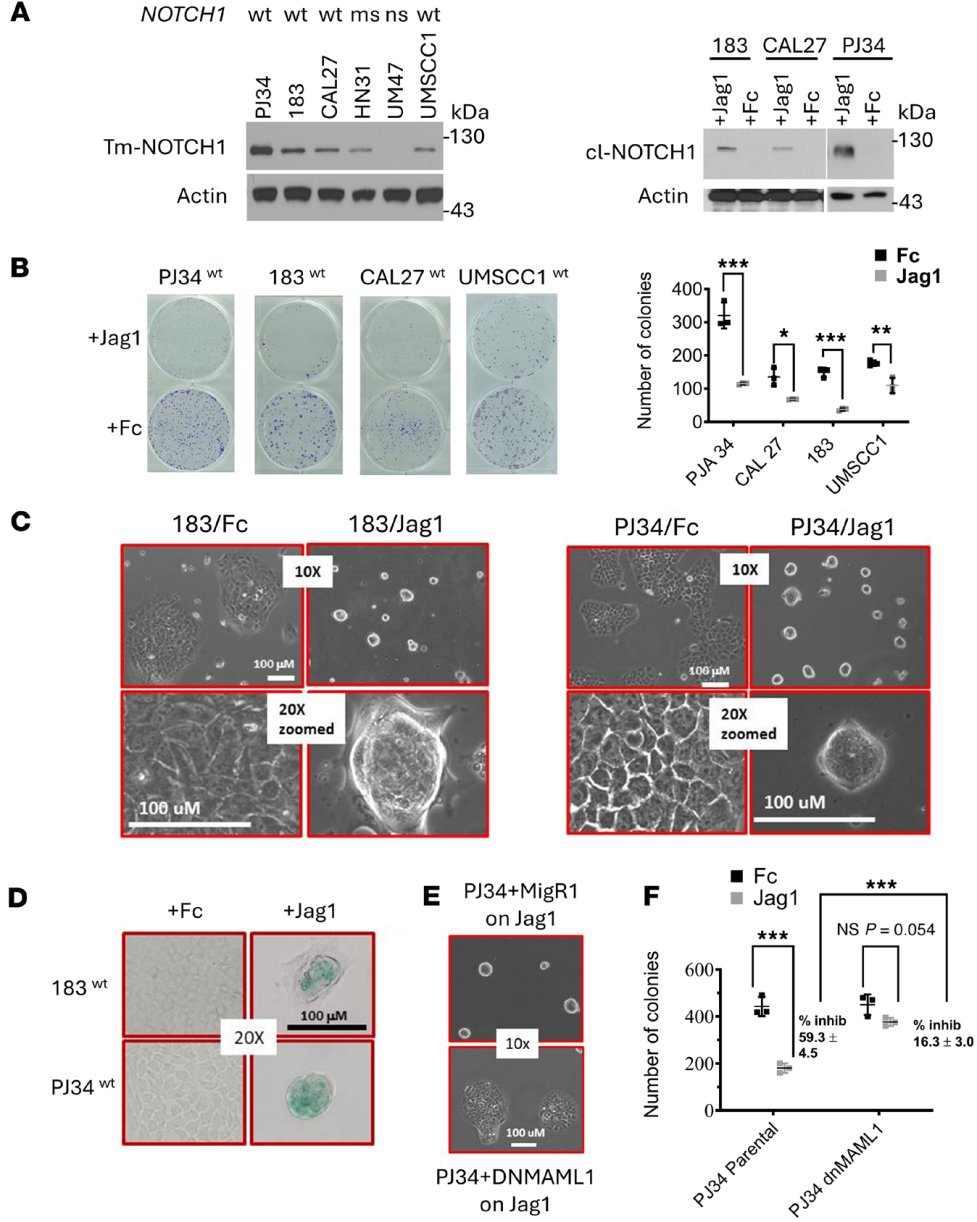
NOTCH signaling alters cell morphology and inhibits growth in 2-dimensional cultures. (**A**) Expression of full-length transmembrane NOTCH1 (Tm-NOTCH1) in cells with WT *NOTCH1* (PJ34, 183, CAL27, and UMSCC1) and cleaved activated NOTCH1 (cl-NOTCH1) protein after 16 hours of growth on immobilized NOTCH1 ligand (JAG1) or Fc control protein (Fc). HN31 and UM47 harbor homozygous missense (ms) and nonsense (ns) *NOTCH1* mutations, respectively. (**B**) Extended growth (e.g., 8–10 days) on JAG1 ligand significantly reduced colony formation compared with control Fc protein in 4 *NOTCH1*-WT cell lines. (**C**) Growth on JAG1, but not control Fc, induces morphological transformation of cell lines with WT *NOTCH1* (183 and PJ34) observed by 5 days, characterized by reduced cell size and formation of loosely attached tumor spheroids. (**D**) Tumor spheroids induced by growth on JAG1 express the senescence marker β-Gal. (**E**) Ectopic expression of dominant negative MAML1 (dnMAML1), which inhibits *NOTCH1*-mediated transcriptional regulation, prevents JAG1-induced tumor spheroid formation, and reverses inhibition of colon formation in PJ34 cells. (**F**) Quantitation of colonies from parental PJ34 or PJ34 expressing dnMAML1 cultured on either control Fc or JAG1 protein. Scale bars: 100 μm (**C**–**E**). Differences between Fc and JAG1 treatment (simple contrasts) or comparison of JAG1-mediated inhibition in parental or cells expressing dnMAML1 (interaction contrasts) were determined with a cell means model. *P < 0.05; **P < 0.01; ***P < 0.001.

Next, we examined the morphological and proliferation phenotypes associated with NOTCH1 pathway activation in 4 randomly selected HNSCC cell lines expressing endogenous WT NFL1 receptors (Supplemental Table 1). Expression of full-length transmembrane NOTCH1 (Tm-NOTCH1) receptor proteins at various levels was confirmed by Western blotting (Figure 1A). Levels of cl-NOTCH1 protein were examined in 3 of the cell lines grown on control Fc protein and found to be barely detectable but increased following a brief 16-hour exposure to JAG1 (Figure 1A). Extended cultivation of *NOTCH1*-WT tumors on immobilized JAG1 inhibited colony formation (Figure 1B) in 4 cell lines tested (PJ34, 183, CAL27, and UMSCC1), particularly in 3 of 4 cell lines where JAG1-induced NOTCH1 activation was confirmed (e.g., Figure 1A), consistent with growth inhibition observed in *NOTCH1* mutants. In the fourth *NOTCH1*-WT cell line, UMSCC1, JAG1 induced a mild but significant reduction in colony formation (Figure 1B). Remarkably, growth of 183 and PJ34 cell lines on JAG1 (but not control Fc protein) led to the very same morphological transformation found earlier in UMSCC22A, characterized by substantial cell shrinkage and the formation of loosely attached compacted tumor spheroids (Figure 1C). These spheroids also displayed positive staining for β-Gal (Figure 1D).

The growth inhibition and associated morphological changes induced by JAG1 exposure in PJ34 were effectively reversed by expressing a dominant negative form of Mastermind-like 1 (Figure 1, E and F), known to inhibit NOTCH family signaling. Intrigued, we aimed to dissect the individual phenotypic contributions of NOTCH1 and NOTCH2 signaling in PJ34, since both receptors can be triggered by their shared ligand JAG1. Through CRISPR-mediated KO experiments targeting *NOTCH1*, *NOTCH2*, or both genes (Supplemental Figure 3A), we observed that KO of either NOTCH gene only partially rescued PJ34 from the growth inhibition induced by JAG1 (Supplemental Figure 3B). However, double KO of both genes (N1N2KO) completely prevented JAG1-mediated growth inhibition (Supplemental Figure 3B) and prevented morphological formation of tumor spheroids (Supplemental Figure 3E). Reexpression of NFL1 alone in N1N2KO cells was sufficient to restore JAG1-induced NOTCH1 activation (Supplemental Figure 3C), growth inhibition (Supplemental Figure 3D), and morphological changes (Supplemental Figure 3E).

### NOTCH1 activation does not drive proliferation in HNSCC tumors with high endogenous NOTCH1 signaling.

**Figure 2:**
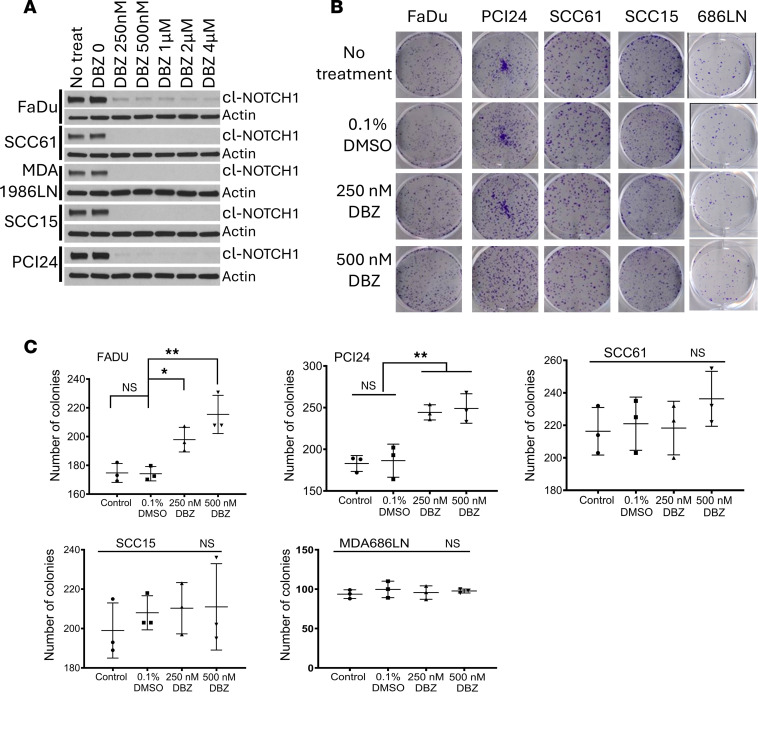
NOTCH1 is not a driver of cell growth in multiple HNSCC cell lines with high endogenous NOTCH1 activation. (**A**) Western blot validation of high basal levels of cl-NOTCH1 protein in 5 untreated *NOTCH1*-WT HNSCC cell lines (i.e., no treatment) with different genomic backgrounds and persistent inhibition of NOTCH1 signaling after 72 hours of treatment with various doses of the NOTCH1 inhibitor DBZ. (**B**) Staining of colony formation in the presence or absence of continuous treatment with γ-secretase inhibitor DBZ (replaced every 48 hours) for the duration of culture. (**C**) Quantitation of colony formation shows no decrease in growth after continued treatment with DBZ. For each cell line, comparisons between treatment groups were analyzed by 1-way ANOVA and individual comparisons were made using a post hoc Tukey’s test. *P < 0.05; **P < 0.01.

Because previous studies linked NOTCH1 signaling to proliferation and cancer stem cell–like (CSC-like) behavior in HNSCC cell lines, we investigated the function of NOTCH1 signaling in tumors with high endogenous levels of activated cl-NOTCH1 initially identified by reverse-phase protein arrays (RPPAs). Levels of cl-NOTCH1 along with 155 other proteins/phosphoproteins, including total NOTCH1, were measured across 53 different HNSCC cell lines with known *NOTCH1* and *NOTCH2* mutational status (Supplemental Table 1). As predicted, RPPA levels of cl-NOTCH1 were significantly lower in cell lines harboring *NOTCH1* mutations (*P* = 0.03, Supplemental Figure 4A). Western blotting (Supplemental Figure 4, B and C) confirmed relatively high levels of baseline cl-NOTCH1 protein in 6 cell lines (FaDu, SCC61, SCC15, PCI24, MDA1986LN, and MDA686LN) identified by RPPA, compared with 2 of the *NOTCH1*-WT cell lines, PJ34 and CAL27, utilized earlier and found to undergo JAG1-mediated growth inhibition (e.g., Figure 1B and Supplemental Figure 4B). To evaluate the necessity of NOTCH1 signaling in these cells with high cl-NOTCH1 levels, we inhibited the formation of cl-NOTCH1 using 250 or 500 nM γ-secretase inhibitor dibenzazepine (DBZ) in all 6 cell lines (Figure 2A and Supplemental Figure 4D). The inhibition of NOTCH1 activation persisted 48 to 72 hours after DBZ treatment and continuous exposure to DBZ (refreshed every 48 hours) failed to inhibit clonogenic growth in *NOTCH1*-WT cells FADU, PCI24, SCC61, SCC15, or MDA686LN (Figure 2, B and C). On the contrary, there was even a slight increase in colony formation for 2 of the cell lines with DBZ. Although MDA1986LN failed to form colonies, its growth was unaffected by DBZ in standard proliferation assays (not shown).

### Proteins correlating with NOTCH1 activation in HNSCC cell lines.

The top protein correlating with cl-NOTCH1 levels among analytes analyzed by RRPA was total NOTCH1 (*r* = 0.679, Adj*P* = 3.50 × 10^–6^, Supplemental Table 2), followed by FBXW7 (*r* = 0.612) and EZH2 (*r* = 0.608). FBXW7 is known to degrade active cl-NOTCH1 in the nucleus and likely represents negative feedback, while EZH2 is a histone methyltransferase that represses gene expression. NRF2, which is a stress-induced transcriptional activator responsive to reactive oxygen species (ROS), was also positively correlated (*r* = 0.449), as was KEAP1 (0.331) that negatively regulates NRF2, and NF-κB-p65 (*r* = 0.382), which also responds to ROS. Among significantly anticorrelated proteins were the receptor tyrosine kinase AXL (*r* = –0.457) that regulates survival and proliferation and fibronectin (*r* = –0.385), an extracellular matrix protein that can mediate binding of fibroblasts.

### Persistent NOTCH1 signaling downregulates proto-oncogenes, growth factors, and integrins while increasing expression of early differentiation markers.

**Figure 3:**
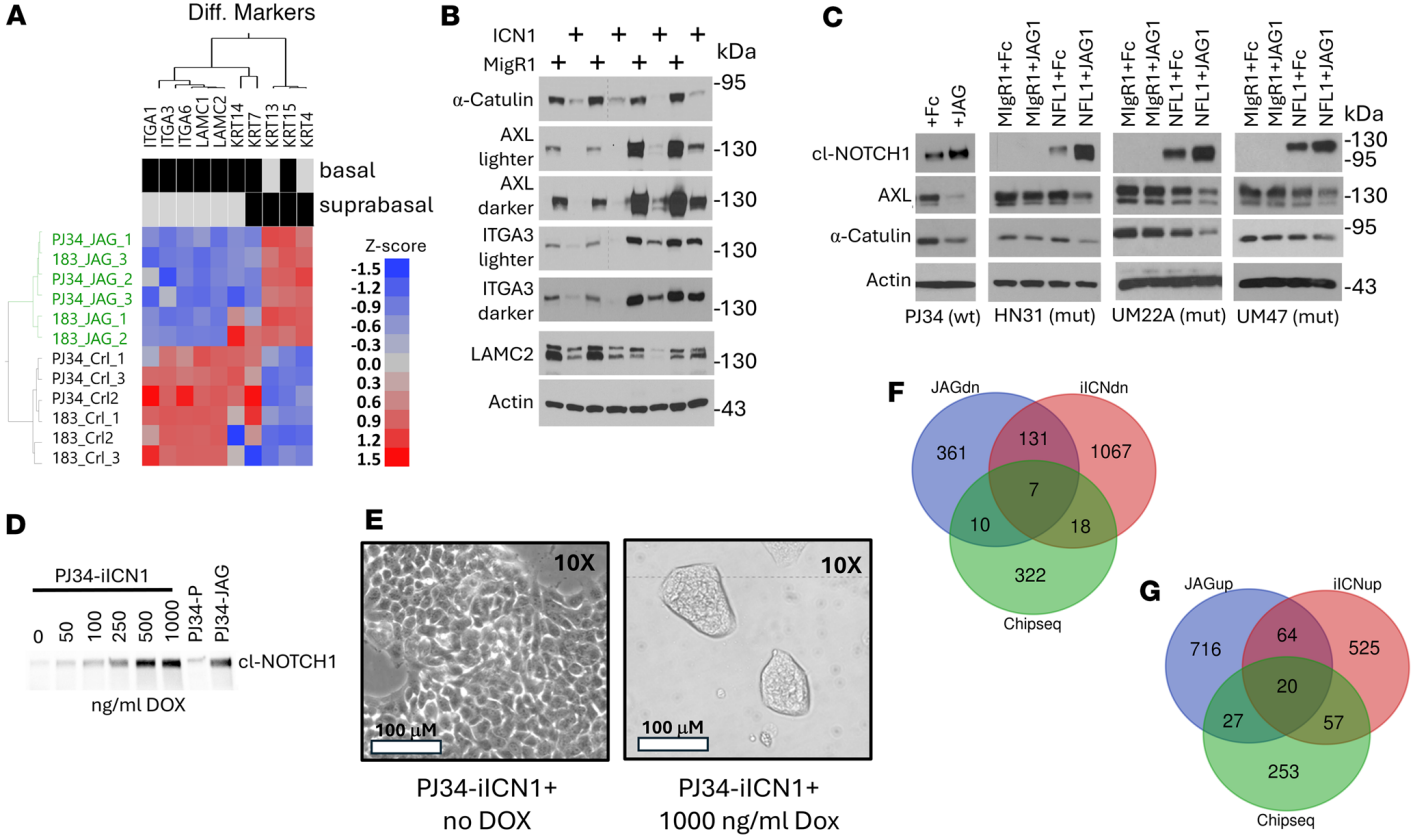
The activation of NOTCH1 signaling through multiple experimental approaches consistently downregulates oncogenic drivers and modulates adhesion or other markers of early differentiation. (**A**) Growth for 5 days on JAG1 significantly inhibited RNA expression of the basal cell marker genes *ITGA1*, *ITGA3*, *ITGA6*, *LAMC1*, and *LAMC2* in 2 *NOTCH1*-WT cell lines, PJ34 and 183, while stimulating expression of the suprabasal maker genes *KRT4* and *KRT13*. (**B**) Ectopic expression of cDNA encoding cl-NOTCH1 decreased expression of ITGA3, LAMC2, AXL, and α-catulin at 3 days and 5 days after infection in a *NOTCH1*-WT cell (PJ34) and in a *NOTCH1*-mutant cell (HN31). (**C**) AXL and α-catulin protein levels decline in *NOTCH1*-WT (PJ34) tumor cells grown on JAG1 (3 days) and in 3 different *NOTCH1*-mutant cell lines (HN31, UMSCC22A, UMSCC47) when expression of WT full-length NOTCH1 receptors (NFL1) is restored and then grown on JAG1 ligand for 3 days. Cells were also infected with empty vector (MigR1) and/or grown on control Fc protein. (**D**) PJ34 with CRISPR-mediated *NOTCH1* KO were further engineered to express a DOX-inducible cl-NOTCH cDNA (PJ34-iICN1) whose expression was titrated after 72 hours of treatment with different doses of DOX, to achieve protein levels equivalent to parental PJ34 stimulated with JAG1 for 16 hours. (**E**) ICN1 induction with 1000 ng/mL DOX caused massive cell shrinkage and formation of loosely attached tumor spheroids. Scale bars: 100 μm. (**F**) Venn diagram illustrates overlap of genes in PJ34 significantly upregulated (FDR < 0.1, |fold change| ≥ 1.25) by growth on JAG1 or after 24 hours of iICN1 induction, and genes specifically bound in the promoter or gene body regions by iICN1 after ChIP-seq experiments. (**G**) Venn diagram illustrates overlap of genes in PJ34 significantly downregulated (FDR < 0.1, |fold change| ≥ 1.25) by growth on JAG1 or after 24 hours of iICN1 induction, and genes specifically bound in the promoter or gene body regions by iICN1 after ChIP-seq experiments.

To elucidate the molecular mechanisms and understand phenotypes associated with prolonged activated NOTCH1 signaling, we examined changes in gene expression in *NOTCH1*-WT HNSCC cell lines (PJ34 and 183) induced by growth on JAG1 ligand for 5 days. A 2-way ANOVA identified genes downregulated (Supplemental Table 3) and upregulated (Supplemental Table 4) due to growth on JAG1 as a main effect (e.g., JAG treatment) and for the individual cell lines PJ34 and 183 grown on JAG1 in post hoc analyses. We focused on the top significant genes that showed at least a 1.4-fold change in both cell lines and found 50 genes upregulated and 70 downregulated because of JAG1 exposure. Gene Ontology (GO) enrichment analysis of these 120 altered genes identified pathways related to proliferation, differentiation, cell adhesion, cytokine production, response to oxygen-containing compounds, and cytokine production (Supplemental Tables 5 and 6, and Supplemental Figure 5). Among the top genes downregulated by NOTCH activation were 3 prosurvival/proliferation genes, *CTNNAL1* (α-catulin), *AXL*, and *EREG* (epiregulin). Notably, *CTNNAL1* exhibited the most significant reduction in magnitude among all genes (e.g., 24-fold in 183) following NOTCH activation. *AXL* ranked among the top 5 genes with the greatest reduction in both cell lines and was consistent with the earlier finding of an inverse correlation between AXL protein and cl-NOTCH1 by RRPA (Supplemental Table 2). Multiple genes regulating cellular adhesion, including *ITGA3* (integrin α3), *ITGA5* (integrin α5), *LAMC2* (laminin γ2), and *LAMC1*, were downregulated in both cell lines after NOTCH activation (Supplemental Table 3). Keratins 4 and 13 emerged as the top 2 upregulated genes in 183 cells (7.9- and 3.6-fold, respectively) and among the top 15 upregulated genes in PJ34 (2.0- and 1.8-fold, respectively) following NOTCH activation (Supplemental Table 4). Additionally, putative tumor suppressors such as *EPHA4*, *TP53INP1*, *PDCD4*, *DSP*, and *TXNIP* were among top genes upregulated by NOTCH activation. The specific pattern of integrins downregulated and keratins upregulated (Figure 3A) mirrors what happens to normal oral mucosa during very early differentiation as basal stem cells divide and migrate upwards to the suprabasal layer of squamous epithelium. Collectively, the genes commonly regulated in both PJ34 and 183 cells after growth on ligand suggest a loss of proliferation and loss of substrate adhesion consistent with very early squamous cell differentiation.

### Validation of genes and proteins regulated by NOTCH1 activation.

Given strong links between AXL and α-catulin and HNSCC aggressiveness, we validated their decreased protein expression following NOTCH1 activation in both WT and mutant tumors using multiple approaches. First, we exogenously expressed activated cl-NOTCH1 using a retroviral construct encoding ICN1 widely used by others for functional studies (31); construct integrity was confirmed as described in Supplemental Methods (Supplemental Figure 6A). Infection with ICN1 but not empty vector MigR1 induced morphological changes in UMSCC22A and *NOTCH1*-WT 183 and PJ34 cells identical to that observed earlier for growth of these cell lines on JAG1 (Supplemental Figure 6, B–D). ICN1 expression caused substantial reduction in AXL and α-catulin proteins in *NOTCH1*-WT PJ34 and 183 (Figure 3B and Supplemental Figure 6E) and *NOTCH1*-mutant HN31 and UMSCC22A (Figure 3B and Supplemental Figure 6A). Likewise, growth of PJ34, or *NOTCH1*-mutant cells with restored NFL1 (HN31, UMSCC22A, and UM47) on JAG1 ligand also suppressed AXL and α-catulin protein levels (Figure 3C). Decreased LAMC2 and ITGA3 protein was also confirmed following ICN1 expression in PJ34 and HN31. Consistent with negative regulation by NOTCH1, increased AXL and α-catulin protein levels were found after pharmacological inhibition of NOTCH1 signaling with DBZ in the *NOTCH1*-WT cell lines with normally high NOTCH1 activation (Supplemental Figure 7).

### NOTCH1 regulates many genes indirectly.

To better understand the mechanisms and timing of NOTCH1 activation, we constructed a doxycycline-inducible (DOX-inducible) ICN1 (iICN1) retroviral construct encoding intracellular NOTCH1 from the known cleavage site, enabling precise control over activation levels and detection by cl-NOTCH1–specific antibodies. We combined this tool with NOTCH1 ChIP-seq experiments to identify genes directly regulated. *NOTCH1/NOTCH2* double-KO PJ34 cells (e.g., Supplemental Figure 3) were engineered to express the Tet3 regulator (PJ34Tet3 cells) along with iICN1 (PJ34-iICN1) so that NOTCH1 was activated in the presence of DOX. Dose-response experiments determined that 500–1000 ng/mL DOX induced levels of cl-NOTCH1 protein equivalent to those found after incubating parental PJ34 (PJ34-P) on JAG1 (Figure 3D). Within 1 week after iICN1 induction with DOX, PJ34-iICN underwent the same morphological transformation observed earlier when parental PJ34 were grown on JAG1, characterized by massive cell shrinkage and formation of loosely attached tumor spheroids (Figure 3E). RNA-seq performed from samples isolated 20 hours after peak iICN1 expression identified 1223 genes downregulated and 666 genes upregulated by 1.25-fold or greater (FDR < 0.1) specifically in PJ34-iICN1 treated with DOX but not in control PJ34Tet3 cells treated with DOX (Supplemental Table 7). NOTCH1 ChIP-seq experiments identified 357 unique genes in PJ34-iICN1 bound by NOTCH1 at one or more loci in their gene promoters or gene bodies after DOX induction (Supplemental Table 8). Venn diagrams illustrating the overlap of genes regulated by iICN1, JAG, and bound by ICN1 appear in Figure 3, F and G, with intersecting genes listed in Supplemental Tables 9 and 10. *AXL*, *CTNNAL1*, *ITGA3*, *ITGA5*, *LAMC1*, and *LAMC2* were all significantly downregulated by both iICN1 and JAG1 in PJ34, but only *LAMC2* was bound by ICN1 (e.g., within the gene body) in ChIP-seq experiments, suggesting the majority of observed changes linked to early differentiation were an indirect but early effect of NOTCH1 activation.

Genes from the hairy and enhancer of split (HES) and hairy/enhancer-of split with YRPW motif (HEY) family of transcriptional repressors are key canonical downstream targets of NOTCH1 signaling cascades that were also found to be elevated after ICN1 induction and identified through NOTCH1 ChIP-seq. Specifically, HES2, HES4, HEY2, and HEYL were all bound by NOTCH1 in their promoter/gene body regions and significantly upregulated by 20-hour iICN1 induction (Supplemental Tables 7 and 8) but showed lower fold changes with prolonged growth on JAG1 incubation (Supplemental Tables 3 and 7). This likely reflects the cyclical nature of HES/HEY transcription following NOTCH1 activation. HES5, on the other hand, was bound by ICN1, and was strongly elevated after ICN1 induction or prolonged JAG1 exposure (Supplemental Tables 3, 7, and 8). In contrast, the early differentiation markers *KRT13* and *KRT4*, and tumor suppressors *EPHA4*, *PDCD4*, and *TXNIP* — all upregulated by prolonged JAG1 exposure — were not identified as direct NOTCH1 targets by ChIP-seq nor were they strongly induced within 20 hours of ICN1 expression (Supplemental Table 10), suggesting these are later events indirectly triggered through NOTCH1 signaling. Collectively, the data support a model through which NOTCH1 activation triggers early, but indirect, suppression of cell adhesion receptors involved in transitioning away from basement membrane attachment, with subsequent upregulation of differentiation markers.

### NOTCH1 signaling drives anchorage-independent growth but fails to increase CSC frequency or promote in vivo tumor growth.

In vitro growth of tumor spheroids in 3-dimensional culture systems employing low-serum medium is frequently used to propagate and quantify CSCs. The loosely attached tumor spheroids that formed in both *NOTCH1*-mutant and *NOTCH1*-WT cell lines after growth on JAG1 or NOTCH1 signaling stained positive for the senescence marker β-Gal when cultivated in media with regular serum concentrations. However, serum is known to cause differentiation of CSCs. Therefore, we engineered some additional cell lines to robustly examine whether NOTCH1 signaling would increase anchorage-independent growth, survival, or expression of CSC markers in the presence of diminished serum, using our DOX iICN1 vector system. *NOTCH1*-null/mutant UMSCC22A cells were engineered to express the Tet3 regulator and iICN1. For FaDu, we used CRISPR-mediated gene KO to first delete endogenous *NOTCH1*, since baseline cl-NOTCH1 levels are normally high, before introducing the Tet3 regulator and iICN1. Titration experiments indicated that physiological levels of cl-NOTCH1 and the characteristic morphological changes and spheroid formation were achieved at doses of 250–500 ng/mL in FaDu-iICN1 (Supplemental Figure 8, A and B). Physiological levels of activated NOTCH1 were achieved at 200–300 ng/mL DOX in UMSCC22A-iICN1, although morphological changes happened at an even lower dose (Supplemental Figure 8, C and D). At these doses of DOX, profound inhibition of growth in 2-dimensional cultures accompanied expression of cl-NOTCH1 in both cell lines as well as PJ34-iICN1 (Supplemental Figure 9, A and B). In the absence of iICN1 infection, DOX failed to induce morphological changes or growth inhibition in any of the control Tet3G cells (Supplemental Figure 9C). DOX-induced iICN1 expression significantly increased the number of tumor orospheres formed from FaDu and UMSCC22A (Supplemental Figure 10, A and B) in suspension cultures maintained with low serum, compared with control Tet3G cells or cultures lacking DOX.

Next, we examined whether increased tumor spheroid survival reflected increased resistance to anoikis (i.e., cell death associated with detachment). Following 48-hour pretreatment with or without DOX, iICN1 induction was significantly protective against anoikis in both FaDu and UMSCC22A compared with cells without DOX or control Tet3G cells (Supplemental Figure 10C). Because anoikis resistance and spheroid growth are both characteristics of CSCs, we examined whether NOTCH1 activation would also increase CSC markers previously associated with HNSCC, including Aldefluor activity, CD133 expression, and SOX2 protein levels. A 48-hour induction of ICN1 failed to increase CD44^+/bright^Aldefluor^+^ cells in both UMSCC22A and FaDu (Supplemental Figure 11), or the percentage and mean fluorescence of CD133-expressing cells (Supplemental Figure 12, A and B). However, *SOX2* gene expression increased an average of 1.4-fold in PJ34 and 183 cells grown on JAG1 and 1.3-fold in PJ34 after ICN1 induction (Supplemental Tables 4 and 7), although *SOX2* was not bound by NOTCH1 in ChIP-seq (Supplemental Table 8), ruling out direct regulation. SOX2 protein was similarly elevated 2- to 4-fold after ICN1 induction in PJ34, FaDu, and UMSCC22A (Supplemental Figure 12C).

Although stem cell markers and tumor spheroid formation can be surrogates for CSCs, the gold standard remains measuring the in vivo tumor-initiating frequency in mice through limiting dilution assays. We reasoned that if NOTCH1 signaling were driving CSC behavior, it would most likely happen in *NOTCH1-*WT tumors that endogenously express activated NOTCH1, like FaDu. Furthermore, for tumors to grow in vivo, stem cells must be allowed to reenter a proliferative state resembling progenitors by turning NOTCH1 signaling off again. To avoid artifacts from non-physiological levels of NOTCH1 activation, we conducted pilot studies to determine the in vivo DOX dose that would be equivalent to an in vitro dose of 400 ng/mL, which induced physiological levels of NOTCH1 signaling in FaDu-iICN1 (Supplemental Figure 8A). Using a reporter cell line engineered to express luciferase from the same inducible promoter as iICN1, a dose of 1 mg DOX by oral gavage in mice led to a fold induction of luciferase equivalent to that induced by 400 ng/mL DOX in vitro (Supplemental Figure 13).

**Figure 4:**
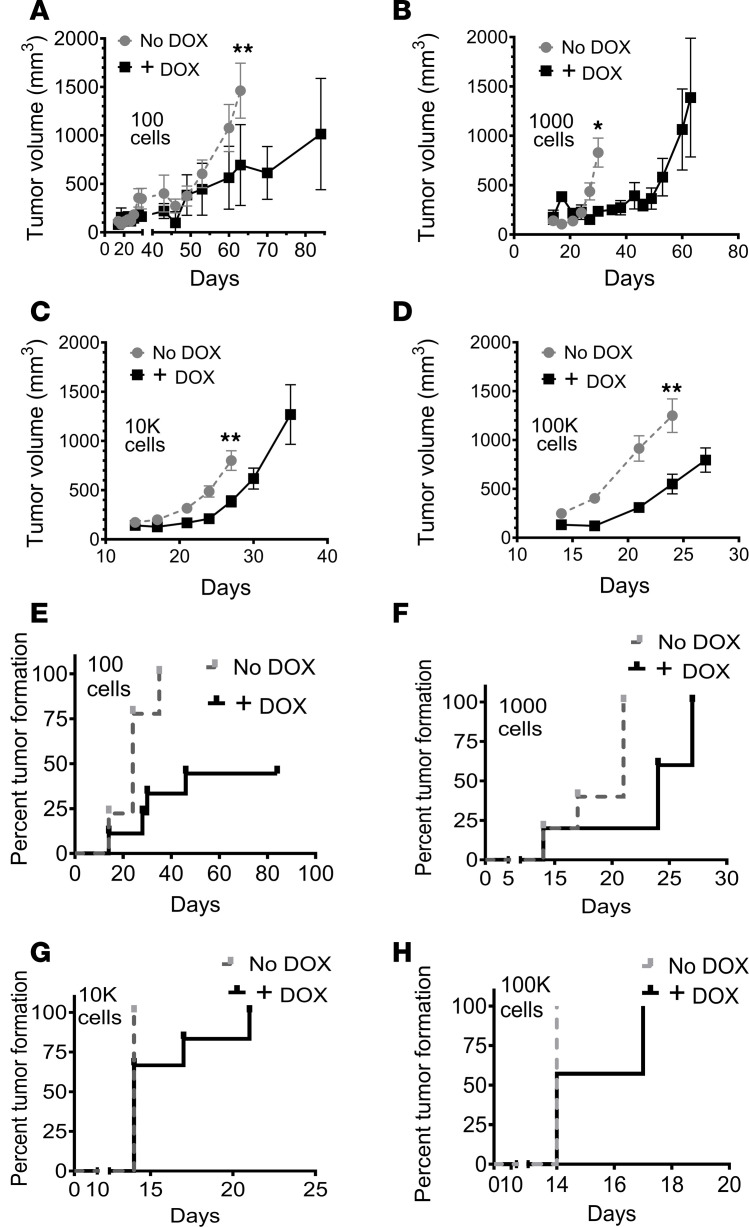
NOTCH1 activation reduces in vivo tumor growth and formation in *NOTCH1*-WT FaDu. After CRISPR-mediated *NOTCH1* KO, FaDu were engineered to express iICN1 and pretreated with or without 300 ng/mL DOX for 72 hours in vitro before injecting 100 cells (**A**), 1000 cells (**B**), 10,000 cells (**C**), or 100,000 cells (**D**) into flanks of mice. Equivalent numbers of untreated *NOTCH1*-KO FaDu were also inoculated and grown in mice receiving no DOX. The DOX-treated group received additional in vivo DOX (1 mg) for 1 week by oral gavage after implantation and tumor growth was plotted verses postinoculation time. DOX-treated tumors (squares) grew considerably more slowly than untreated controls (circles). Time to tumor formation in mice was plotted verses postinoculation time for 100, 1000, 10,000, or 100,000 inoculated tumor cells (**E**–**H**) in the no-DOX groups (gray dotted lines) or DOX-treated groups (black solid lines). Differences in tumor volumes (**A**–**D**) were compared with a 2-sided Student’s *t* test. *P < 0.05; **P < 0.01.

FaDu-iICN1 cells were then treated with 400 ng/mL DOX in vitro for 72 hours to activate NOTCH1 and potentially enrich for CSCs before inoculating increasing amounts of cells (100–100,000 cells range) subcutaneously into mouse flanks. This was followed by daily oral gavage with 1 mg DOX for an additional week to maintain NOTCH1 activation, followed by discontinuation of DOX for the remaining period to allow tumor cells to transition back to a proliferative state. As a control, matching numbers of untreated FaDu-iICN1 cells (e.g., no DOX) were inoculated into mice that never received DOX. Tumor cells treated with DOX grew much more slowly (Figure 4, A–D) and formed tumors later than tumor cells never treated with DOX at every inoculum dose (Figure 4, E–H). Eventually, tumors formed in 100% of animals for all groups except for the lowest inoculum of 100 cells, where only 40% of mice from the DOX group ever formed tumors by 100 days compared with 100% of mice that grew tumors within 40 days when no DOX was given. Statistical analysis estimated a tumor-initiating frequency of 1/189 for DOX treated tumors compared with 1/1 for FaDu with no NOTCH1 induction (Supplemental Table 11, *P* = 0.00142), indicating a drastic reduction in CSC frequency with NOTCH1 activation.

When NOTCH1 signaling was restored in a *NOTCH1*-mutant background using UMSCC22A-iICN, it also profoundly inhibited tumor growth. UMSCC22A-iICN1 were implanted into flanks of nude mice, which were randomized to receive placebo or 1 mg DOX by oral gavage daily for 3 weeks to persistently induce iICN1. NOTCH1 activation profoundly suppressed in vivo tumor growth (Supplemental Figure 14A). After discontinuation of DOX, tumors eventually grew in some mice. Histological staining revealed a roughly 50% reduction in the presence of mouse fibroblasts within the DOX-treated tumor group (*P* < 0.01, Supplemental Figure 14B).

### Suppression of oncogenic AXL and α-catulin expression contributes to NOTCH1-mediated growth inhibition.

NOTCH1 activation increased expression of multiple tumor suppressor genes and simultaneously reduced expression of *AXL* and *CTNNAL1*, which encode 2 oncogenic proteins linked to tumor growth and aggressiveness in HNSCC. We functionally examined the impact of reduced *AXL* and *CTNNAL1* expression on tumor growth in vitro and in vivo, with bicistronic shRNA IRES EGFP constructs targeting these 2 genes. Phenotypes were measured shortly after purifying infected (e.g., EGFP^+^) cells. Specific knockdown of either AXL or α-catulin protein in both *NOTCH1*-mutant HN31 and *NOTCH1*-WT PJ34 was confirmed by Western blotting (Supplemental Figure 15, A, B and G) and found to significantly reduce colony formation in clonogenic assays (*P* < 0.006, Supplemental Figure 15, C and D). Importantly, shRNA knockdown of either *AXL* or *CTNNAL1* severely diminished tumor growth of HN31 in an orthoptic tongue tumor model (Supplemental Figure 15E) and in a subcutaneous flank model using alternate shRNA sequences (Supplemental Figure 15F), demonstrating the phenotype was robust.

We used PJ34-iICN1 and UMSCC22A-iICN1 to functionally examine whether preventing NOTCH1-induced increases in HES/HEY family members would prevent associated decreases in *AXL* and *CTNNAL1* gene expression. In both cell lines, prior knockdown of *HES1*, *HES2*, *HES4*, *HEY1*, or *HEY2* with siRNA shortly before ICN1 induction reduced their elevation stemming from NOTCH1 activation but did little to prevent associated reductions in *AXL* and *CTNNAL1* expression (determined by quantitative real-time PCR [qPCR], Supplemental Figure 16). Furthermore, combined simultaneous knockdown of some of the more strongly induced NOTCH1 targets, *HES5/HEY1/HEY2*, also failed to prevent NOTCH1-induced decreases in *AXL* and *CTNNAL1* expression in both PJ34 and UMSCC22A (data not shown).

### A gene expression signature identifies primary HNSCC tumors with intact NOTCH1 signaling and an altered tumor microenvironment.

**Figure 5:**
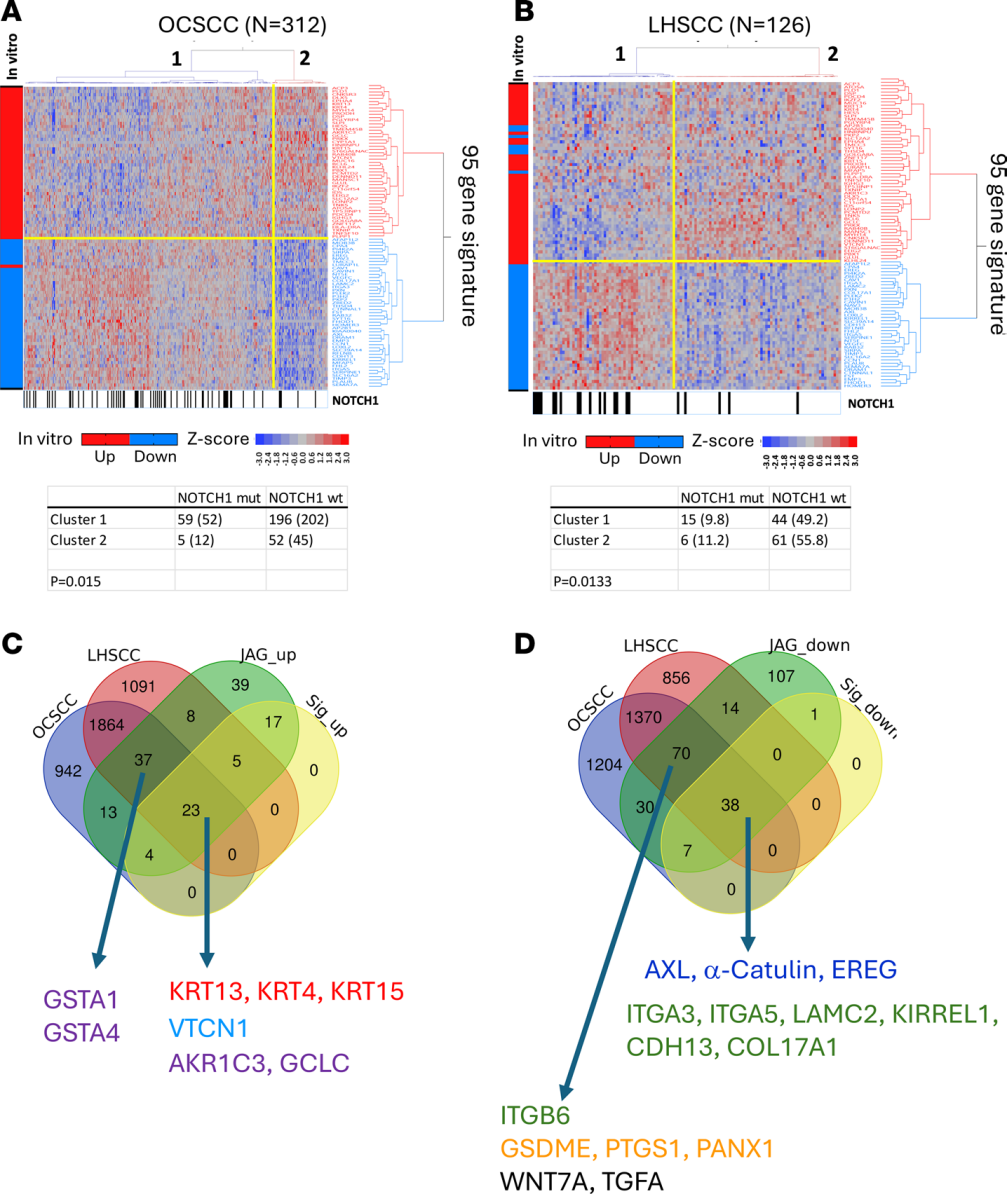
Comparison of genes regulated by NOTCH1 in vitro and genes differentially expressed in primary HNSCC tumors with a NOTCH1 activation signature. (**A**) Consensus hierarchical clustering of TCGA OCSCC primary tumors based on a 95-gene NOTCH1 activation signature identified a cluster of patient tumors (Cluster 2, *N* = 57) with an expression pattern indicative of active NOTCH1 signaling and another cluster (Cluster 1, *N* = 255) predicted to have loss of NOTCH1 signaling. Genes are annotated with vertical boxes according to whether they were upregulated (red) or downregulated (blue) by JAG1 in vitro. Samples with a *NOTCH1* mutation are annotated horizontally with a black box and association between *NOTCH1* mutation status and cluster for patients with sequencing information was analyzed by χ^2^ analysis. (**B**) Parallel clustering and analysis of TCGA LHSCC primary tumors using the same 95-gene NOTCH1 signature. (**C**) Venn diagram illustrating overlap of genes upregulated (FDR < 0.1, |fold change| ≥ 1.25) by NOTCH1 in vitro (JAG_up), the subset of upregulated genes part of the NOTCH1 signature (Sig_up), and genes upregulated (FDR < 0.1, |fold change| ≥ 1.25) in Cluster 2 from OCSCC or LHSCC. (**D**) Venn diagram illustrating overlap of genes downregulated (FDR < 0.1, |fold change| ≥ 1.25) by NOTCH1 in vitro (JAG_down), the subset of downregulated genes part of the NOTCH1 signature (Sig_down), and genes downregulated (FDR < 0.1, |fold change| ≥ 1.25) in Cluster 2 from OCSCC or LHSCC.

A robust in vivo gene expression signature of NOTCH1 activation was developed based on the top 120 differentially regulated genes identified in vitro after JAG1 stimulation (Supplemental Figure 5) by examining their cross-correlation in primary tumors from The Cancer Genome Atlas (TCGA) oral squamous cell carcinoma (OSCC) cohort of 312 patients. After removing one gene for low expression, 2-way hierarchical clustering of cross-correlation coefficients identified 2 primary gene clusters or modules (Supplemental Figure 17A) from TCGA data. Genes from each cluster showed a dominant but opposite pattern of JAG1 regulation in vitro, identifying up- and downregulated gene groups. After removal of several inconsistently regulated genes, 95 genes remained in the final signature (Supplemental Figure 17B and Supplemental Table 12). We applied the 95 gene signature to perform consensus hierarchical clustering on the TCGA OCSCC and TCGA laryngeal\hypopharyngeal SCC (LHSCC) cohorts individually. Consensus clustering metrics identified that the choice of 2 sample clusters was optimal for both TCGA cohorts (Supplemental Figure 18). After 2-way clustering (Figure 5, A and B), it was predicted that NOTCH1 signaling is off in sample cluster 1 but on in sample cluster 2 for both OCSCC and LHSCC cohorts, based on the direction of gene regulation from the 95-gene signature observed (Supplemental Table 13 and 14). The predicted NOTCH1 pathway states were compared to the *NOTCH1* mutational status of samples in both cohorts to validate the NOTCH1 signature. In both cases, sample cluster 2 was depleted for *NOTCH1* mutations but sample cluster 1 was enriched (*P* = 0.015 and *P* = 0.013, Figure 5, A and B), consistent with inactivating *NOTCH1* mutations preventing NOTCH1 pathway signaling. Consequently, the gene signature likely distinguished tumors based on their NOTCH1 pathway status, and the data suggest that NOTCH1 signaling is active in a subset of OCSCC and LHSCC tumors (e.g., sample cluster 2).

Next, we identified all genes differentially expressed by the subset of TCGA tumors predicted to have NOTCH1 activated in the OCSCC (Supplemental Table 15) and LHSCC (Supplemental Table 16) cohorts and compared them to genes found differentially regulated by JAG1 binding in vitro, including the subset of 95 genes defining the pathway signature. Venn diagrams depicting overlap of differentially expressed genes appear in Figure 5, C and D. Among the commonly upregulated genes (Supplemental Table 17) were early differentiation markers *KRT13*, *KRT14*, and *KRT15*; the immune checkpoint *VTCN1*; and several enzymes involved in the antioxidant response, including *AKR1C3*, which neutralizes lipid peroxides, as well as *GCLC*, *GSTA1*, and *GSTA4*, which are essential for glutathione synthesis and antioxidant response. Among commonly downregulated genes (Supplemental Table 18) were multiple integrins and cell adhesion molecules *ITGA3*, *ITGA5*, *ITGB6*, *LAMC2*, *KIRREL1*, *CDH13*, including *COL17A1* (collagen XVII) that mediates adhesion to basement membranes as a component of hemidesmosomes. The pro-oncogenic genes *AXL* and *CTNNAL1* were also commonly downregulated. So was *WNT7A*, which contributes to HNSCC growth by stimulating β-catenin, as well as *EREG* and *TGFA*, both EGFR ligands that promote HNSCC.

**Figure 6:**
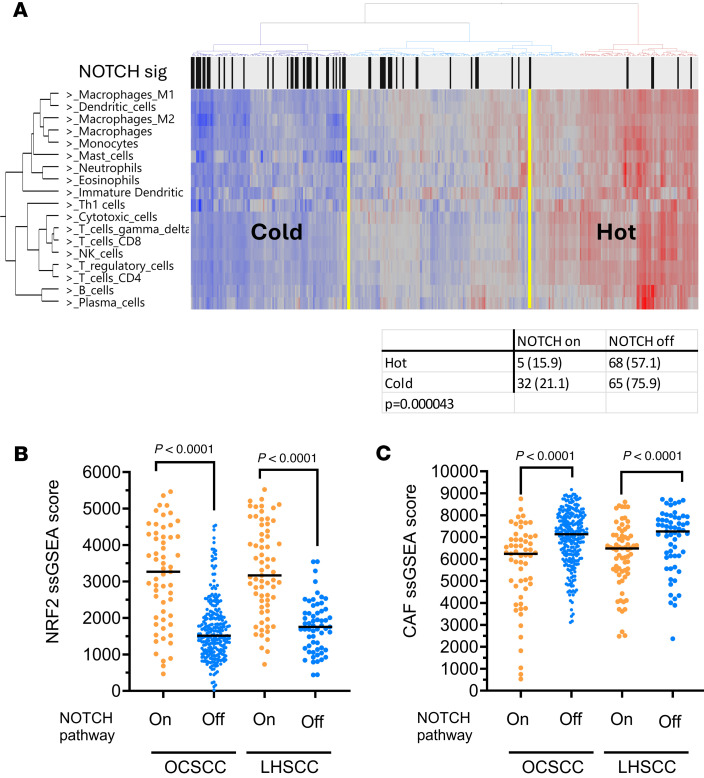
NOTCH1 activation in primary HNSCC is associated with changes to the tumor microenvironment. (**A**) ssGSEA scores representing 18 different leukocyte subsets derived from TCGA OCSCC were used for hierarchical clustering to classify samples as immunologically cold (sample cluster 1) or hot (sample cluster 3) and the membership of samples from previous clustering based on the NOTCH1 gene signature is annotated with a black box for NOTCH1 signaling on or a gray box for NOTCH1 signaling off. Tumors with activated NOTCH1 are depleted from immunologically hot tumors and enriched in cold tumors by χ^2^ analysis (*P* < 0.0001). (**B**) NRF2 pathway activation scores are significantly higher among OCSCC and LHSCC tumors when NOTCH is activated. (**C**) CAF pathway scores are significantly higher in OCSCC and LHSCC tumors where NOTCH signaling is turned off. Differences in NRF2 pathway scores were analyzed with a 2-sided Student’s *t* test (**C** and **D**).

Single-sample gene set enrichment analysis (ssGSEA) was used to determine associations between the tumor microenvironment and NOTCH1 signaling, using published lists (Supplemental Table 19) specific for immune subtypes, endothelial cells, and a robust gene list we constructed for cancer-associated fibroblasts (CAFs). Our CAF signature was derived by clustering cross-correlation coefficients for fibroblast-associated genes across more than 9000 solid tumors from TCGA. In the TCGA OCSCC cohort, tumors with a NOTCH1 activation signature had significantly reduced proportions of nearly every immune subset analyzed (Supplemental Table 20), indicating they are immunologically “cold.” Two-way hierarchical clustering with the immune subset ssGSEA scores demonstrated significant depletion of tumors with active NOTCH1 signaling among OCSCC “hot” tumors (*P* < 0.0001, Figure 6A). In LHSCC samples with NOTCH1 activation there was a similar trend of broad decrease in leukocytes subpopulations present when NOTCH1 signaling was on, although differences only reached statistical significance for T helper type 1 and mast cells (Supplemental Table 21). Because we previously showed that a cold immune microenvironment was associated with elevated NRF2 gene signatures specifically in OCSCC but not LHSCC (32), we revisited the connection between NOTCH1 signaling and the antioxidant response inferred from some of the top genes commonly regulated by NOTCH1 (Figure 5C) or proteins correlating with cl-NOTCH1 (Supplemental Table 2). HNSCC TCGA tumors with NOTCH1 activation had NRF2 pathway scores (Figure 6B) that were on average profoundly elevated regardless of disease subsite (*P* < 0.0001). In contrast, NOTCH1 activation was associated with a significant reduction in the proportion of CAFs present, imputed from CAF ssGSEA scores in both OCSCC and LHSCC (Figure 6C), which was supported by gross differences in fibroblast content visible in H&E images downloaded from the TCGA project (Supplemental Figure 19).

### NOTCH1 signaling correlates with survival and PIK3CA genomic changes.

**Figure 7:**
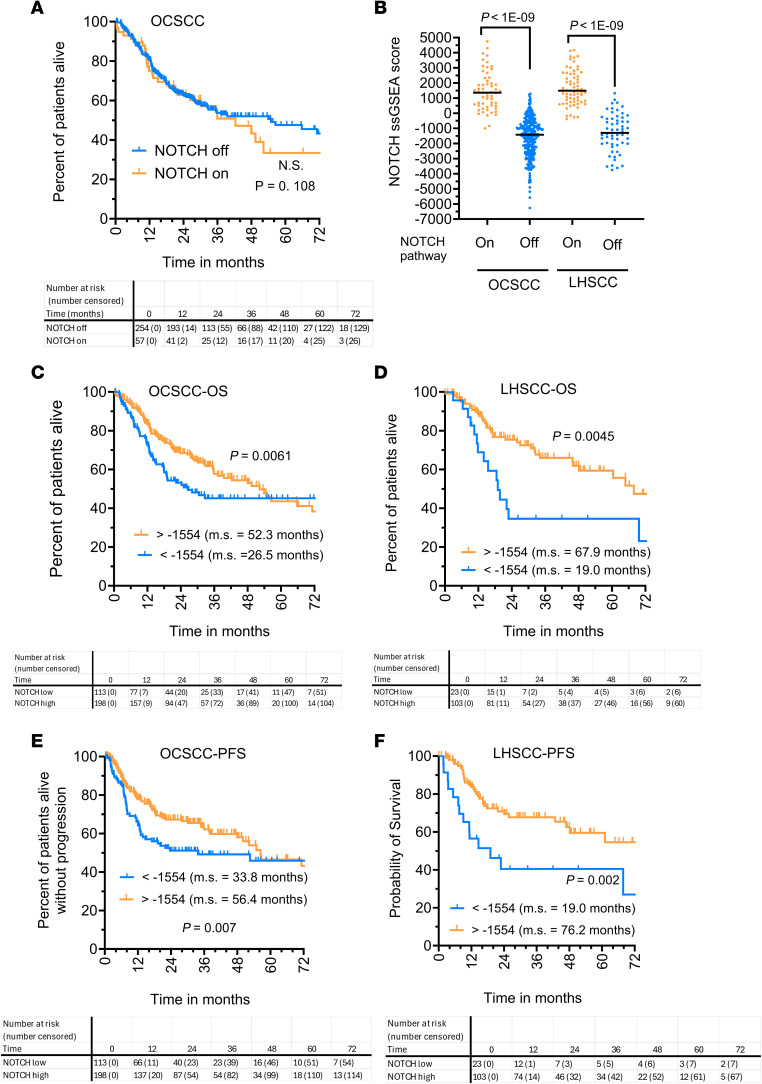
Higher levels of NOTCH1 activation correlate with better survival in OCSCC and LHSCC TCGA cohorts. (**A**) No difference in OS among TCGA OCSCC patients when NOTCH1 activation is treated as a categorical variable based on clusters with the NOTCH gene signature. (**B**) Validation that ssGSEA scores derived from the NOTCH1 gene signature provide a continuous value measurement that faithfully replicates sample clustering. (**C**) OCSCC samples with higher ssGSEA scores (e.g., NOTCH1 signaling) above a threshold (–1554) determined by optimal cutpoint selection have significantly improved OS. (**D**) LHSCC samples with higher ssGSEA scores above the same threshold (–1554) have significantly improved OS, validating the threshold. (**E**) OCSCC patients with higher NOTCH1 signaling have improved PFS. (**F**) LHSCC patients with higher NOTCH1 signaling have improved PFS. At-risk tables underneath Kaplan-Meier curves indicate the number of patients still at risk or censored at the indicated time intervals. *P* values for survival curves were determined with a log-rank test.

When NOTCH1 signaling status was treated as a dichotomous variable for OCSCC TCGA samples based on clustering, there was no significant association (Figure 7A) with overall survival (OS). Because signaling pathways are rarely binary, we used the NOTCH1 gene signature to generate ssGSEA scores for samples and treated NOTCH1 signaling as a continuous variable, which demonstrated clear separation between sample clusters found previously (Figure 7B). Optimal dichotomization identified a NOTCH1 ssGSEA threshold of –1554 that stratified OCSCC patients into 2 groups that differed significantly by survival, with poor survival corresponding to lower levels of NOTCH1 signaling (*P* = 0.0061, Figure 7C). The threshold identified seemed biologically meaningful, as it corresponded to roughly the average value for samples from the NOTCH1-off clusters in both OCSCC and LHSCC (Figure 7B), and it also separated the LHSCC samples into 2 groups that differed in survival in the same manner (*P* = 0.0.0045, Figure 7D). Likewise, patients with NOTCH1 ssGSEA scores below this same threshold had significantly worse progression-free survival (PFS) in both disease subsites (Figure 7, E and F). Using an independent cohort of OCSCC tumors (*N* = 43) previously characterized by our group (2) but profiled for gene expression using a microarray platform, we were able to validate that NOTCH pathway activation was associated with better survival (Supplemental Figure 20). Patients whose tumors had low NOTCH scores had a median survival time that was roughly half of that for those with tumors having a high NOTCH score. No associations were found between lymph node stage, tumor stage, or smoking history in either disease subsite, although there was a significant decline in NOTCH signaling levels among poorly differentiated tumors found in OCSCC tumors (not shown).

The improved survival associated with higher NOTCH signaling in tumors conflicts with what would be predicted based on their higher levels of NRF2 signaling (e.g., Figure 6B), which itself is associated with worse survival and a colder tumor immune microenvironment (32). To disentangle the relationships between NOTCH, NRF2, leukocyte infiltration, and survival, we plotted NRF2 versus NOTCH scores in TCGA OCSCC tumors, confirming a positive correlation (Supplemental Figure 21A). An overlay of the pathway thresholds defined 4 quadrants or risk groups (Supplemental Figure 21A). As predicted, tumors annotated by immunological status above the NRF2 threshold tended to be immunologically colder. Kaplan-Meier plots (Supplemental Figure 21B) revealed that patients whose tumors had high NRF2 and low NOTCH signaling (quadrant 1) had the worst median survival time (15.2 months). Patients whose tumors had low NRF2 and high NOTCH scores had the best prognosis with a median survival of 71.2 months, with patients in the remaining 2 quadrants showing an intermediate and similar survival phenotype. A Cox proportional hazards model fit of survival time demonstrated independent contributions of both NRF2 (*P* = 0.008) and NOTCH (*P* = 0.0206) pathway status with no significant interaction (Supplemental Table 22). Having either a low NOTCH score or high NRF2 score led to an increased chance of death, with hazard ratios (HRs) of 1.24 and 1.3, respectively.

Next, we examined the relationship between these pathways and immunological status of OCSCC tumors in a nominal logistic regression model (Supplemental Table 23). NRF2 was the dominant factor driving colder tumors (*P* < 0.0001) with an odds ratio of 5.08 (per 1000 units of NRF2 score, *P* < 0.0001), but NOTCH score did have a significant and smaller independent effect (*P* < 0.03) with an odds ratio of 0.78 (per 1000 units of NOTCH score, *P* < 0.05), signifying that lower NOTCH score (e.g. NOTCH off) was associated with hotter tumors.

We previously reported that HNSCC cell lines harboring *NOTCH1* loss-of-function mutations are exquisitely sensitive to PI3K inhibitors (28, 29), which was supported by a small clinical trial we conducted (30). Direct links between NOTCH1 signaling and PI3K inhibitor sensitivity proved context dependent, leading us to hypothesize that tumors evolving with *NOTCH1* mutations may have altered pathway dependencies, including PI3K. We analyzed the relationship between TCGA NOTCH1 sample clusters and found that the presence of PIK3CA genomic changes (e.g., high-level copy gains or mutations) was significantly enriched among OCSCC (*P* = 0.024) and LHSCC samples (*P* = 0.0004) belonging to the NOTCH-on clusters (Supplemental Figure 22).

## Discussion

More than a decade has passed since our group and others first identified inactivating *NOTCH1* mutations as a driver of HNSCC (1, 3). Yet, the function and significance of NOTCH1 signaling in this cancer are still poorly understood. Adding to the complexity, WT *NOTCH1* has been reported as an oncogenic driver inducing CSC-like properties in some HNSCC cell lines (7, 27), with strong NOTCH1 signaling observed in subsets of primary tumors. Here, we clarify these conflicts and unify the field.

Regardless of *NOTCH1* mutational status or endogenous levels of signaling, the pathway activates a program of very early differentiation that involves downregulation of cell adhesion molecules, which normally tether cells to their basement membrane, accompanied by upregulation of keratin differentiation markers. These changes in adhesion accompanied by increased anoikis resistance, which is likely a vestige of squamous epithelial stratification, promotes growth of cells in a non-adherent spheroid state and likely explains reports that NOTCH1 signaling triggers HNSCC tumors to become more CSC like (19, 25, 26). However, using an inducible cl-NOTCH1 expression system in *NOTCH1*-WT FaDu clearly showed a substantial decrease in tumor-initiating cells in vivo using the gold standard limiting dilution assay. Furthermore, we found that the NOTCH1 inhibitor DBZ, more potent and specific than DAPT frequently used in prior NOTCH studies (16, 23, 33), had no effect on in vitro growth of 6 different HNSCC tumor lines specifically chosen for high basal cl-NOTCH1expression. Collectively, this refutes the idea that NOTCH1 actively drives cell proliferation in HNSCC. Interestingly, we find evidence of limited NOTCH1 signaling in HNSCC cell lines with high basal cl-NOTCH1 expression, given the strong anticorrelation with AXL protein — one of the genes robustly downregulated by NOTCH1 activation. That said, no obvious growth phenotypes distinguished cell lines with high NOTCH1 signaling, but when the gene was knocked out in FaDu and cl-NOTCH1 reexpressed at physiological levels there was a dramatic morphologic transformation to tumor spheroid growth not otherwise observed for parental FaDu with equivalent NOTCH1 signaling. Expression of Fbxw7 protein, the E3 ligase known to degrade intracellular activated NOTCH1, strongly correlated with cl-NOTCH1 protein by RPPA in cell lines. Collectively, our data suggest that some HNSCC cell lines can tolerate NOTCH1 signaling in vitro and that growth in 2-dimensional cultures may select for diminished downstream phenotypes.

By defining genes altered after physiological activation of NOTCH1 in vitro, we were able to construct an empirically derived gene expression signature of NOTCH1 activation that was validated in primary HNSCC tumors from 2 different disease sites, allowing stratification of patients’ samples based on relative levels of NOTCH1 signaling. Wholesale downregulation of cell adhesion receptors, particularly integrins along with genes encoding extracellular matrix proteins such as laminins and COL17A1 were found to accompany NOTCH1 activation in both preclinical models and primary tumors, consistent with very early differentiation of tumors that mirrors transition from the basal to suprabasal layer in normal mucosa. Possibly, the shift in cell adhesion and extracellular matrix proteins could impact the tumor microenvironment and contribute to the diminished presence of CAFs. We found evidence of this in our preclinical model and in both disease subsites when NOTCH1 signaling was elevated. A surprising finding was the association between NOTCH1 activation and increased NRF2 pathway signaling, which has implications for chemoradioresistance, and is consistent with reports that NOTCH1 signaling increases chemotherapy resistance (25, **26**). Elevated NRF2 activity contributes to cisplatin resistance using preclinical models (34, 35) and elevated NRF2 activity is associated with an immunologically cold tumor immune microenvironment in multiple tobacco-associated tumors (32), including OCSCC but not LHSCC. Consistent with this, we found NRF2 scores were significantly correlated with NOTCH scores. Both the NRF2 and NOTCH pathways independently associated with worse survival, defining 4 distinct risk groups in which patients whose tumors were NRF2 high/NOTCH low had the poorest outcomes of all groups. While both high NOTCH and NRF2 activation were significantly associated with immunologically cold tumors, the effects of NRF2 were considerably stronger.

Many genes altered by NOTCH1 were modulated within an early time frame but were not directly regulated by binding of ICN1 to their promoters or enhancers. Of these, *AXL* (36, 37) and *CTNNAL1* (38, 39) reportedly contribute to HNSCC tumor growth in preclinical models and are associated with clinical aggressiveness. We confirmed their pro-oncogenic function in vitro and in vivo through knockdown experiments and they contributed to some of the growth inhibition triggered by NOTCH1 activation. However, the multiplicity of genes commonly regulated by NOTCH1 both in vitro and in vivo suggests cooperative gene expression programing with possibly redundant function. *SOX2* was the one gene tied to CSC that we and others found upregulated by NOTCH1 activation (24, 40). However, IHC has shown that SOX2 (41) along with cl-NOTCH1 (7) is frequently present in the normal mucosal suprabasal layer and elevated SOX2 correlates with better HNSCC prognosis (41), consistent with our proposed model of very early differentiation.

If NOTCH1 signaling turns on a program of very early differentiation and is not associated with CSC maintenance, then we might expect tumors with higher NOTCH1 signaling to have a better prognosis. Consistent with what others reported for cl-NOTCH1 staining (27), we found that subsets of tumors with higher NOTCH1 signaling scores had improved OS and PFS across disease subsites. Our preclinical models demonstrated that *NOTCH1*-WT tumors retain plasticity and can undergo very early differentiation in response to NOTCH1 signaling. This is consistent with prior work proposing that *NOTCH1* loss-of-function mutations drive carcinogenesis by preventing early stem cell differentiation and promoting accumulation of secondary mutations in an expanding pool of stem cells (42). Possibly, precancerous lesions arising with *NOTCH1* mutations avoid early differentiation and retain some pathway dependencies of stem cells that may include the PI3K pathway, which has been linked to survival and maintenance of CSCs (43). This could explain the sensitivity to PI3K inhibitors associated with *NOTCH1* mutations we reported in the absence of *PIK3CA* mutations (28). If early differentiation driven by NOTCH1 activation in WT tumors were accompanied by a shift away from the PI3K pathway, then perhaps these tumors rely more on genomic changes in the *PIK3CA* gene to reestablish signaling. This is supported by our findings that mutations and high-level amplifications of the *PIK3CA* gene are more frequent in tumors with higher NOTCH1 signaling. Nevertheless, *NOTCH1* mutations sometimes do co-occur in HNSCC tumors harboring PIK3CA mutations (4). We have also seen this in a few established HNSCC cell lines (28), and it has been reported by others that *NOTCH1* deletion accelerates growth of genetically engineered mouse models (GEMMs) of HNSCC driven by activating *PIK3CA* mutations (44). While the trend toward mutual exclusivity may reflect steps in carcinogenesis, evidence from GEMM studies supports the notion that loss of *NOTCH1* function can still be advantageous in HNSCC tumors driven by PIK3CA oncogenes.

A dual oncogenic/tumor suppressive function for *NOTCH1* has been reported in HPV^+^ tumors in HNSCC GEMMs (45) driven by expression of HPV-derived E6/E7 oncogenes plus an activating KRAS mutation (KHR mice), where faster tumor growth occurred when KHR mice expressed activated ICN1 or lost the *NOTCH1* gene. However, the authors noted that KHR tumors had impaired differentiation persisting with *NOTCH1* changes, making extrapolation to our work difficult, as this genomic background may obscure differentiation pathway changes. The same study showed that in HPV^–^ GEMMs with mutated KRAS, homozygous *NOTCH1* loss accelerated tumor growth — supporting a tumor suppressor function, consistent with conditional *NOTCH1* KO accelerating cSCC formation in mice (12, 46). One *NOTCH1*-mutant line in our study (UMSCC47) is HPV^+^, and NOTCH1 restoration inhibited colony formation similarly to HPV^–^ lines. NOTCH pathway scores showed a trend toward worse survival in HPV^+^ TCGA HNSCC patients (not shown), possibly because HPV oncogenes interfere with differentiation programs, reducing reliability of downstream target-based tumor separation. The paucity of *NOTCH1* mutations among HPV^+^ HNSCC tumors (4) suggests these cancers escape early differentiation through E6/E7 transformation rather than *NOTCH1* loss, with little evidence of activating *NOTCH1* mutations in HPV^+^ tumors.

How then do we reconcile our conclusions with multiple reports of NOTCH1 behaving like an oncogene in HNSCC? Many factors likely contribute, including overinterpretation of tumor spheroid properties in this context, frequent use of antibodies that detect inactive membranous/cytoplasmic NOTCH1 rather than active cl-NOTCH1 for IHC studies, nonspecific pharmacological inhibitors to block NOTCH1 signaling, widely available constructs to overexpress activated NOTCH1 that cannot be validated with cleavage-specific antibodies, and/or ambiguous use of antibodies recognizing C-terminal NOTCH1 peptides in lieu of those specific for activated NOTCH1. In summary, we find molecular evidence of NOTCH1 signaling in subsets of HNSCC tumors that has broad gene expression consequences impacting tumor biology, the tumor microenvironment, and clinical behavior. However, most of the evidence unequivocally supports a tumor suppressor function for *NOTCH1*, regardless of mutational status or baseline NOTCH1 signaling, which triggers early differentiation accompanied by decreased cell attachment.

## Methods

### Sex as a biological variable.

Male mice were used, as this reflects the sex prevalence of HNSCC (75% male, 25% female). Sex was not considered a biological variable, as the genes tested are not linked to hormonal pathways and results were expected to be similar in female mice.

### Cell lines, plasmids, and reagents.

The established HNSCC cell lines used in experiments were obtained from MD Anderson and are listed in Supplemental Table 1. Cells were passaged in growth media containing 10% FBS plus additives, validated by STR profiling, and profiled for somatic mutations as previously described (47). NFL1 cDNA was obtained from OriGene, the ICN1 retroviral construct encoding activated human *NOTCH1* was obtained in-house, and the cDNA encoding human cleaved *NOTCH1* detectable by commercial cl-NOTCH1 antibodies was subcloned through reverse transcriptase PCR using RNA derived from *NOTCH1*-WT FaDu cells. Details regarding these constructs, shRNAs targeting *AXL* and *CTNNAL1* and CRISPR vectors, and siRNA reagents targeting *HES/HEY* family members are provided in Supplemental Methods. Catalog numbers for antibodies obtained from Cell Signaling Technologies and Santa Cruz are provided in Supplemental Methods as well.

### NOTCH activation, clonogenic assays, Western blots, and RPPAs.

For NOTCH activation experiments, tissue culture wells were precoated with immobilized recombinant JAG1 fused to an Fc fragment or control IgG Fc protein as described in Supplemental Methods. Proteins lysates were harvested, resolved by SDS-PAGE, electro-transferred to PVDF membranes, and probed with specific antibodies using standard methods, as previously described (34). Antibodies against cl-NOTCH1, total NOTCH1, total NOTCH2, AXL, and FLAG tag were from Cell Signaling Technology; whereas antibodies against α-catulin, LAMC2, ITGA3, and ITGA5 were from Santa Cruz Biotechnology and anti-HES5 was from Abcam. For clonogenic assays, 1000 cells were seeded into replicate 6-well plates either uncoated or pretreated with JAG1 or control Fc, grown for 7–10 days, fixed and stained with 0.5% crystal violet, and the number of colonies with greater than 60 cells were counted with ImageJ software (28). RPPAs were used to quantitate levels of 157 different protein/phosphoproteins using lysates prepared from a panel of HNSCC cell lines and validated antibodies according to methods we previously published (1). Pearson’s correlations with cl-NOTCH1 protein were calculated using JMP v19 (SAS) and *P* values were adjusted with a Benjamini-Hochberg (B-H) correction (FDR = 0.1, significance cutoff).

### Differentially expressed gene analysis and RNA-seq.

After growing cells on plates coated with either JAG1 or Fc control protein (in biologic triplicate) for 5 days, extracted RNA was processed by the MD Anderson Sequencing and Microarray Core Facility and individually quantitated following hybridization to Affymetrix HuGene 2.0 ST arrays. RNA expression after iICN1 induction in PJ34-iICN1 cells was determined by RNA-seq using replicate samples incubated with or without 1000 ng/mL DOX for 36 hours. Detailed bioinformatics analyses, including DEG identification, consensus hierarchical clustering, and comparison to TCGA RNA-seq data from 423 HNSCC patients are described in Supplemental Methods. Human TCGA data were obtained from the BROAD firehose portal (https://gdac.broadinstitute.org/). Human OCSCC validation data and clinical information were obtained from the NCBI GEO public repository (GSE41116) and accompanying publication (PMID 23619168).

### qPCR.

RNA (2–5 mg) was converted to cDNA using a SuperScript First Strand synthesis Kit (Life Technologies) and 100 ng of cDNA was added to quadruplicate reactions containing FAM-MGB TaqMan PCR primers (Supplemental Methods) and amplified in a Bio-Rad c1000 Thermal Cycler. Expression of targets was normalized to GAPDH by the ΔCt method using CFX Manager 3.1 software (Bio-Rad).

### Staining for β-Gal.

Cells were fixed and stained with a β-Galactosidase Kit (Cell Signaling Technology), according to the supplied instructions and 5 random 10× objective fields were observed to count the number of positive and negative staining cells. For quantitating spheres, 25 random fields were counted.

### Mouse tumor models and CSC frequencies.

Tongue orthotopic and subcutaneous flank tumor models were established in male nude mice (10 per group) using shRNA-expressing or iICN1-inducible cell lines, with DOX administered by oral gavage where indicated (see Supplemental Methods for details). CSC frequencies in untreated and DOX-treated FaDu-iICN1 populations were estimated by limiting dilution using the ELDA software tool (48) at https://bioinf.wehi.edu.au/software/elda/

### Statistics.

Log_2_ transformation was used for all count data and the logit transformation for all percentage data before statistical testing. GraphPad Prism or JMP v13 (SAS) statistical software was used for most analyses. Two-sided *t* tests were employed for comparisons involving only 2 groups (*P* < 0.05 threshold for 2 groups), whereas experiments with multiple groups were analyzed using analysis of variance (ANOVA, *P* < 0.05 significance cutoff). For the latter, a post hoc Tukey’s test was applied for pairwise comparisons, or Dunnett’s test was used when comparing groups against a control treatment (*P* < 0.05 significance cutoff). ssGSEA scores were calculated through the BROAD Institute’s Gene Pattern public server at https://www.genepattern.org/ using published lists specific to individual cell types that we vetted through cross-correlation of gene expression across more than 9,000 solid tumor samples from TCGA. GO enrichment was performed through the GO website portal at https://geneontology.org/, which utilizes a hypergeometric/Fisher’s exact test and corrects *P* values for multiple testing using a B-H correction (FDR < 0.1 as the significance cutoff). Survival data were analyzed through a Cox’s proportional hazards model (JMP v19), treating NOTCH and NRF2 score groups as binary nominal variables with a *P*-value threshold of less than 0.05 for the likelihood ratio tests. The relationship between tumor immunological status and the continuous variables NOTCH and NRF2 scores was analyzed with a nominal logistic fit model (JMP v19) using a *P*-value cutoff of 0.05 for Wald’s χ^2^ tests (parameter estimates) and likelihood ratio tests (effects). Optimal cutpoint selection to dichotomize continuous variables for survival analysis was done with an in-house Python script (https://github.com/Mjfreder/Partition_survival_analysis) that used a data-driven approach to search over all observed values to maximize the log-rank test statistics. Colony counts (square root–transformed) were analyzed using a cell means model, fitting all cell line × treatment combinations as a single factor in a 1-way ANOVA. Planned contrasts tested the simple effect of JAG versus Fc within each cell line and the interaction between treatment and cell line genotype (each KO versus parental), with Bonferroni’s correction applied to the interaction contrasts.

### Ethics approval and consent to participate.

The research did involve any human participants. All human data or cell lines used are publicly available and therefore no consent is required.

All animal experiments were performed in accordance with protocols (AN-7317, AN-7321) and ethics approved by our Institutional Animal Care and Use Committee at Baylor College of Medicine and The University of Texas MD Anderson Cancer Center.

### Data availability.

All human clinical specimen data used, including gene expression, are available from public databases. Raw harmonized TCGA RNA-seq data were downloaded from the University of Santa Cruz Xena browser at https://xenabrowser.net TCGA genomic and clinical data were obtained from cbioportal website at https://www.cbioportal.org/ Normalized RNA microarray expression data from the MD Anderson validation was downloaded from the GEO database (GSE41116) and the data with converted gene symbols are available in the supplemental tables. Normalized microarray and RNA-seq data from experiments for individual genes across replicate samples are available in the primary supplemental tables. Bulk RNA-seq was performed for this study more than 5 years ago. The original raw sequencing files (FASTQ) are no longer available due to data archiving limitations at the time the experiments were conducted. Processed gene expression matrices and all data used for analysis are provided in the Supporting Data Values file.

All cell line models engineered will be made available upon request.

## Author contributions

The co–first authors (CH, SM, and QL) contributed equally. Author order was determined by the senior author (MJF) based on overall contribution to project completion: CH completed critical experimental gaps, including in vivo studies. SM performed foundational experiments that enabled subsequent work. QL generated all CRISPR KO models and conducted validation experiments. Collectively, their contributions were deemed equivalent in scientific contribution and importance. Investigation: CH, SM, QL, KMA, KS, DD, Jiping Wang, ZL, NT, RS, TXX, AAO, KR, JVH, LAB, VCS, PY, and MJF. Formal analysis: XR, JZ, YX, Jing Wang, DAW, ES, AKEN, and MJF. Writing-original draft: CRP, PAZM, FMJ, VCS, JNM, PY, and MJF. Resources: PAZM, including the ICN1 retroviral construct encoding activated human *NOTCH1*. Funding acquisition: FMJ, JNM, and MJF. Conceptualization, Data Curation, Methodology, Project administration, Supervision: MJF.

## Conflict of interest

VCS is a consultant for Femtovox Inc. This material is based on work supported in part by the Department of Veterans Affairs, Veterans Health Administration, Office of Research and Development. The views expressed in this article are those of the authors and do not necessarily reflect the position or policy of the Department of Veterans Affairs or the US government.

## Funding support

This work is the result of NIH funding, in whole or in part, and is subject to the NIH Public Access Policy. Through acceptance of this federal funding, the NIH has been given a right to make the work publicly available in PubMed Central.

- National Institute of Dental and Craniofacial Research grant R01DE024179 (to MJF).
- National Cancer Institute grants R01CA235620 (to MJF), U54CA274321 (to VCS, MJF, JNM, and AAO), I01BX006380 (to VCS and MJF), and U01DE025181 (to MJF and JNM).
- Cancer Prevention Institute of Texas grant RP200369 (to MJF and FJ).

## Supplementary Material

Supplemental data

Unedited blot and gel images

Supplemental tables 1-14

Supplemental table 15

Supplemental table 16

Supplemental tables 17-23

Supporting data values

## Version 1 04/16/2026

In-Press Preview

## Version 2 06/08/2026

Electronic publication
