# Supplementary material for: NOTCH1 acts as a tumor suppressor that induces early differentiation in head and neck cancer
Source: JCI Insight. 2026 Apr 16;11(11):e202414. doi: 10.1172/jci.insight.202414 (PMC13313506; doi:10.1172/jci.insight.202414)
Supplement: Supplemental data [file jciinsight-11-202414-s185.pdf]

SUPPLEMENTARY FIGURE LEGENDS

Supplementary Figure 1

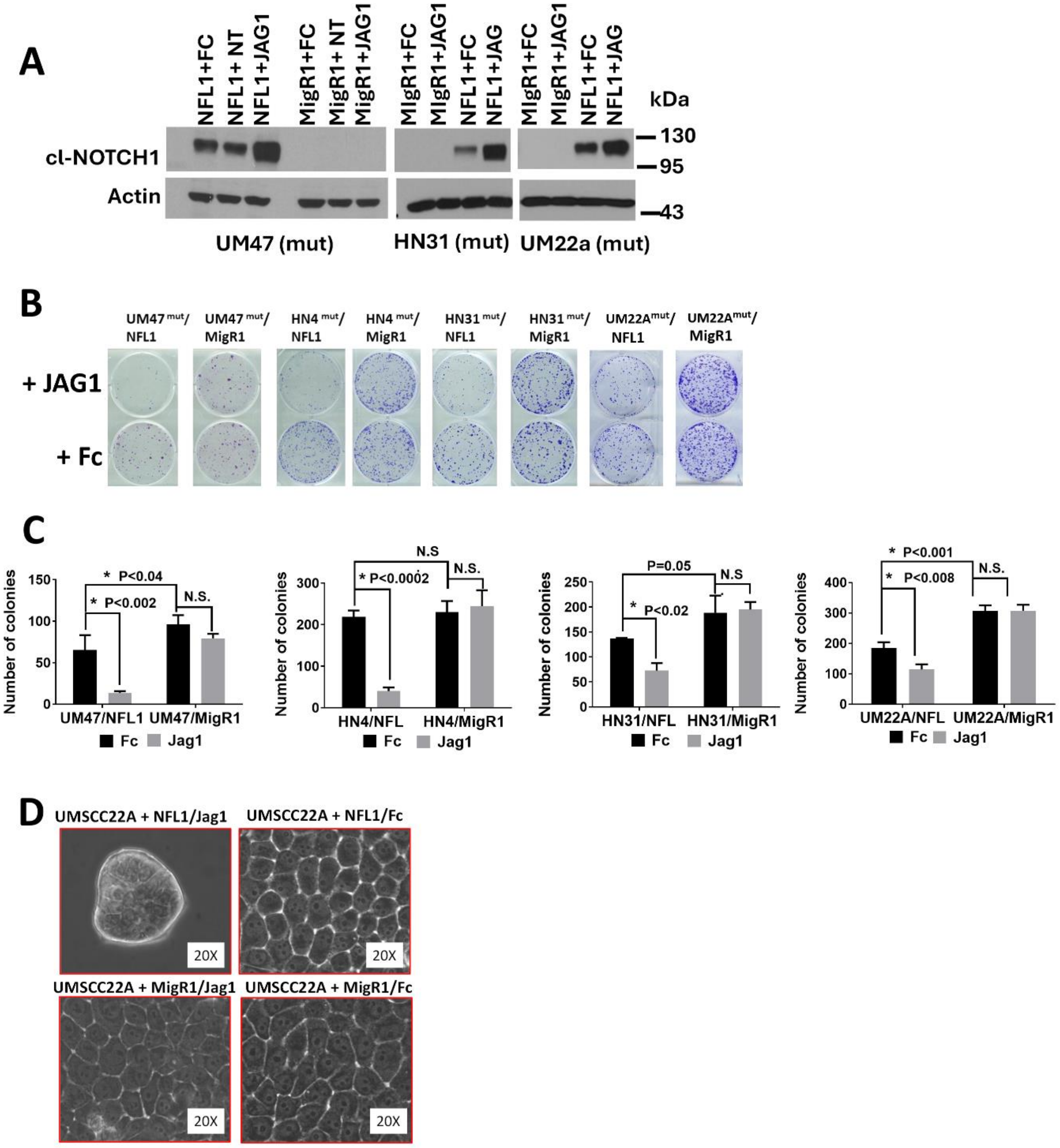

Supplementary Figure 1. **Restoration of NOTCH1 signaling inhibits *in vitro* growth and alters morphology in *NOTCH1* mutant HNSCC tumors. A**. Ectopic expression of WT full-length NOTCH1 receptors (NFL1) leads to activation and cleavage of NOTCH1 (cl-NOTCH1) protein in 3 different NOTCH1-mutant HNSCC cell lines that increases upon 16 h stimulation with immobilized JAG1 ligand, compared to control FC protein. **B**. Continued growth on immobilized JAG1 for 8-10 days inhibits colony formation in *NOTCH1*-mutant cell lines with restored NFL1. **C**. Quantitation of NFL1-mediated growth inhibition of colonies. **D.** Restoration of NFL1 signaling in UMSCC-22A led to dramatic reduction in cell size and formation of loosely attached tumor spheroids after 5 days only in the presence of JAG1. UM47 = UMSCC47, UM22A = UMSCC22A. Differences in colony number between treatments for each cell line were analyzed by an ANOVA, with P-values for individual comparisons determined with a post-hoc Tukey test.

Supplementary Figure 2

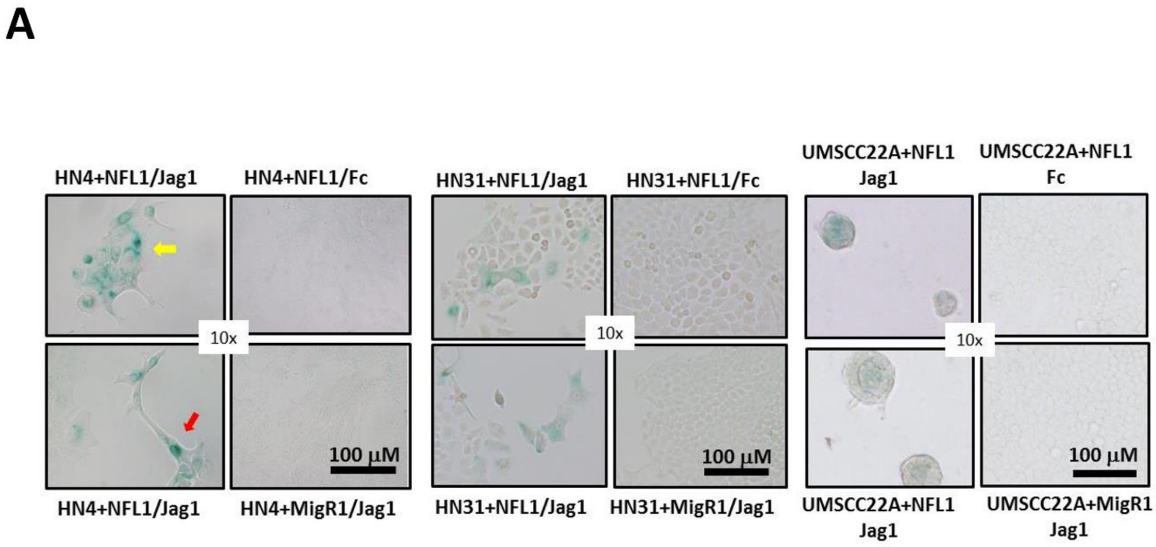

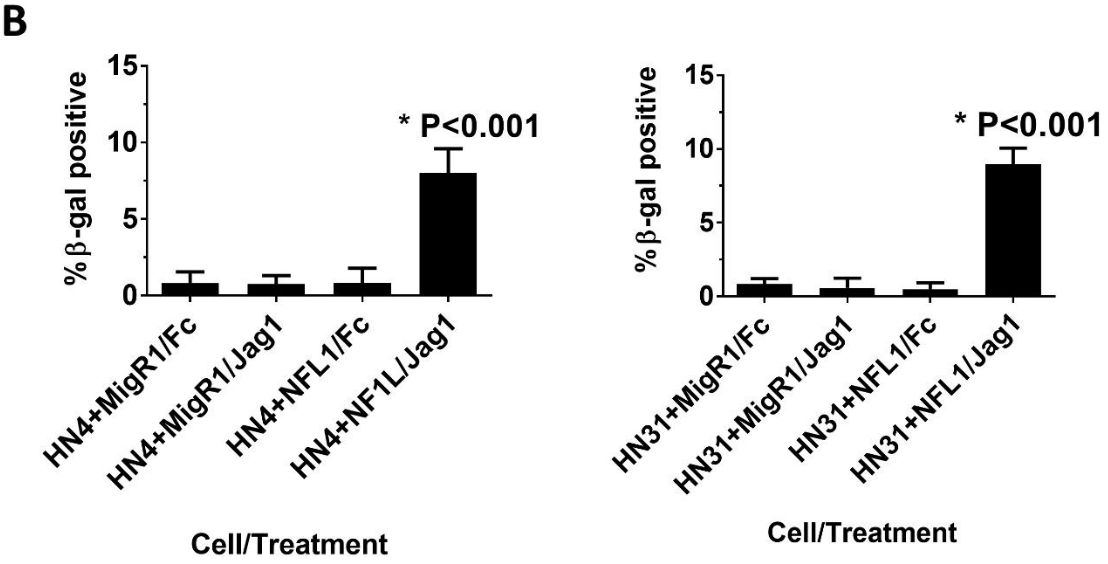

Supplementary Figure 2. **Restoration of NOTCH1 signaling induces senescence in NOTCH1 mutant tumor cell lines**. **A.** Photomicrographs of *NOTCH1*-mutant HNSCC cell lines infected with NFL1 or empty MigR1 grown on either JAG1 or FC control protein and stained for β-gal after 5 to 7 days culture. **B.** Quantitation shows significant elevation of β-gal staining in the presence of NFL1 expression and growth on JAG1 ligand. Differences in percentage of β-gal staining among treatments for each cell line were analyzed by an ANOVA, with P-values for individual comparisons determined using a post-hoc Tukey test. Treatments with no P-value showing were not significantly different from each other but were all significantly different from cells infected with NFL and plated on JAG1 ligand.

Supplementary Figure 3

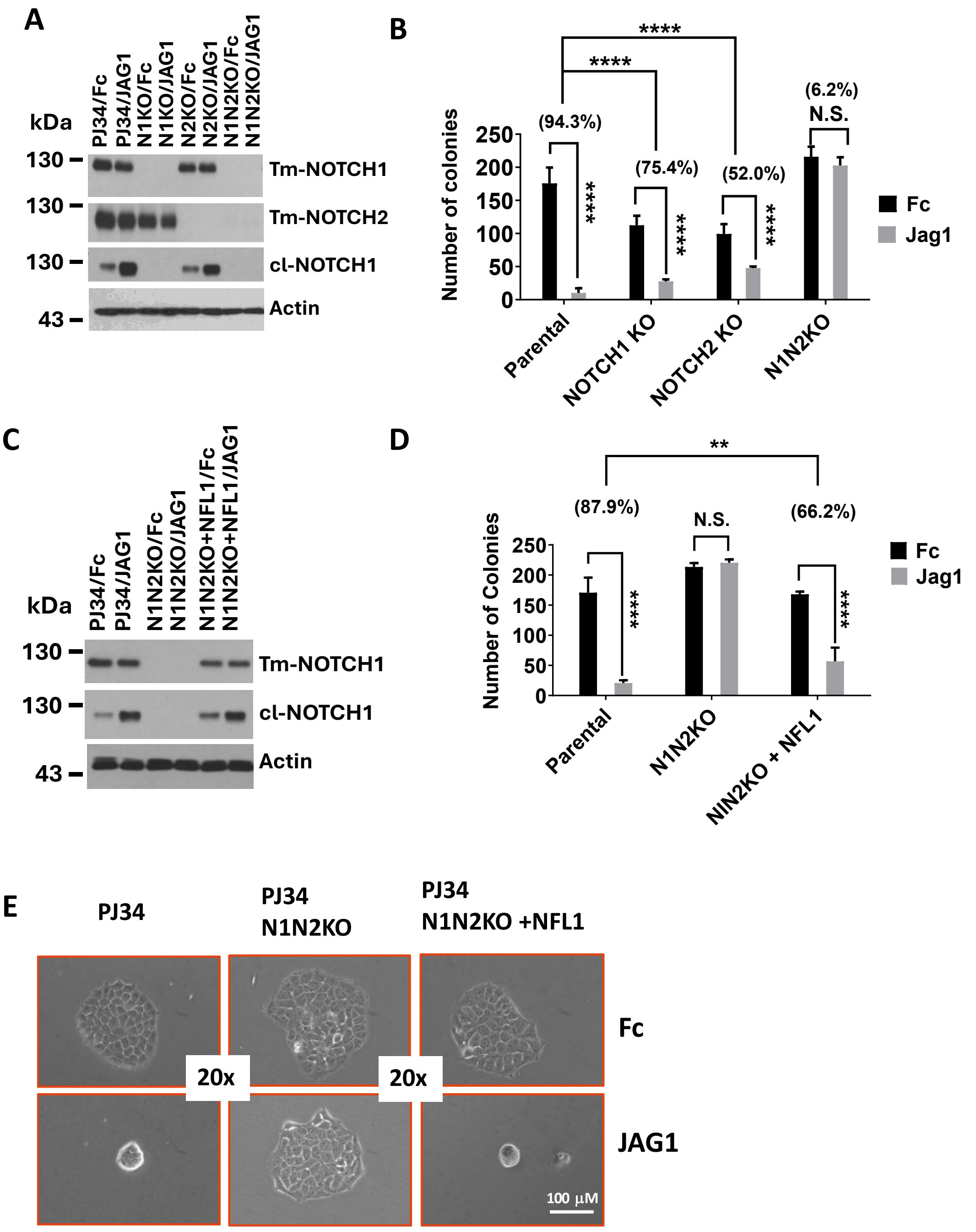

Supplementary Figure 3. **Both NOTCH1 and NOTCH2 signaling contribute to JAG1-induced growth inhibition but NOTCH1 signaling is sufficient. A.** CRISPR KO of either *NOTCH1* (NIKO), NOTCH2 (N2KO) or double KO (N1N2 KO) in PJ34 were validated by western blotting for total NOTCH1 and NOTCH2. Appearance of cl-NOTCH1 was absent in N1KO or N1N2KO after stimulation with JAG1 for 16 h. **B.** N1KO or N2KO partially blocked JAG1-induced inhibition of colony formation compared to that observed in parental cells ($P < 0.0001$) , while double N1K2 KO completely prevented growth inhibition. **C.** Infection with ectopic NFL1 cDNA restored NOTCH1 signaling in N1N2 KO cells. **D.** Re-Expression of NFL1 in PJ34 after N1N2KO partially restored JAG1-mediated inhibition of colony growth. **E.** Loss of both *NOTCH1* and *NOTCH2* (N1N2KO*)* in PJ34 protected cells from JAG1-induced morphology changes while re-expression of NFL1 restored spheroid formation caused by growth on JAG1. Changes in colony growth after growth on JAG1 ligand for each cell line compared to growth on FC control protein, or the differences in JAG1-induced effects between cell lines were analyzed with a cell means model using simple and interaction contrasts. ****$P < 0.0001$, **$P < 0.01$.

Supplementary Figure 4

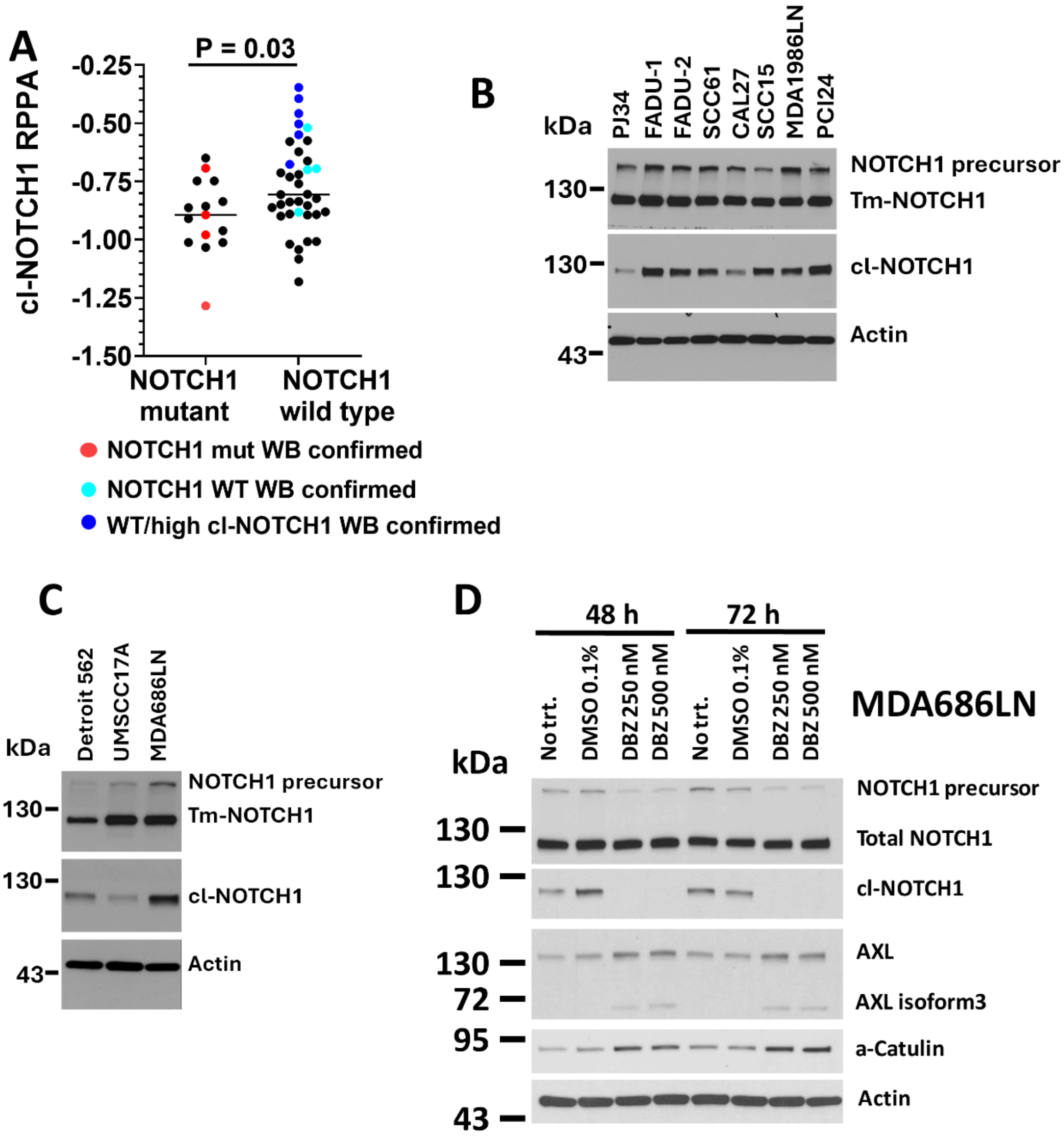

Supplementary Figure 4. RPPA identifies *NOTCH1* WT cell lines with elevated baseline cl-NOTCH1expression which was inversely correlated with protein expression of AXL and a-CATULIN. **A.** Normalized RPPA values of cl-NOTCH1 protein measured from *NOTCH1* mutant and *NOTCH1* WT HNSCC cell lines. Cell lines where cl-NOTCH1 protein was validated by western blots (WB) are indicated by colored symbols, with red dots for NOTCH1 mutants (HN31, UMSCC22A, UMSCC47, and HN4), light blue dots for NOTCH1 WT cells with low to moderate baseline cl-NOTCH (PJ34, 183, CAL27, and UMSCC1), and dark blue dots corresponding to *NOTCH1* WT cells with high basal cl-NOTCH1 (FaDu, PCI24, SCC61, SCC15, and MDA1986LN). **B.** Western blot confirmation of high cl-NOTCH1 protein in subsets of WT cell lines, compared to PJ34 with low baseline activation. C. Confirmation of high baseline NOTCH1 activation in MDA686LN. **D**. DBZ inhibited cl-NOTCH1 protein and increased levels of both AXL and α-CATULIN protein. Differences in average cl-NOTCH1 measured by RPPA between *NOTCH1* mutant and wild type cell lines were analyzed with a two-sided student's t-test (A).

Supplementary Figure 5

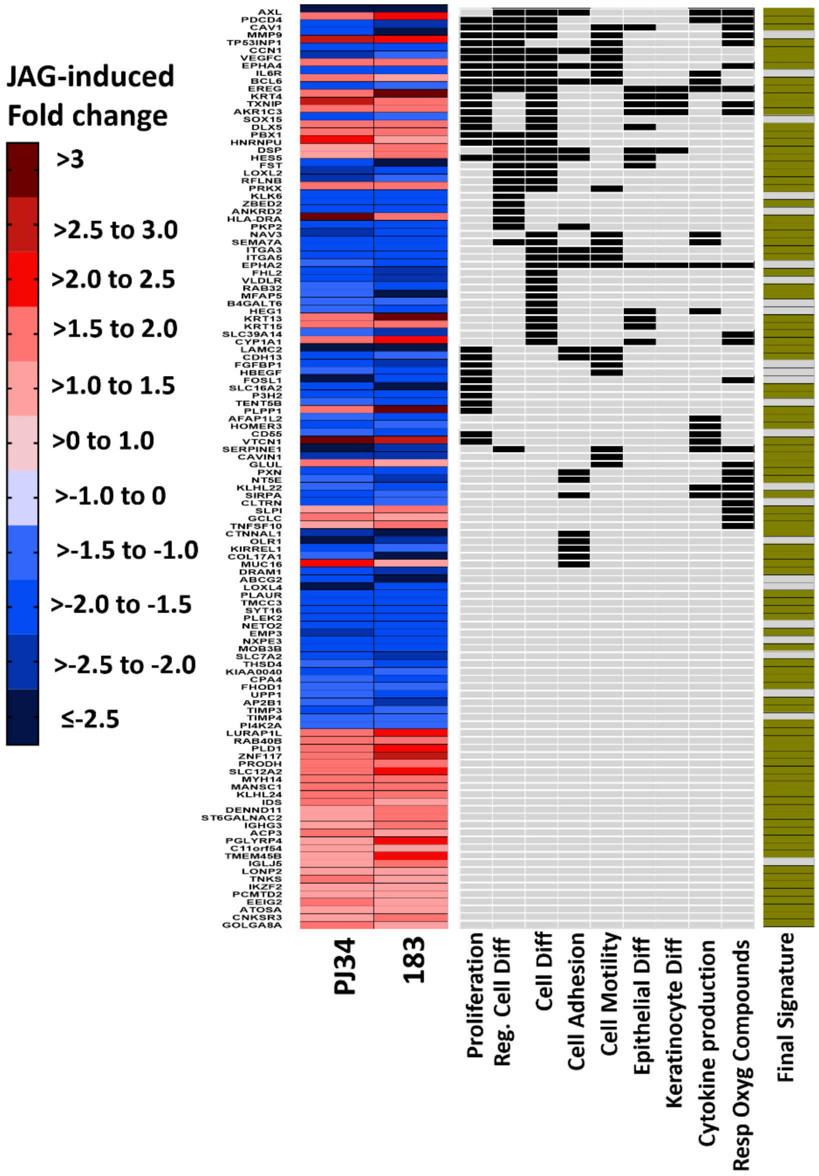

Supplementary Figure 5. NOTCH1 regulates genes involved in proliferation, differentiation, attachment, motility, and response to oxygen containing compounds. The top 120 significant genes (i.e., minimum of 1.4-fold change, **FDR <0.1**) from *NOTCH1* WT PJ34 and 183 cells regulated in common by growth on JAG1 and their biological pathways. The fold change in each cell line after growth for 5 days on JAG1 is annotated vertically as a color gradient with red boxes representing increases, and blue boxes representing decreases. Membership in select GO pathways that were enriched in the gene set is annotated with black boxes, and gold boxes indicate genes that were included in the final NOTCH1 gene activation signature discussed in the text.

Supplementary Figure 6

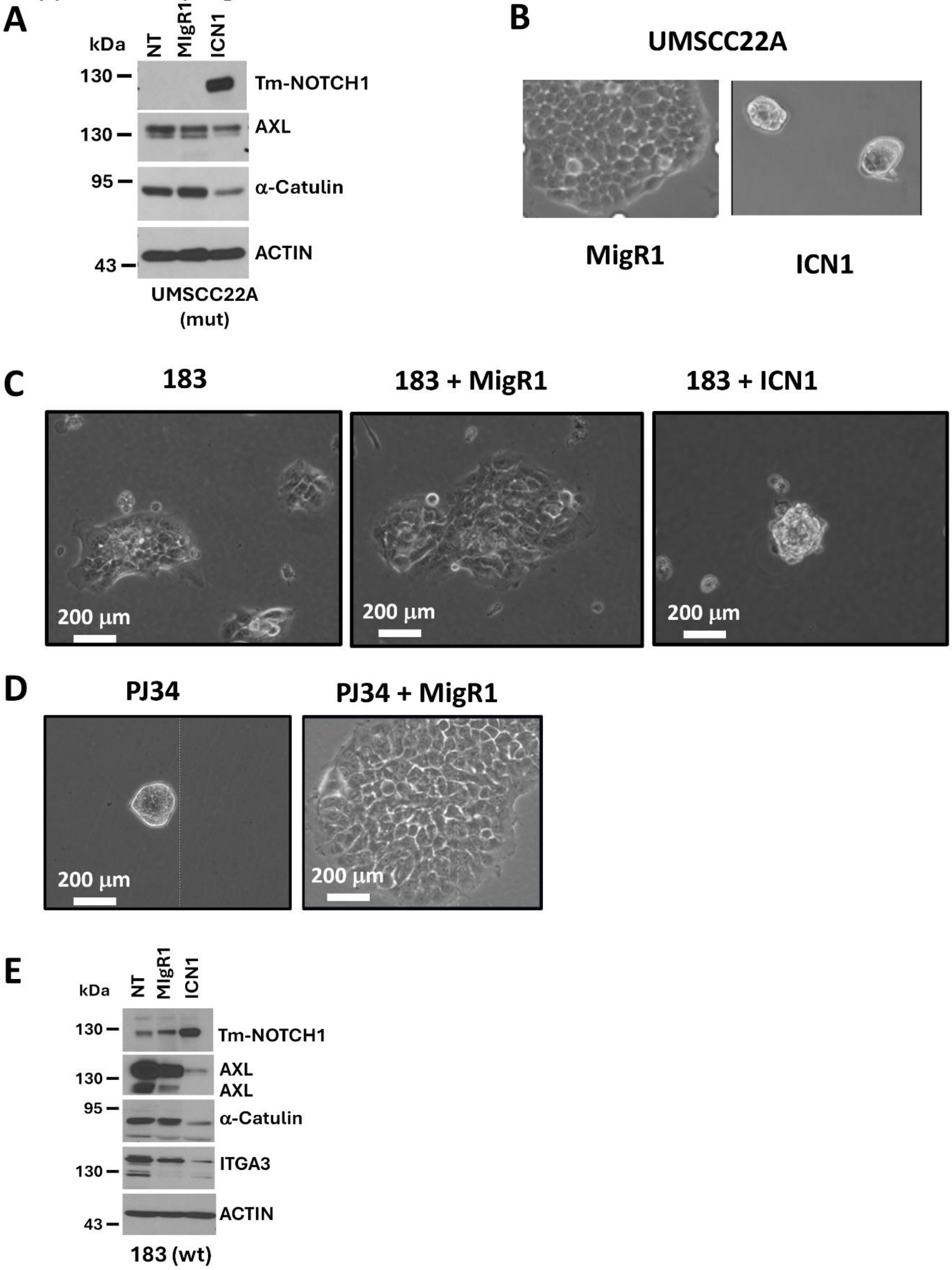

Supplementary Figure 6. **Ectopic expression of ICN1 mimics changes induced by JAG1 stimulation**. **A.** Infection with ICN1 but not empty vector (MigR1) strongly inhibited protein expression of AXL and α-CATULIN. Widely used ICN1 cDNA product which begins several amino acids downstream of the native cleavage site is recognized by antibodies to the C-terminal region of NOTCH1 but not antibodies specific to the cleavage site. **B.** Expression of ICN1 in *NOTCH1* mutant UMSCC22A produced the same morphological changes observed earlier upon expression of NFL1 and growth on JAG1. **C.** Expression of ICN1 in *NOTCH1* WT 183 produced the same morphological changes observed earlier with growth on JAG1. **D.** Expression of ICN1 but not MigR1 control in *NOTCH1* WT PJ34 produced the same morphological changes observed earlier with growth on JAG1. E. ICN1 expression triggered decreased protein expression of AXL, α-CATULIN, and ITGA3 in 183 cells.

Supplementary Figure 7

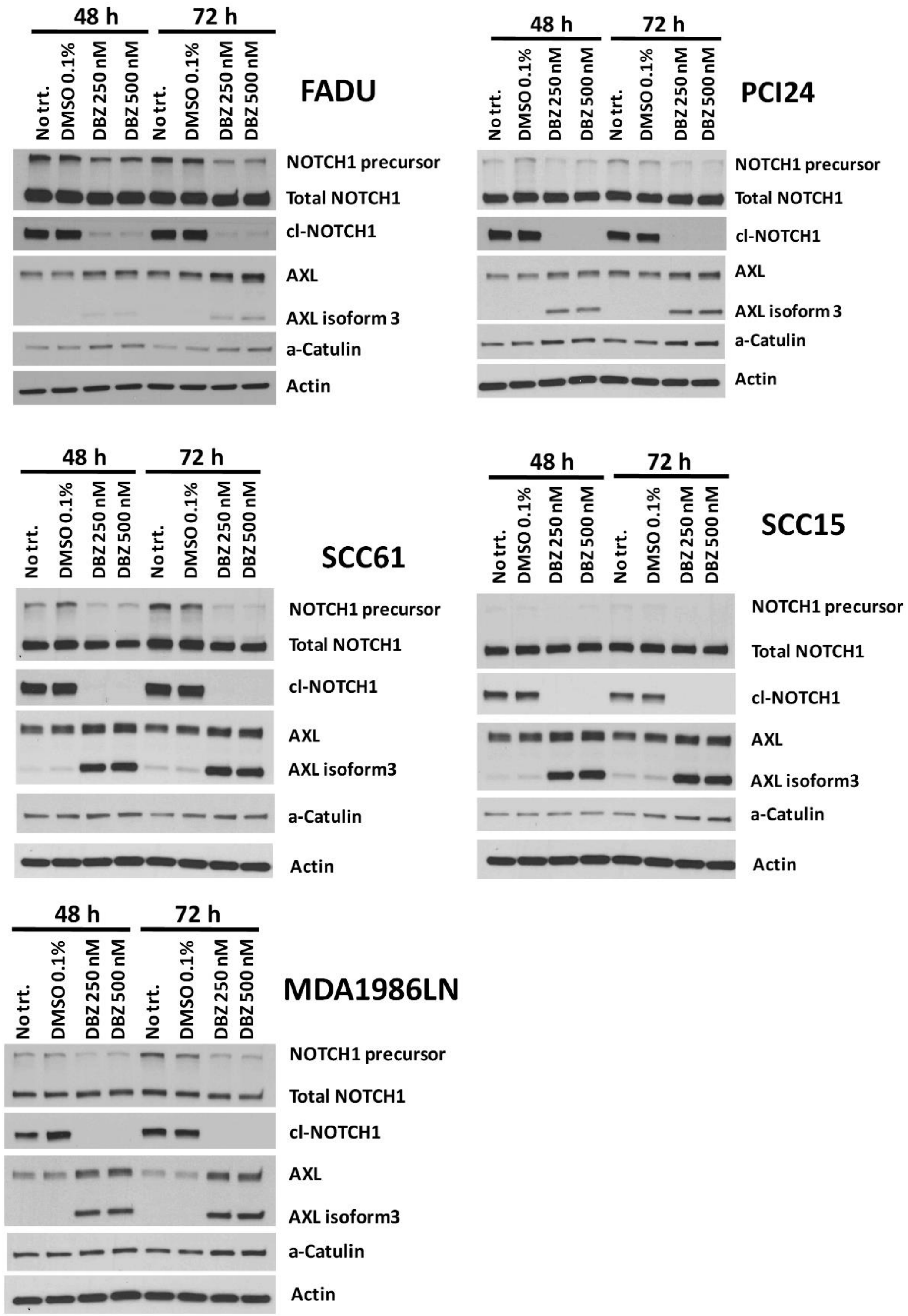

Supplementary Figure 7.**Evidence of NOTCH1 signaling that suppresses AXL and α-CATULIN expression in a subset of NOTCH1 wt HNSCC tumor cell lines.** Protein levels of both AXL and α-CATULIN increase when activated cl-NOTCH1 formation is blocked with the NOTCH inhibitor DBZ in five different *NOTCH1* WT cell lines with high endogenous NOTCH1 activity.

Supplementary Figure 8

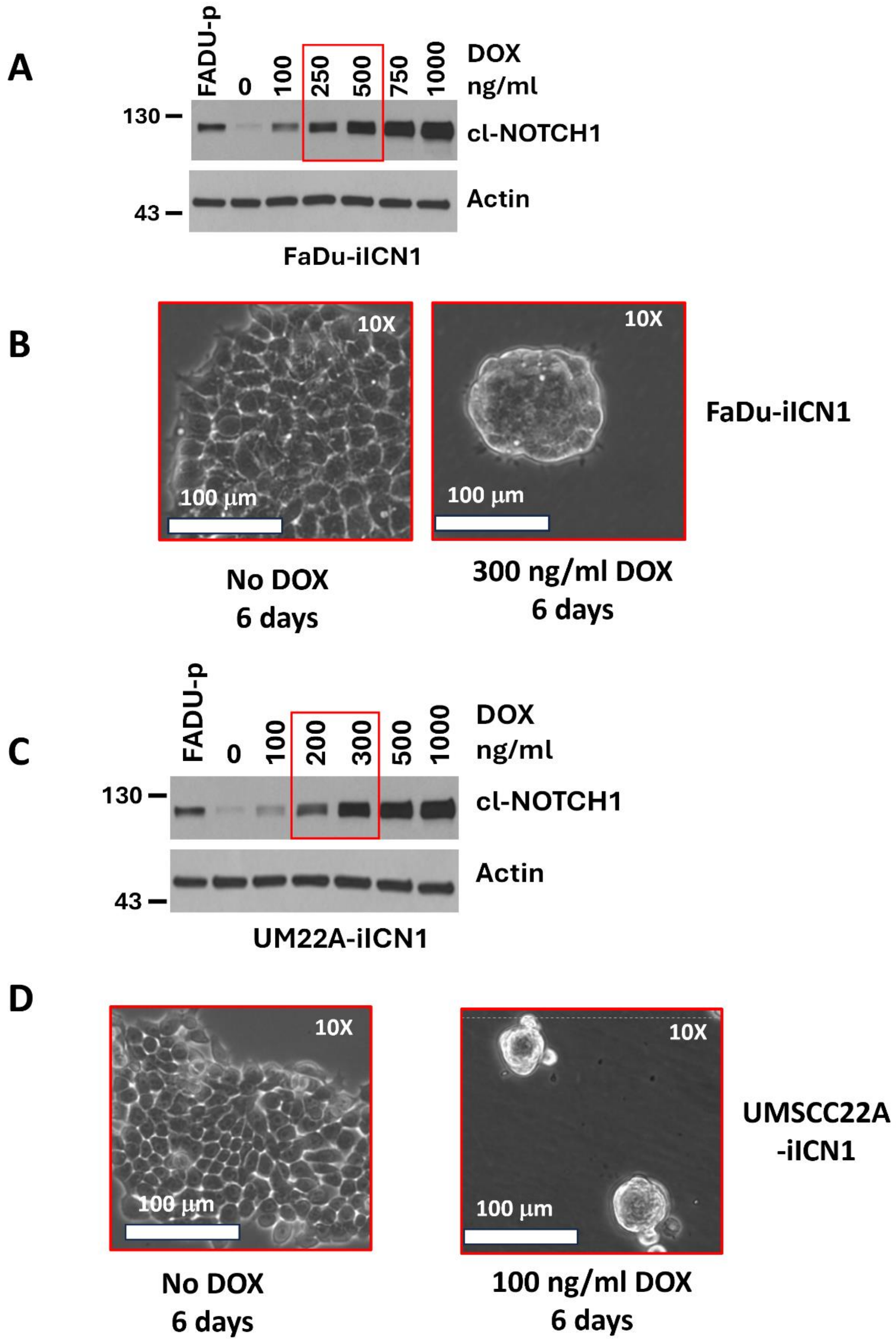

Supplementary Figure 8. Physiological levels of iICN1 trigger morphology changes in *NOTCH1* WT and mutant tumor cell lines. A. Titration demonstrates that treating FADU NIKO cells engineered to express iICN1 (FaDu-iICN1) with 250ng-500 ng/ml DOX for 36 h induces protein levels of iICN1 equivalent to parental FaDu (FADU-p) **B**. As little as 300 ng/ml DOX for 6 days causes tumor spheroid formation in FaDu-iICN1. **C.** cl-NOTCH1 protein levels in UMSCC22A-iICN1 treated with 200-300 ng/ml DOX for 36 h are similar to basal levels in parental FaDu. **D.** Tumor spheroid formation in UMSCC22A-iICN1 cells after 6 days treatment with 100 ng/ml DOX.

Supplementary Figure 9

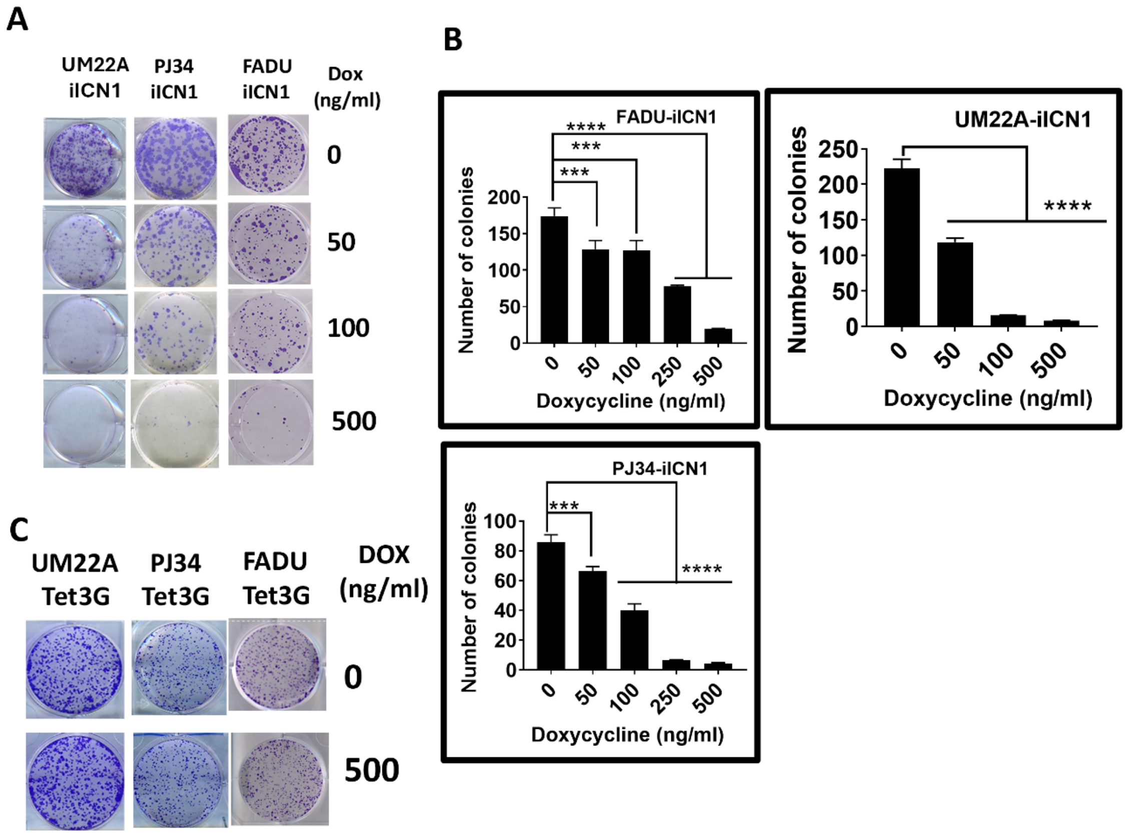

Supplementary Figure 9. Dose response inhibition of colony formation by iICN1 in NOTCH1 mutant and WT cell lines. A. Crystal violet stain of colonies after increasing doses of DOX. **B**. Quantitation of colony inhibition after treating cells with increasing DOX doses. **C.** Treatment of control Tet3G expressing cell lines, lacking an iICN1 construct, with 1000 μg/ml DOX did not inhibit colony growth. Differences in colony number between treatments for each cell line were analyzed with an ANOVA followed by a post-hoc Tukey test to determine P-values for specific comparisons. *** $P < 0.001$, **** $P < 0.0001$.

Supplementary Figure 10

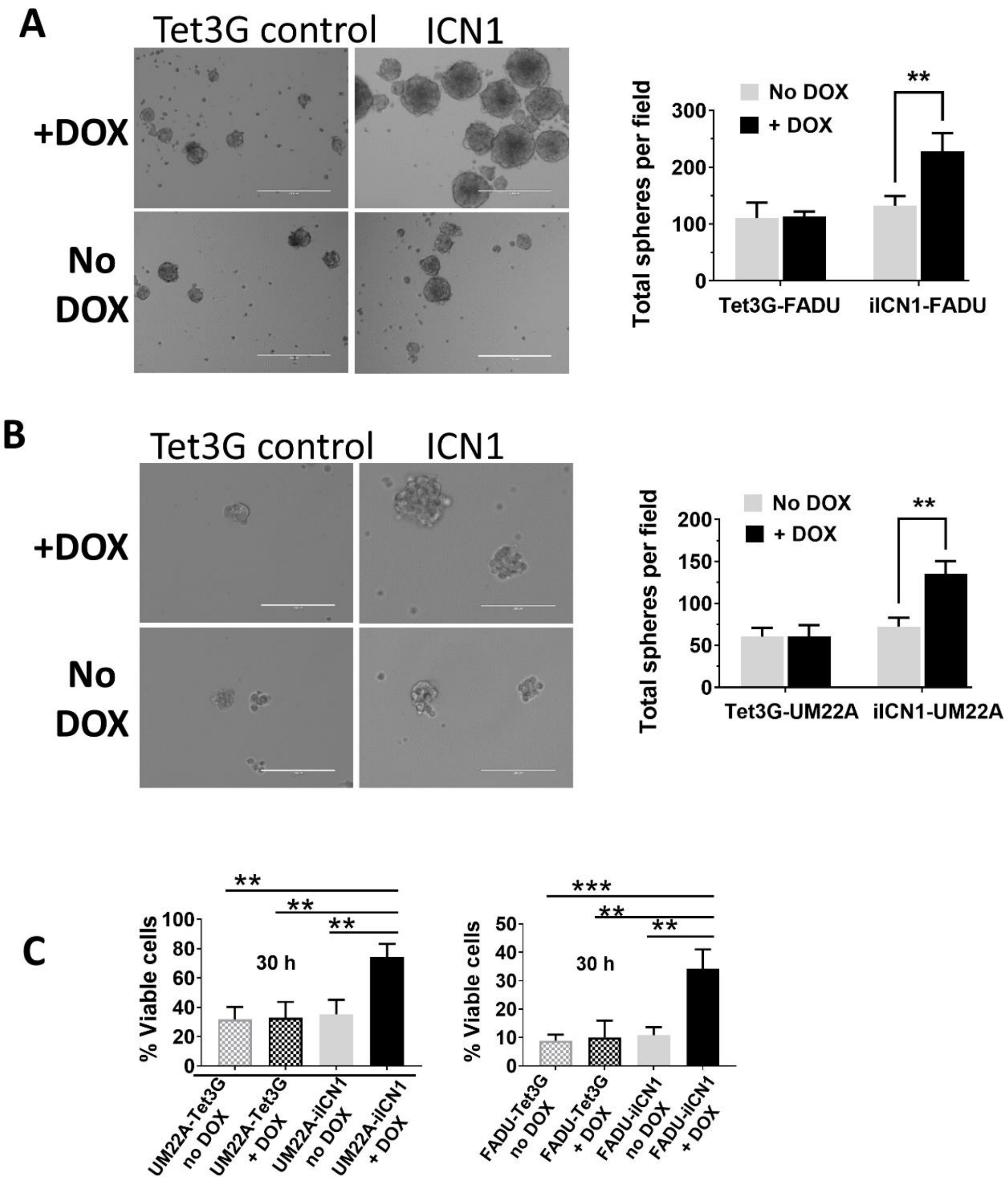

Supplementary Figure 10. NOTCH1 activation increases tumor spheroid growth and anoikis resistance. A. Addition of 1000 mg/ml DOX increases the number of orospheres derived from FaDu-iICN1 in non-adherent low serum concentrations for 1 week. **B**. Increased orosphere numbers after growing UMSCC22A-iICN1 in 300 mg/ml DOX non-adherent low serum cultures for 1 week. **C.** DOX was added to attached UMSCC22A-iCN1 at 300 mg/ml, FaDU-iICN1 at 1000 mg/ml, or control Tet3G cells for 48 h before single cell suspensions were prepared in low serum containing media and rotated in non-adherent tubes inside a humidified 37 ^{0}C cell incubator for an additional 30 hours, in continued presence or absence of DOX. Viability of replicate cultures was examined with an automated cell counter. Differences in spheroid count in cell lines before and after DOX treatment (A and B) were determined with a two-sided student's t-test, and treatments lacking P-values were not significantly different from each other (P > 0.05). Differences in the percentage of viable cells expressing the control plasmid Tet3G in the presence or absence of DOX or in iICN1 infected cells minus DOX were all compared to iICN1 infected cells plus Dox with an ANOVA followed by a Dunnett's multi-comparison test (C). ** P <0.01, *** P < 0.001.

Supplementary Figure 11

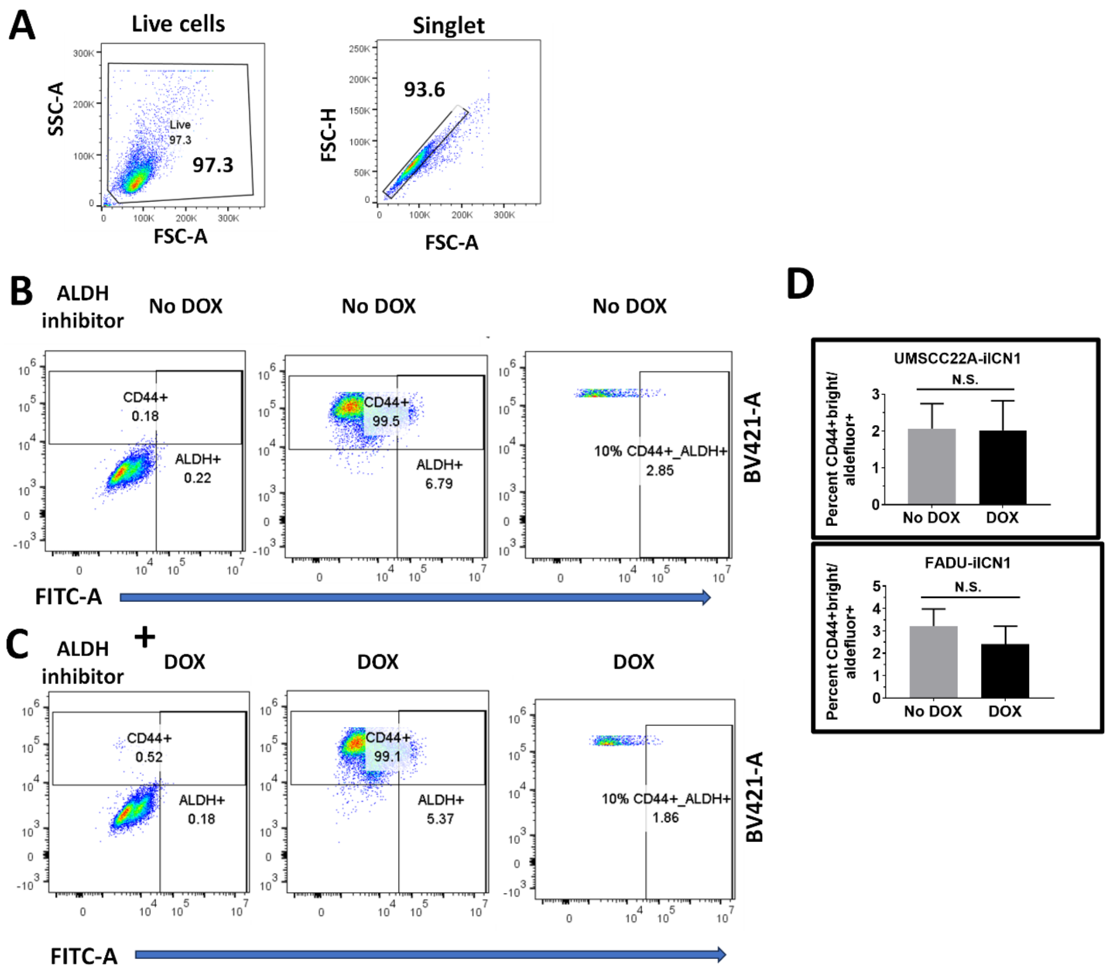

Supplementary Figure 11. **Activation of NOTCH1 does not increase the percentage of CD44+bright ALDEFLUOR-positive cells in NOTCH1 mutant or WT tumor lines. A.** Flow cytometry gating strategy to identify live single cells. **B.** Representative gating of UMSCC22A-iICN1 cells in absence of Dox. The sample is split and one tube treated with a BV-421 conjugated isotype control antibody and the aldehyde dehydrogenase inhibitor (DEAB) before adding fluorescein conjugated substrate to set the background gates (left panel). The second tube is incubated with BV421-A conjugated anti-CD44 and Aldefluor substrate without *DEAB* to gate on the CD44+/Aldefulor+ population (middle panel) and a single fixed threshold is set for the top 10% brightest CD44+ (e.g., CD44+ bright), which is uniformly applied to all remaining UMSCC22A-iICN1 samples. The CD44+ bright population is further analyzed for the percentage of Aldefluor+ cells (right panel). **C.** A UMSCC22A-iICN1 sample pretreated with 200 ng/ml DOX for 48 h to induce ICN1 before staining is gated in the same fashion, except Aldefluor gating is set with a sample specific tube treated with DEAB (left panel) and CD44+bright cells are analyzed in a companion tube without DEAB for percentage of Aldefulor+ using the previously set CD44+ threshold (right panel). D. The total percentage of CD44+bright/Aldefluor+ cells does not increase after iICN1 induction in either UMSCC22A-iICN1 or FaDu-iICN1. No significant differences were found in the percentage of CD44+bright/Aldefluor+ cells following a two-sided student's t-test.

Supplementary Figure 12

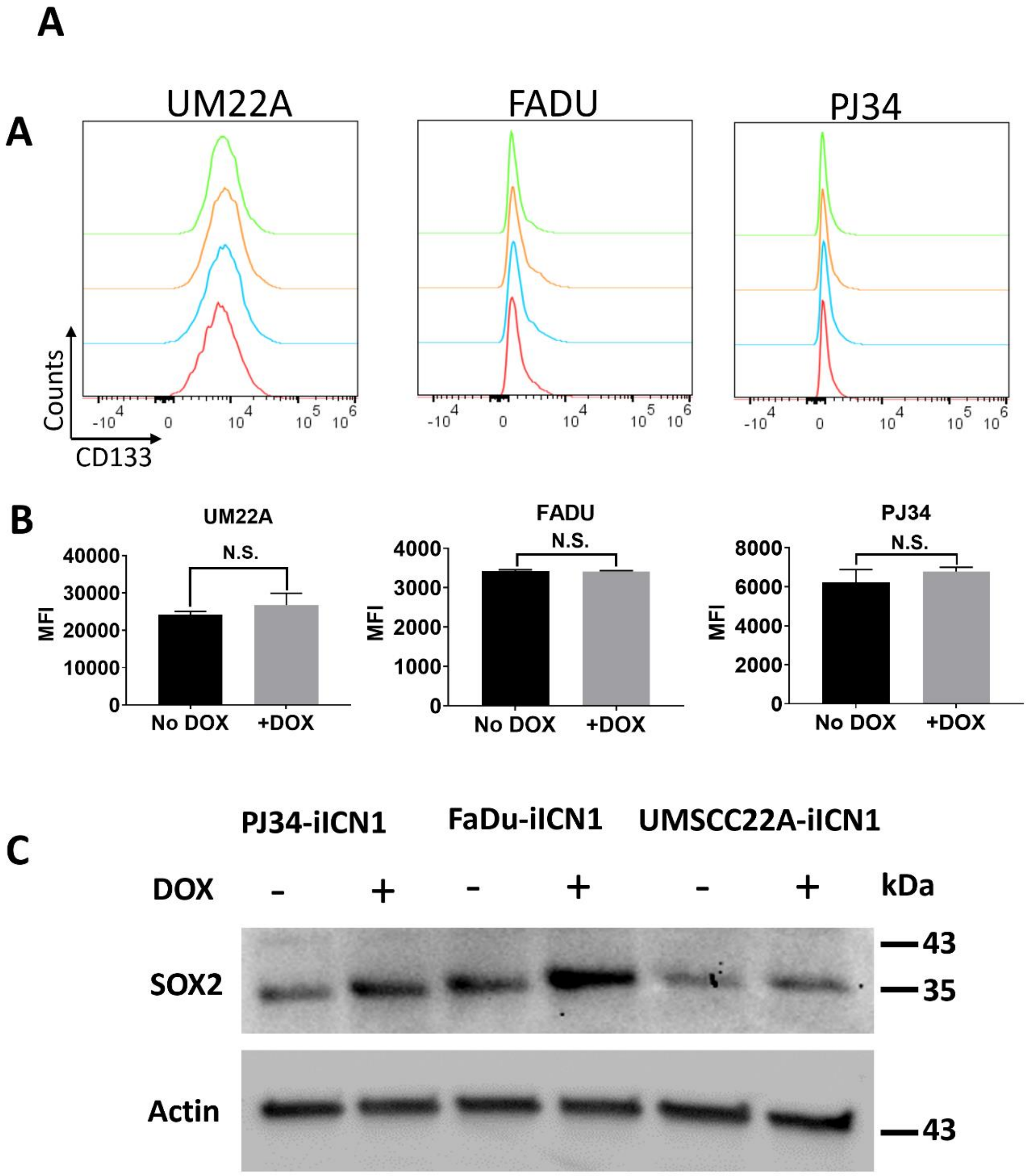

Supplementary Figure 12. Activation of NOTCH1 fails to increase surface CD133+ but increases expression of SOX2 in *NOTCH1* mutant (UMSCC22A-iICN1), or *NOTCH1* WT cell lines (FaDu-iICN1 and PJ34-iICN1). A. Cells were incubated for 48 h with or without DOX at 200 ng/ml (UMSCC22A), 300 ng/ml (FaDu), or 1000 ng/ml (PJ34) before staining with antibody to surface CD133 by flow cytometry. Fluorescent intensity histograms are shown for control biological replicates without DOX (red and light blue traces) or after DOX treatment (green and orange traces). **B.** Statistical comparison of CD133 mean fluorescence intensity (MFI). No significant differences were found in the mean intensities following a two-sided student's t-test. **C.** Western blot analysis of SOX2 protein expression in similarly treated cells.

Supplementary Figure 13

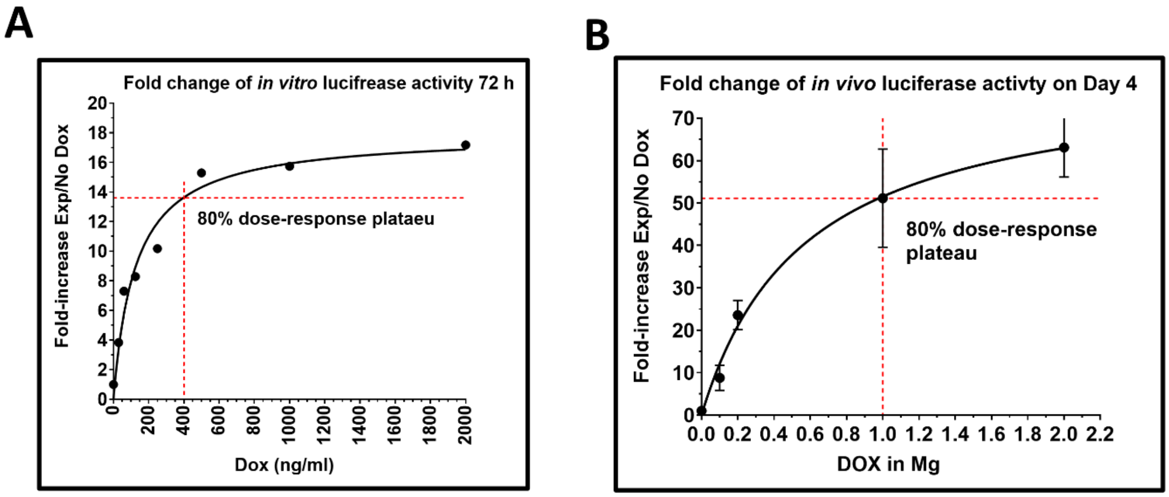

Supplementary Figure 13. ***In vitro* and *in vivo* dose response of the inducible DOX promoter**. **A.** UMSCC22A Tet3G reporter cells stably expressing firefly luciferase cloned into pLVX-TRE3G-mCherry were seeded into 6 well plates and luciferase activity measure in a plate reader 36 hours after induction with increasing concentrations of DOX. A dose of ~400 ng/ml resulted in luciferase activity that was 80% of the *dose response* plateau, but not outside linear response of the plate reader **B**. Standard curve relating relative *in vivo* luciferase luminescence to DOX concentration given to mice by oral gavage for 4 days, demonstrating that 1 mg DOX yielded activity that was 80% of the in *vivo* plateau.

Supplementary Figure 14

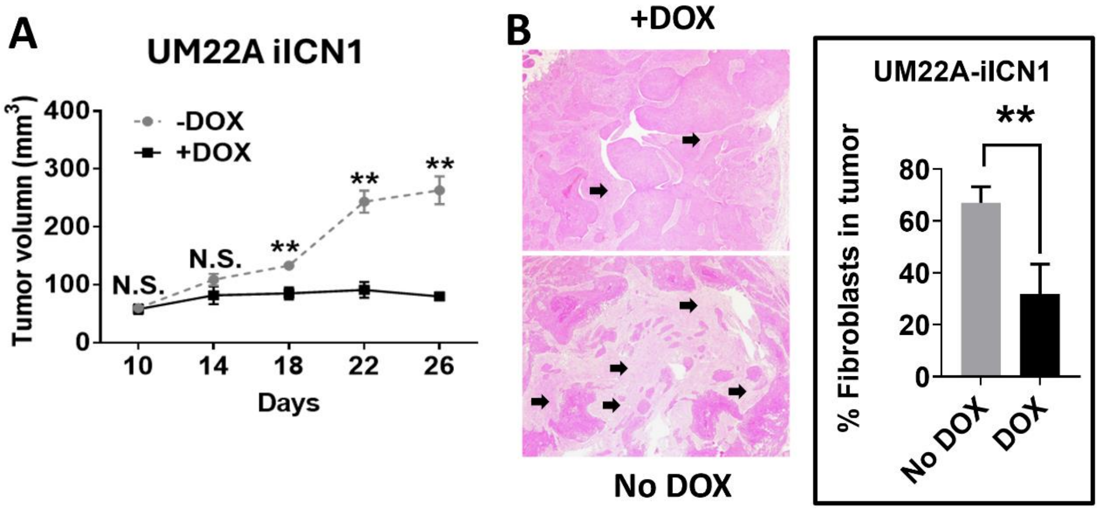

Supplementary Figure 14. **Activation of NOTCH1 signaling in *NOTCH1* mutant tumors profoundly inhibits *vivo* tumor growth and reduces the number of CAF cells. A.** UMSCC22A-iICN1 was inoculated into mouse flanks and mice were randomly assigned to receive either no DOX or daily DOX (1 mg) by oral gavage for two weeks. Tumor volumes were compared over time. **B**. H&E staining of a representative tumor that eventually formed in mice treated with DOX (top panel) and a tumor that formed in the absence of DOX (bottom panel). Arrows designate areas predominately populated by CAFs. The percentage of CAFS in tumors was significantly lower when ICN1 was induced with DOX. Differences in tumor volumes between treatment groups at each time point were evaluated by a two-sided student's t-test. ** $P < 0.01$.

Supplementary Figure 15

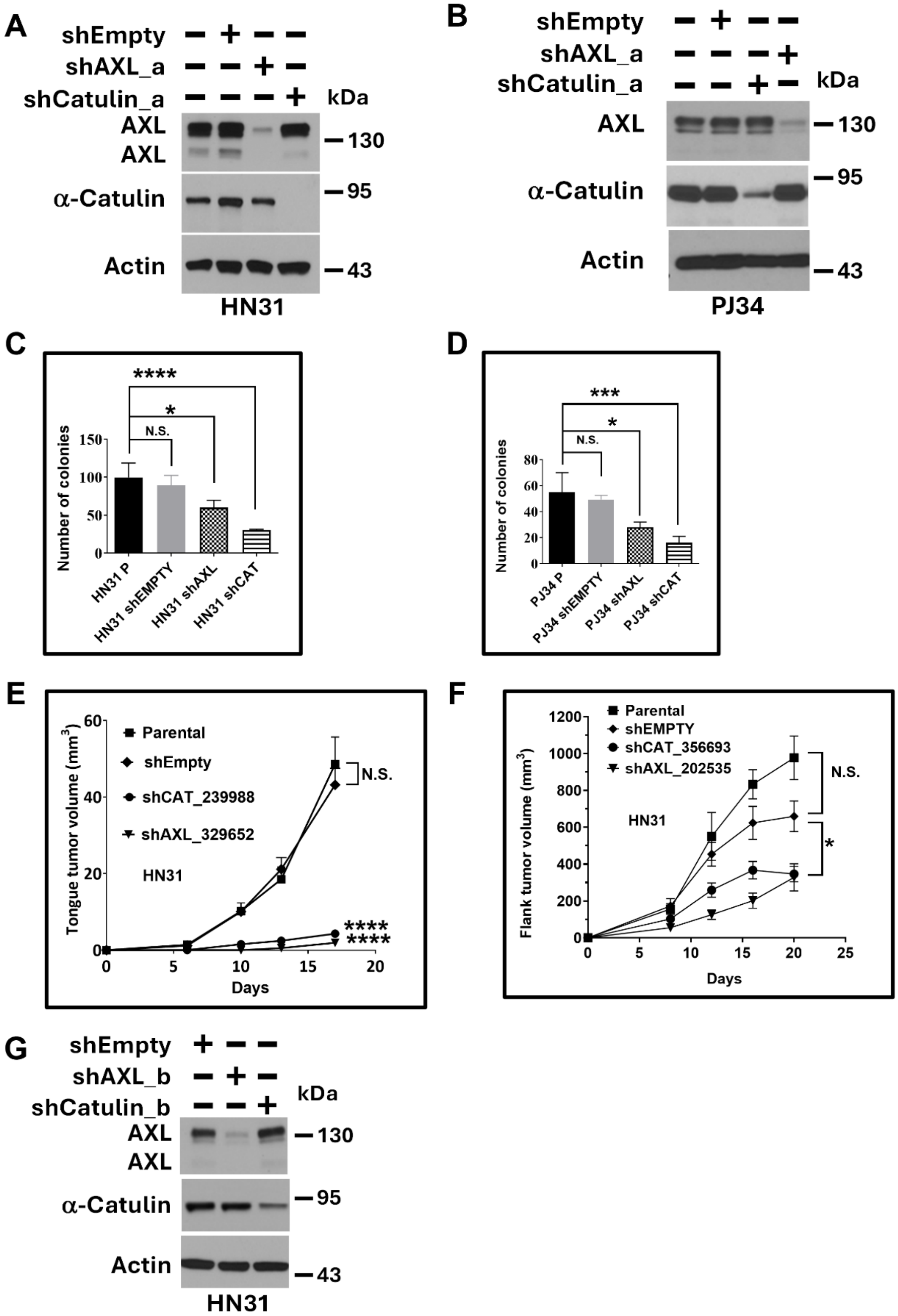

Supplementary Figure 15. Knockdown of *AXL* and α-CATULIN to mirror NOTCH1 signaling inhibits *in vitro* and *in vivo* tumor growth. A. Western blot confirmation of AXL and α-CATULIN protein inhibition following KD in *NOTCH1* mutant HN31 cells 96 h post-infection with shAXL_a (Cat # 329652) and shCatulin_a (Cat # 239988). **B.** Western blot confirmation of AXL and a-CATULIN protein inhibition following shRNA KD in NOTCH1 WT PJ34 cells 96 h post-infection with specific shRNA. **C.** KD of AXL (i.e., shAXL_a) and a-CATULIN (i.e., shCatulin_a) blocks *in vitro* colony formation in HN31. **D.** KD of AXL (shAXL_a) and a-CATULIN (shCatulin_a) blocks *in vitro* colony formation in PJ34. **E.** KD of AXL and a-CATULLIN profoundly inhibits HN31 *in vivo* tumor growth in an orthotopic tongue tumor model. **F.** KD of AXL and a-CATULLIN with alternate shRNA vectors targeting different regions inhibits HN31 *in vivo* tumor growth in a subcutaneous flank model. **G.** Western blot validation of target KD with alternate shCAT_356693 (shCatulin_b) and shAXL_202535 (shAXL_b) vectors used in the subcutaneous tumor experiment. Difference in colony formation or *in vivo* tumor volumes between treatments were analyzed by an ANOVA, followed by a post-hoc Tukey test to determine P-values for individual comparisons (C, D, E, F). $^{*}P < 0.05$, $^{***}P < 0.001$, $^{****}P < 0.0001$

Supplementary Figure 16

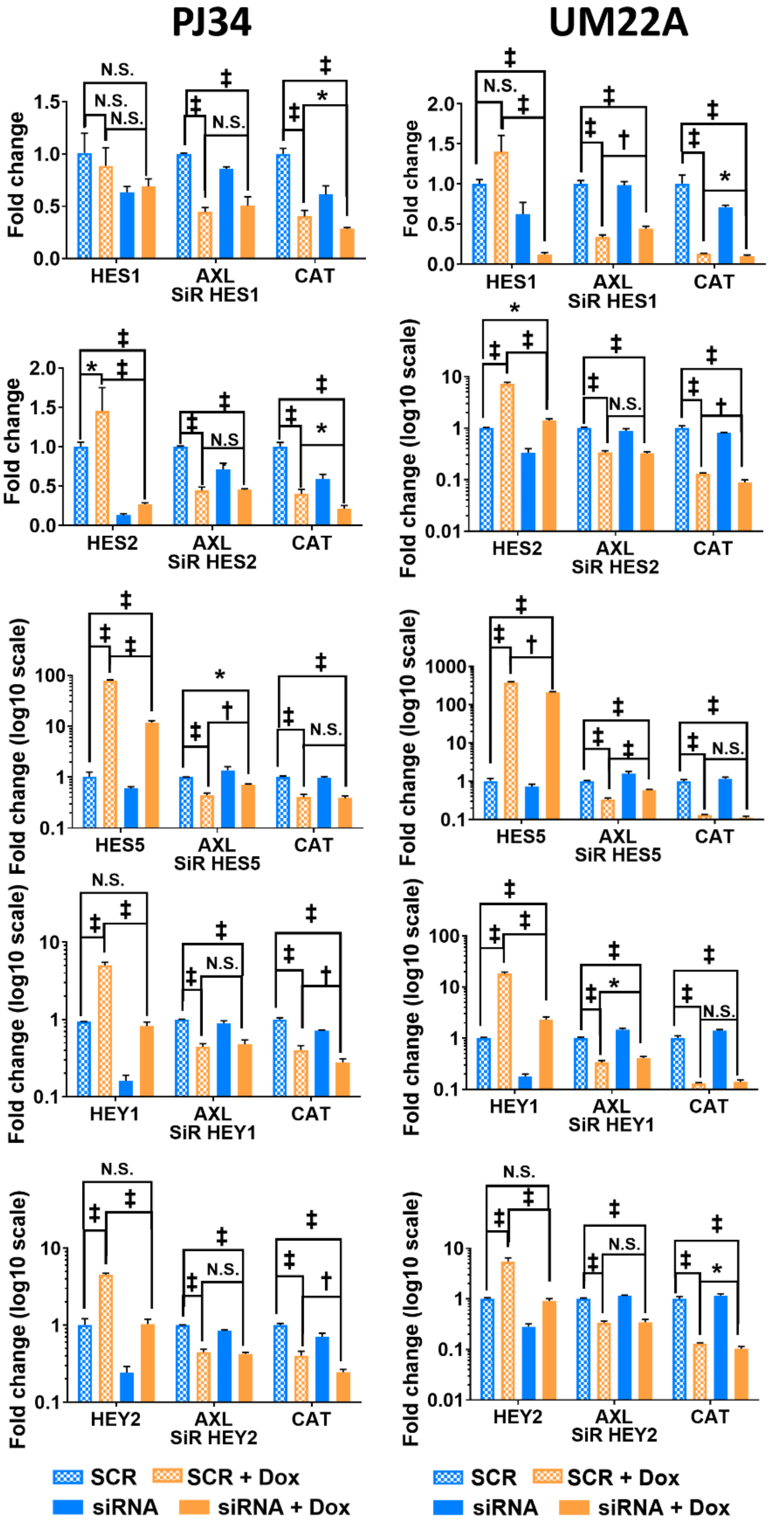

Supplementary Figure 16. Canonical HES/HEY family members are not key mediators of ICN1 induced reductions in *AXL* and a-*CATULIN* expression. PJ34-iICN1 (left) or UMSCC22A-iICN1 (right) were pretreated with siRNA (SIR) to HES1, HES2, HES5, HEY1, or HEY2 or control non-targeting siRNA (SCR) for 48 h before replating in the absence or presence of 300 ng/ml DOX for 24 h. Expression of the HES/HEY knockdown targets, AXL, and α-Catulin were quantitated by qPCR. Differences in RNA among treatments for each gene were evaluated with an ANOVA followed by a post-hoc Tukey test to determine P-values for individual comparisons. * P <0.05, † P < 0.001, ‡ P <0.0001.

Supplementary Figure 17

A

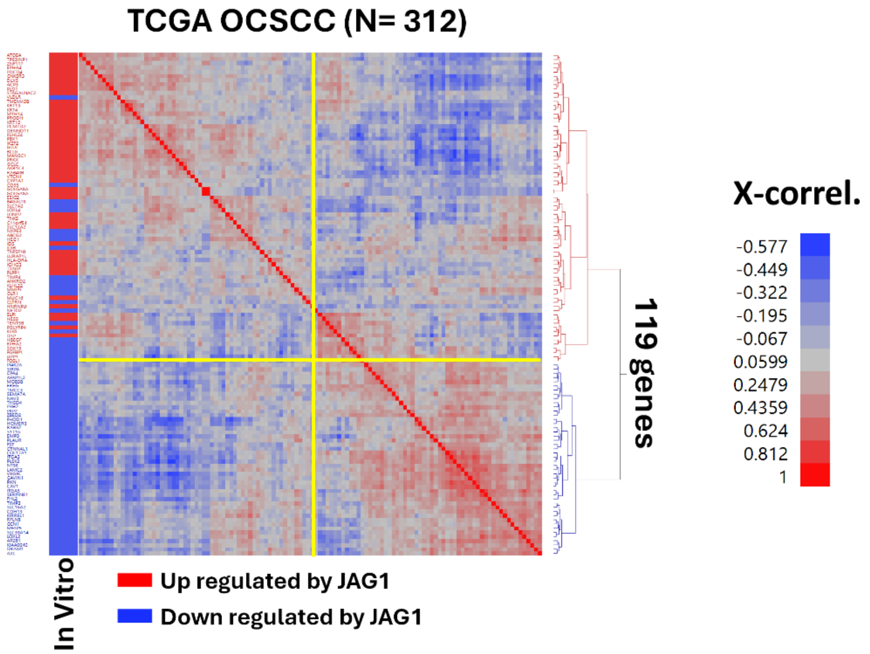

B

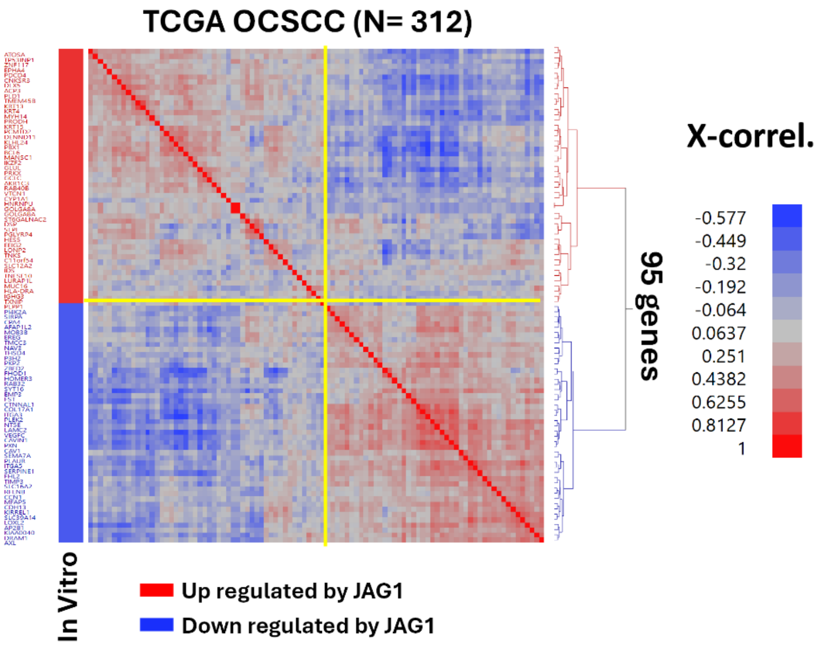

Supplementary Figure 17. Development of an *in vivo* NOTCH1 signaling gene expression signature. **A.** The TCGA OCSCC RNA-seq cohort was mined for cross-correlation of RNA expression using gene candidates from the list of 119 top genes regulated *in vitro* after JAG1 exposure. Hierarchical two-way clustering of cross-correlation coefficients identified two main gene clusters annotated by whether the genes were up-regulated (red box) or down regulated (blue box) after JAG1 exposure. **B.** Re-clustering TCGA OCSCC cross-correlation coefficients with the remaining 95 genes after removal of those with inconsistent *in vitro* and *in vivo* behavior.

Supplementary Figure 18

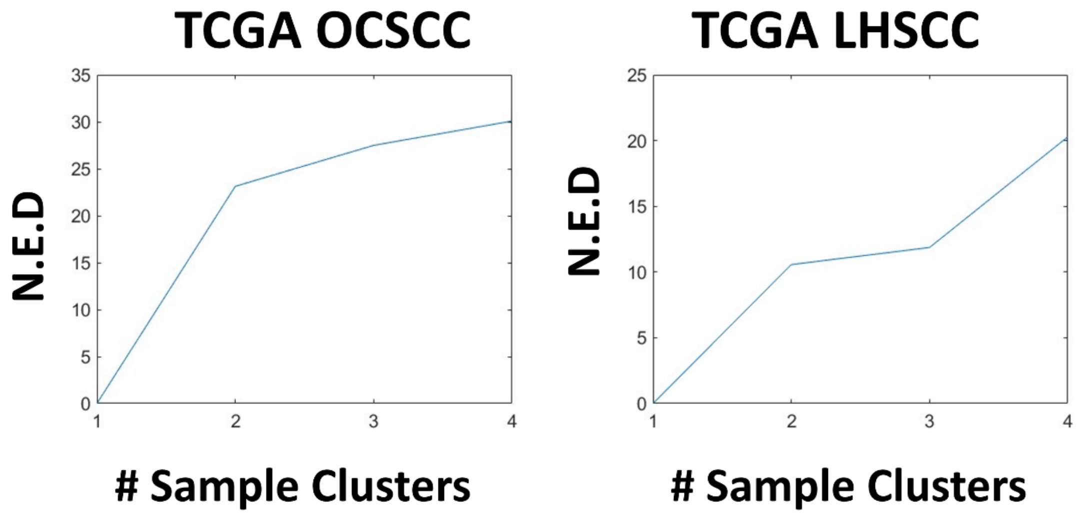

Supplementary Figure 18. Selection of optimal sample clusters numbers with the NOTCH1 gene signature. Consensus hierarchical clustering of TCGA OCSCC and LHSCC samples using the 95 gene NOTCH1 signature was performed and the similarity matrices achieved using increasing numbers of sample clusters (e.g. N=1 to 4) were compared to theoretical perfection matrices to select a local minimum for the normalized Euclidean distances (N.E.D.) to identify an optimal number of sample clusters, which happened to be N =2 clusters for each disease subsite.

Supplementary Figure 19

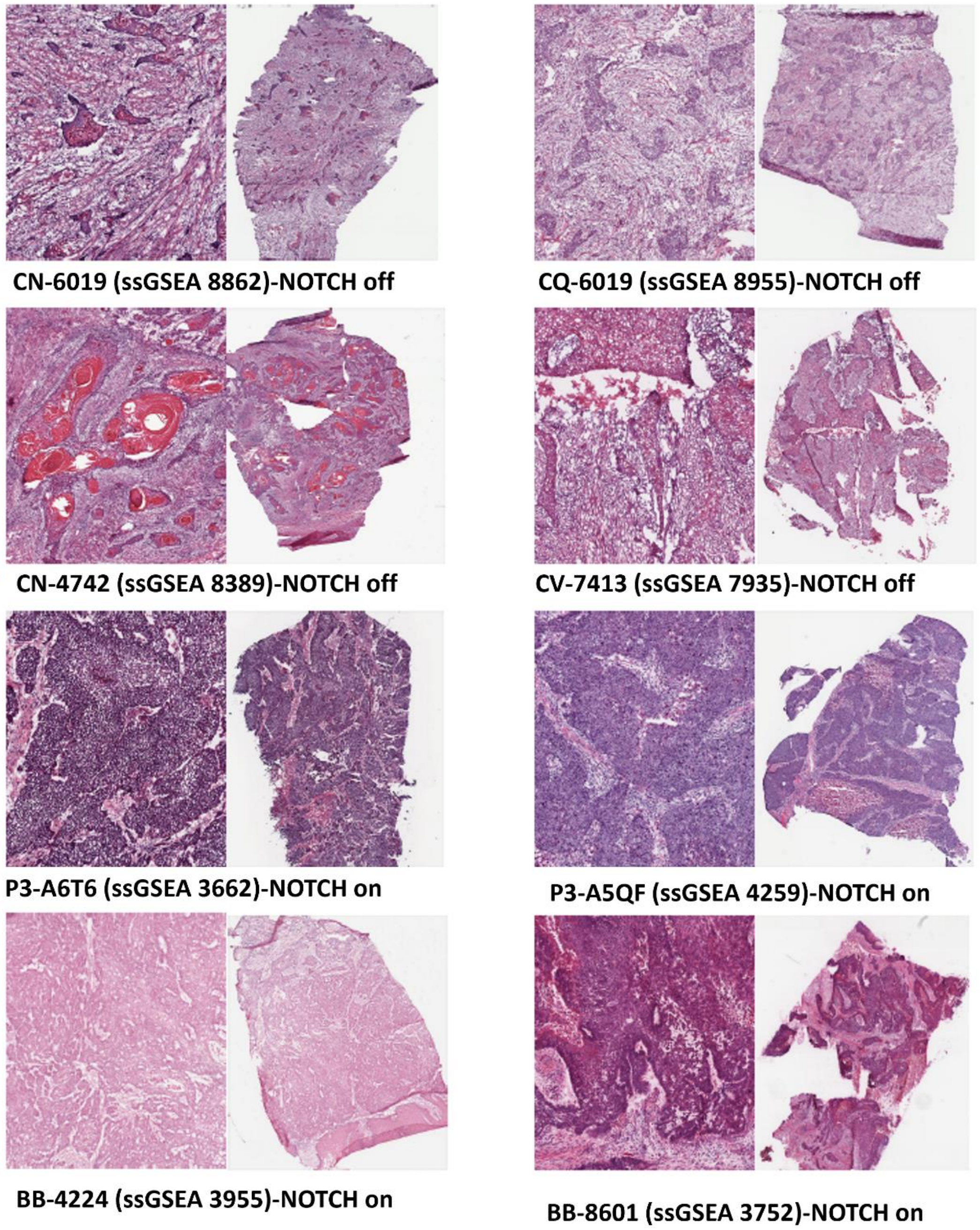

Supplementary Figure 19. Visual confirmation that TCGA tumor samples identified with active NOTCH1 signaling contain fewer CAF. H&E images were downloaded from the TCGA data portal and the CAF ssGSEA scores appear in parenthesis alongside the sample TCGA identification numbers.

Supplementary Figure 20

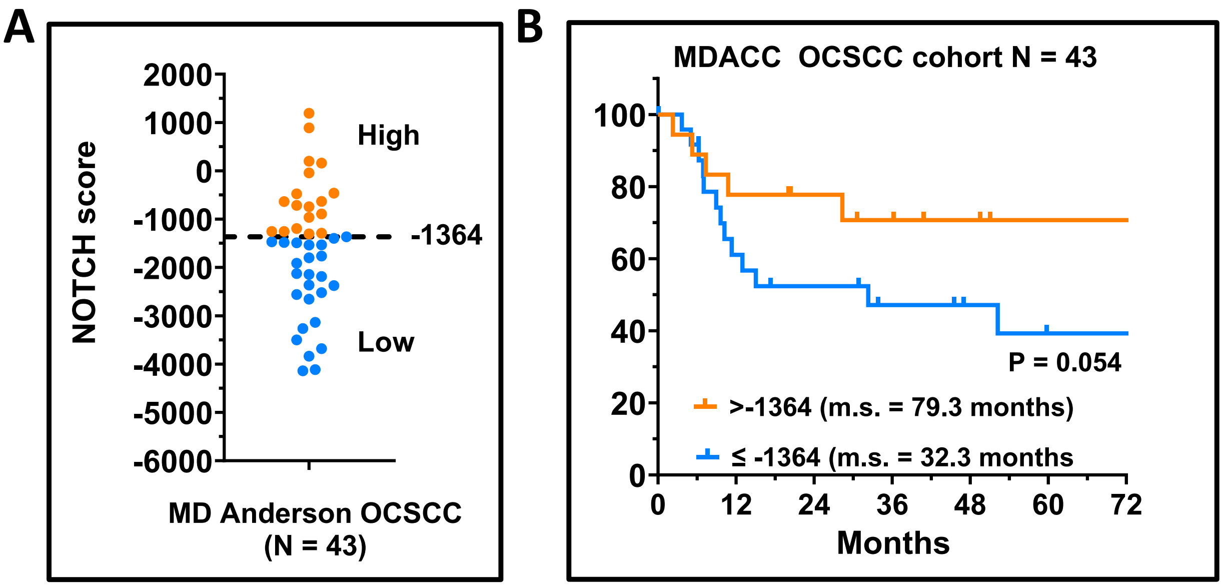

Number at risk (number censored)													
Time	0	12	24	36	48	60	72	84	96	108	120	132	143
NOTCH low	25 (0)	14 (2)	11 (3)	8 (5)	6 (7)	4 (8)	4 (8)	3 (8)	2 (8)	0 (8)	0 (8)	0 (8)	0 (8)
NOTCH high	18 (0)	14 (0)	11 (3)	9 (4)	7 (6)	5 (8)	5 (8)	3 (8)	3 (8)	3 (8)	2 (9)	1 (10)	1 (10)

Supplementary Figure 20. NOTCH activation is associated with better prognosis in an independent validation cohort. A. Distribution of NOTCH scores in the MD Anderson OCSCC cohort with the optimal cutpoint (-1364) shown as a horizontal dotted line. Tumor specimens above the cutoff (high) have orange symbols while those below the cutoff have blue symbols. **B**. Kaplan-Meir curve depicting overall survival and median survival (m.s.) of patients based on their tumors NOTCH score group from panel A. Differences in survival approached significance (P =0.054 by a log rank test). The at-risk table for the Kaplan-Meir data is shown at the bottom of the figure.

Supplementary Figure 21

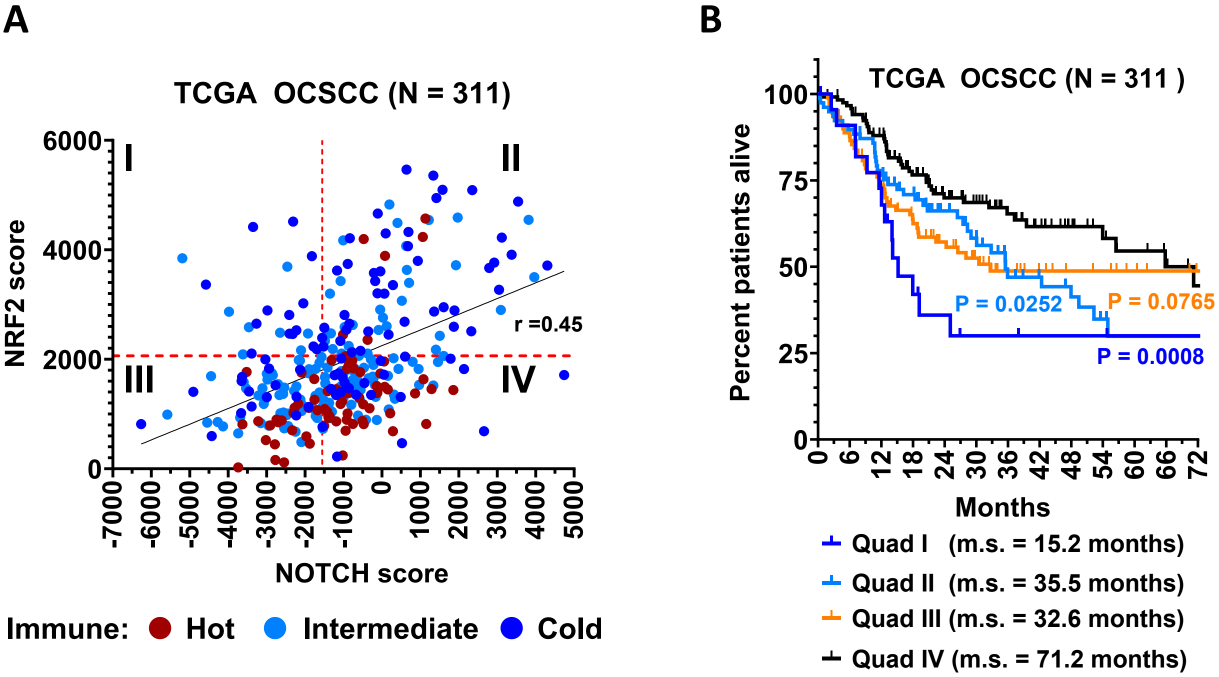

Number at risk (number censored)							
Time	0	12	24	36	48	60	72
Quad I	23 (0)	15 (2)	6 (4)	4 (5)	3 (6)	2 (7)	2 (7)
Quad II	78 (0)	58 (2)	35 (18)	20 (24)	14 (28)	6 (33)	6 (33)
Quad III	90 (0)	62 (5)	38 (16)	21 (28)	14 (35)	9 (40)	5 (44)
Quad IV	120 (0)	99 (7)	59 (29)	37 (48)	22 (61)	14 (67)	8 (71)

Supplementary Figure 21. NOTCH and NRF2 pathways define unique risk groups. A. Patient tumors from the TCGA OCSCC cohort (N = 311) were plotted in two dimensions based on their NOTCH and NRF2 scores determined from ssGSEA. Previous optimal cutpoint thresholds for NOTCH (-1554) and NRF2 (2063) are indicated with dotted red lines to divide tumors into four quadrants: I) low NOTCH/high NRF2; II) high NOTCH/high NRF2; III) low NOTCH/low NRF2; IV) high NOTCH/low NRF2. A linear regression (black line) demonstrates a positive correlation between NOTCH and NRF2 scores (Pearson correlation = 0.45, P <0.001). The immune cluster (hot, intermediate, cold) of each tumor sample from the prior analysis (Fig. 6) is annotated by colored symbols according to the legend. B. Kaplan-Meir survival plots of patients from the four risk quadrants defined in panel A demonstrate that patients whose tumors had low NOTCH but high NRF2 signaling (quadrant 1) had the worst median survival times. Difference in survival curves were analyzed with a log rank test comparing each risk group to quadrant IV which had the best median survival. An at-risk table indicating the number of patients still at risk or censored is depicted underneath.

Supplementary Figure 22

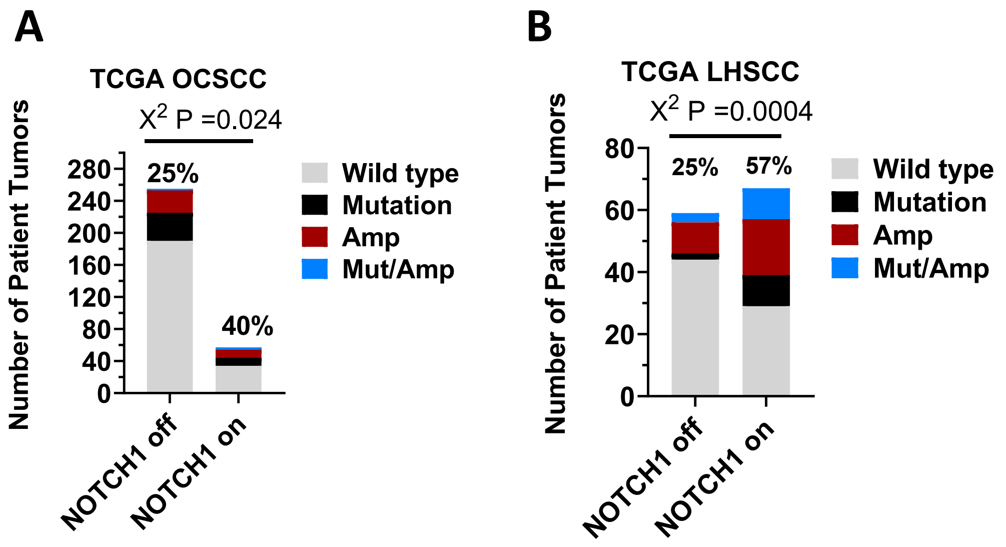

Supplementary Figure 22. Patients lacking NOTCH1 mutations are significantly enriched for genomic alterations in PIK3CA. The proportion of tumors with genomic alterations in PIK3CA (i.e., numbers above bar graphs), including mutations and/or high-level gene copy gains, is significantly higher (ChiSquare test) in in the group associated with NOTCH signaling identified through hierarchical clustering of TCGA OCSCC (A) or (B) LHSCC.

LIST OF ADDITIONAL FILES AND TABLE DESCRIPTIONS

NOTCH_suppelmentary methods.docx: Detailed methods of experimental procedures and statistical analyses.

NOTCH_Supplementary Tables S1 to S14.xlsx:

S1. NOTCH1 and NOTCH2 genotype of established HNSCC tumor cell lines used in the study and their expression of 157 different protein analytes measured by Reverse Phase Protein Arrays.

S2. Protein analytes correlating with cl-NOTCH1 levels across 53 different HNSCC cell lines either WT or mutant for NOTCH1.

S3. Genes downregulated by extended growth on the NOTCH1 ligand JAG1 identified as a main effect or individually for NOTCH1 WT cell lines PJ34 and/or 183.

S4. Genes upregulated by extended growth on the NOTCH1 ligand JAG1 identified as a main effect or individually for NOTCH1 WT cell lines PJ34 and/or 183.

S5. Gene ontology (GO) enrichment analysis of biological processes for the top 120 genes regulated by prolonged growth on the NOTCH1 ligand JAG1.

S6. Annotation of enriched GO pathways for top genes regulated by JAG1.

S7. Comparison of genes significantly altered after ICN1 induction by DOX in PJ34-iICN1 and after growth of parental PJ34 on JAG1 ligand, along with their fold changes.

S8. Genes significantly bound by activated NOTCH1 protein in Chip-seq experiments after induction of iICN1 by DOX in PJ34-iICN1 cells.

S9. List of genes from different sectors of the Venn diagram depicted in Fig. 3F, identifying overlap between genes downregulated by iICN1, growth on JAG1, or bound by ICN1 in chip-seq.

S10. List of genes from different sectors of the Venn diagram depicted in Fig. 3G, identifying overlap between genes upregulated by iICN1, growth on JAG1, or bound by ICN1 in chip-seq.

S11. Frequency of tumor formation from FaDu-iICN1 in presence or absence of DOX treatment and statistical analysis of estimated tumor initiating cell (CSC) frequencies.

S12. The final 95 gene NOTCH1 activation signature list along with their cross-correlation coefficients of expression from the TCGA OCSCC RNA-seq cohort.

S13. Consensus hierarchical clustering of TCGA OCSCC cohort based on 95 gene NOTCH1 activation signature, which predicts the NOTCH1 pathway status.

S14. Consensus hierarchical clustering of TCGA LHSCC cohort based on 95 gene NOTCH1 activation signature, which predicts the NOTCH1 pathway status.

NOTCH_Supplementary Table S15.xlsx:

S15. Significant genes differentially expressed between OCSCC tumors with NOTCH1 signaling active (consensus cluster 2) and inactive (consensus cluster 1).

NOTCH_Supplementary Table S16.xlsx:

S16. Significant genes differentially expressed between LHSCC tumors with NOTCH1 signaling active (consensus cluster 2) and inactive (consensus cluster 1).

NOTCH_Supplementary Table S17 to S21.xlsx:

S17. List of genes commonly upregulated in cells grown on JAG1, the NOTCH1 gene signature, and patient tumors with active NOTCH1 signaling in OCSCC and LHSCC TCGA cohorts.

S18. List of genes commonly downregulated in cells grown on JAG1, the NOTCH1 gene signature, and patient tumors with active NOTCH1 signaling in OCSCC and LHSCC TCGA cohorts.

S19. List of gene signatures defining cell types present in the tumor microenvironment.

S20. Comparison of ssGSEA scores for leukocyte subtypes, CAFS, and endothelial cells among TCGA OCSCC tumors with NOTCH1 signaling on and off.

S21. Comparison of ssGSEA scores for leukocyte subtypes, CAFS, and endothelial cells among TCGA LHSCC tumors with NOTCH1 signaling on and off.

S22. Cox proportional hazards analysis of survival as a function of NOTCH and NRF2 scores

S23 Logistic regression analysis of tumor immune phenotype as a function of NOTCH and NRF2 scores.

MC_Full_westerns_7_10_25.pdf: Contains full blot images for all western blot figures

SUPPLEMENTAL METHODS:

Notch activation

For NOTCH activation experiments, tissue culture wells were pre-coated overnight at room temperature (RT) with Protein G (Prospec, East Brunswick, NJ) at 50 ug/ml, washed twice in phosphate buffered saline (PBS), blocked in 1% BSA/PBS for 2 hours at RT, washed three times with PBS, coated with either human recombinant chimeric Jag1 fused to an FC fragment (R&D Systems, Minneapolis, MN) or control purified IgG FC protein (Jackson ImmunoResearch, West Grove, PA) at 2 ug/ml in 0.1% BSA/PBS for 3 hours RT, stored overnight at 4°C, and washed three times immediately before use.

Viral vectors, shRNA Vectors, and siRNA Reagents

A 7.3 kb cDNA encoding WT full length human NOTCH1 receptor (NFL1) with a Kozak sequence was provided by Origene (Rockville, MD) and digested with EcoR1/XhoI to subclone into an empty retroviral vector MigR1 (Addgene, Watertown, MA), where the multiple cloning site (MCS) that had been previously modified to contain MfeI (EcoR1 compatible) and Xho1 sites from 5' to 3'. Sanger sequencing confirmed that the entire NFL1 cDNA insert matched the reference sequence for full length human NOTCH1 (NM 017617) encoding 2555 amino acids. The MCS of MigR1 is upstream of an IRES-EGFP (enhanced green fluorescence protein) cassette so NFL1 cDNA is expressed from the same mRNA as EGFP, allowing purification of infected cells by flow cytometry. An ICN1 construct encoding human NOTCH1 Pro1770 to Lys2555 cloned into MigR1 was obtained from Dr. Patrick Zweidler-McKay (MD Anderson, Houston, TX). To express inducible activated cleaved NOTCH1 that could be recognized by cl-NOTCH1 antibodies we utilized RNA from *NOTCH1* WT FaDu to generate a cDNA fragment encoding Val1754 to Lys2555 which was amplified with primers containing a Kozak sequence and artificial ATG methionine start site. Amplified cl-NOTCH1 cDNA was then cloned into the tetracycline/doxycycline lentiviral plasmid pLVX-TRE3G-mCherry using an In-Fusion cloning kit (Takara). GIPZ shRNAs used for targeting AXL and a-CATULIN (CTNNAL1) were from Horizon and included, V2LHS_239988 (AXL, AACTTAGATGCTTAGGATC), V3LHS-329652 (AXL, TGAGGATGGAGTCGTCCTG), V2LHS-239988 (CTNNAL1, TATTTCCAGAGGTTCTGTC), and V3LHS_356693 (CTNNAL1, TGTTTTCTTGCTTTGAGCT). SMARTPool siRNA mixtures targeting either human HES1, HES2, HES5, HEY1, and HEY2 were purchased from Horizon. CRISPR-Cas9 NOTCH1 (sc-400167-KO-2), and NOTCH2 (sc-401323-KO-2) plasmids were obtained from Santa Cruz.

ICN1 Retroviral Construct Validation

The ICN1 fragment encoded by this vector (Pro1770 to Lys2555) is missing several N-terminal amino acids at the cleavage site and is therefore not recognized by antibodies specific for activated NOTCH1. Construct integrity was confirmed through DNA sequencing and detection of its protein product after infecting NOTCH1-null mutant UMSCC22A with an antibody that binds the C-terminus of NOTCH1 (Supplementary Fig. S6A).

Antibodies

Antibodies to cl-NOTCH1 (#4147), AXL (#8661), SOX2 (#23064) and LAMC2 (#53884) were purchased from Cell Signaling Technology (Danvers, MA). Antibodies to total NOTCH1 (Sc 6014), total NOTCH2 (sc 5545), α-Catulin (sc 390584) were from Santa Cruz Biotechnology (Santa Cruz, CA). The β-Actin antibody (A1978) was bought from Sigma (St Louis, MO), and anti-ITGA3 (#PA5-143239) was purchased from ThermoFisher (Waltham, MA).

Viral Mediated Ectopic Gene Expression and shRNA Knockdown (KD)

Retroviral and lentiviral particles were generated by transfecting expression plasmids into helper cell lines—HEK-293GP2 for retroviruses and HEK-293F17 for lentiviruses. For retroviral production, a mixture of MigR1, ICN1, or NFL1 (10.8 mg) with 1.2 mg of the packaging plasmid pCMV-VSV-G (Addgene) was transfected into a 10 cm dish of 6 million adherent HEK-293GP2 cells using GenJet Plus (SignaGen, Frederick, MD) under a modified protocol that minimized volumes during the initial 45 minutes at 37 °C in a tissue culture incubator. After this period, complete media was added for an additional 8 hours at 37 °C before the transfection reagents were washed out. The cells were then incubated at 33 °C to optimize viral production, and the virus-containing supernatants were harvested at 48 and 72 hours, centrifuged, and pooled. Lentiviral particles (e.g., shRNA or inducible expression plasmids) were produced similarly, using a mixture of 10 μg expression plasmid, 5 μg packaging plasmid pCMV-dr8.2 (Addgene), and 5 μg envelope plasmid pMD2.G (Addgene) to transfect HEK-293F17 cells with GenJet Plus. The cells were incubated at 37 °C, and virus was harvested from the supernatants at 48 and 72 hours.

Cells were infected with retroviral or lentiviral particles using a reverse spin inoculation protocol. Briefly, trypsinized tumor cells were counted and mixed with serial dilutions of viral supernatant, fresh media, and polybrene (final concentration of 8 μg/mL). One million cells were then seeded in 2 mL per well in 6-well plates. The plates were centrifuged at 12,000 × g for 1.5 hours at room temperature, then transferred to a 37 °C tissue culture incubator for 48 hours. Infected cells were subsequently purified by flow cytometry or, in some cases, selected using antibiotics. Cell lines expressing DOX-inducible lentiviral constructs were

generated by first infecting cells with pLVX-Tet3G (Takara, San Jose, CA) encoding the Tet-ON 3G regulator protein and selecting for G418 resistance to obtain Tet3G modified cells. Cells stably expressing Tet3G were then infected with human ICN1 that had been subcloned into pLVX-TRE3G-mCherry (iICN1) and selected for 1 week in puromycin. For some experiments, polyclonal puromycin selected cells were used while in other cases, cell sorting was used to generate and screen clones with minimal leakiness and maximal ICN1 induction.

Mouse Tumor models

Cell lines infected with equivalent titers of shRNA lentivirus (AXL, α-CATULIN, or empty vector) were sorted for GFP positivity by flow cytometry, allowed to recover for 2 days and then 50,000 cells (in 30 µl PBS) were injected into the anterior tongues of anesthetized mice using a 30-gauge needle. For flank models utilizing shRNA-infected tumors or iICN1-inducible cell lines (FaDu-iICN1 or UMSCC22A-iICN1), 3-4 million cells were injected subcutaneously into flanks of nude mice in 200 µl PBS. For some groups, mice were administered 1 mg doxycycline (DOX) dissolved in 200 µl water by oral gavage 5 days per week for 1 to 3 weeks.

Analysis of RNA expression

Total RNA was isolated from replicate samples of PJ34 or 184 cells grown for 5 days on plates coated with either JAG1 or control FC. The RNA isolated with Trizol was purified by ethanol precipitation and hybridized to Affymetrix HuGene 2.0 ST arrays by the MD Anderson Sequencing and Microarray Core Facility. Data was processed using aroma.affmetrix package in R to quantify the CEL files with Robust Multiarray Averages (RMA) , apply background correction, quantile normalization, and RMA Probe-level summarization. The processed data was log2 transformed before analysis of differentially expressed genes (DEGs) using a linear model fit with both treatment and cell line as fixed effects. Two-sided P values were modeled using a beta-uniform mixture model and combined with false discovery rate at 0.05 to determine P value cutoffs.

RNA-seq was used to identify genes differentially expressed after iICN1 expression in PJ34. Replicate cultures of either PJ34-Tet3G or PJ34-iICN1 cells were incubated in the presence or absence of 1000 ng/ml DOX for 36 h and total RNA isolated with a RNeasy Kit was sequenced by the MD Anderson Sequencing and Microarray Core Facility. Gene expression was normalized as counts per million and log2 transformed before identifying differentially expressed genes using the response screening module in JMP19 statistical software, which conducts individual two-tailed T-tests for every gene and applies a B-H correction (FDR = 0.1, significance cutoff) to calculate adjusted P values. To minimize the number of tests, poorly expressed

genes were filtered out before the analysis by removing any gene whose average for at least one treatment group failed to exceed a low expression threshold (e.g., log2 expression < 2). Data from control Tet3G cells were used to identify and exclude any genes regulated by DOX alone, in the absence of iICN1 expression.

Consensus Hierarchical Agglomerative Clustering

Z scores from select genes were employed in a two-way consensus hierarchical agglomerative clustering analysis using Ward's minimum variance method, implemented via a custom Matlab script (available at https://github.com/aif33/Hierarchical-two-way-agglomerative-consensus-clustering) that we previously described (30). This approach is based on a modification of the resampling method described earlier by Monti et al., wherein 80% of the samples are randomly selected without replacement in each iteration. For each resampled set, Ward's clustering partitions the samples into N clusters, with N varied over a user-specified range (e.g., 2, 3, 4, etc.), and the frequency with which any two samples co-occur in the same cluster is recorded in a similarity matrix. This matrix is then transformed by retaining the original similarity values for pairs that consistently cluster together and replacing the values for pairs that do not with one minus the similarity value, effectively representing the fraction of iterations in which the samples did not co-cluster. Ideally, this transformation would yield an identity matrix, with all off-diagonal values equal to one, indicating perfect separation. The deviation of the observed transformed matrix from this ideal is quantified by computing the Euclidean distance between the two matrices, which is then normalized by dividing by N to yield a normalized Euclidean distance (NED). The optimal number of clusters is determined by identifying a localized minimum on the NED versus cluster number plot, thereby balancing the tradeoff between increasing cluster granularity and the preservation of meaningful information. For graphical representation, the untransformed similarity matrix was subsequently used for Wards clustering (JMP13) in each dimension to generate dendrograms that robustly depict how samples or features cluster and should be ordered for a given choice of N clusters, which can then be overlayed aside the heatmap generated using the original Z scores. For two-way clustering one set of Z scores calculated from the same dimension (e.g. across samples for each gene) are independently subjected to consensus clustering to define the dendrograms and order for both features and samples, which are combined to generate a final heatmap.

Chip-seq

For Chip-seq experiments, PJ34-iICN1 cells were seeded into six T175 flasks at 6 million cells each and on the following day cells were treated with 1000 ng/ml doxycycline for 36 h to induce ICN1 expression before scraping and processing cells for Chip-seq according to our detailed published protocol (45). Briefly, the processed sample was divided into two equal parts. One part was incubated with a rabbit monoclonal antibody against cl-NOTCH1 (Cell Signaling, #4147) to immunoprecipitate ICN1 cross-linked to DNA,

while the other part was treated with purified Rabbit IgG as a negative control for background signal subtraction. Following washes and reverse crosslinking, DNA was eluted, purified, quantitated and used to generate libraries for next generation sequencing performed on a HiSeq 3000 instrument. Raw reads were aligned to hg19 using Bowtie, 30 million reads were randomly sub-sampled, and peaks called with the Model-based Analysis for Chip-seq tool in Python.
