# Supplementary material for: NOTCH1 acts as a tumor suppressor that induces early differentiation in head and neck cancer
Source: JCI Insight. 2026 Apr 16;11(11):e202414. doi: 10.1172/jci.insight.202414 (PMC13313506; doi:10.1172/jci.insight.202414)

Figure 1

Fig. 1A left

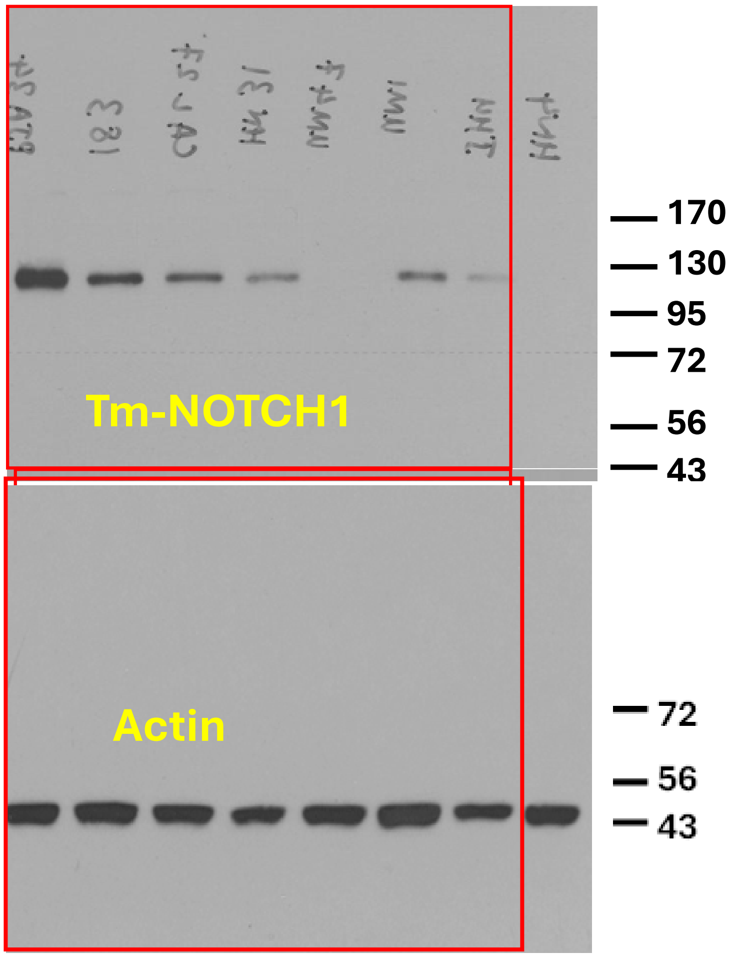

Fig. 1A right 183 & CAL27

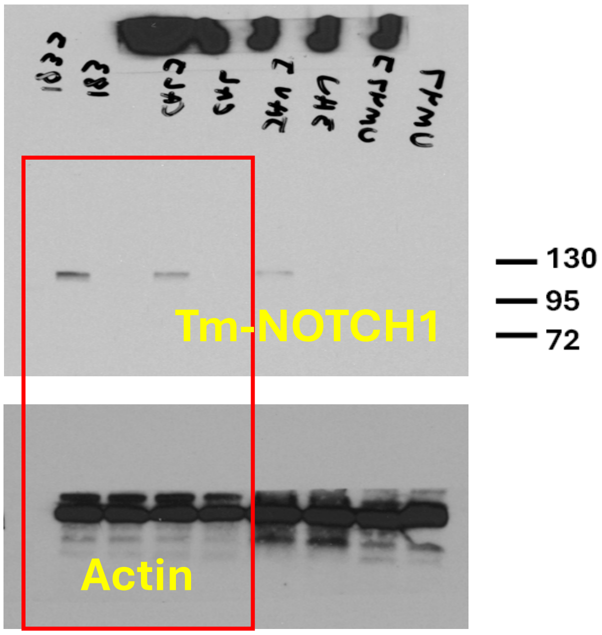

Fig. 1A right PJ34

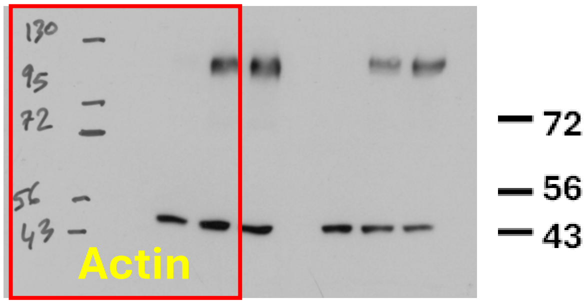

Fig. 2A cl-NOTCH: FaDU, SCC61, MDA1986

Figure 2A

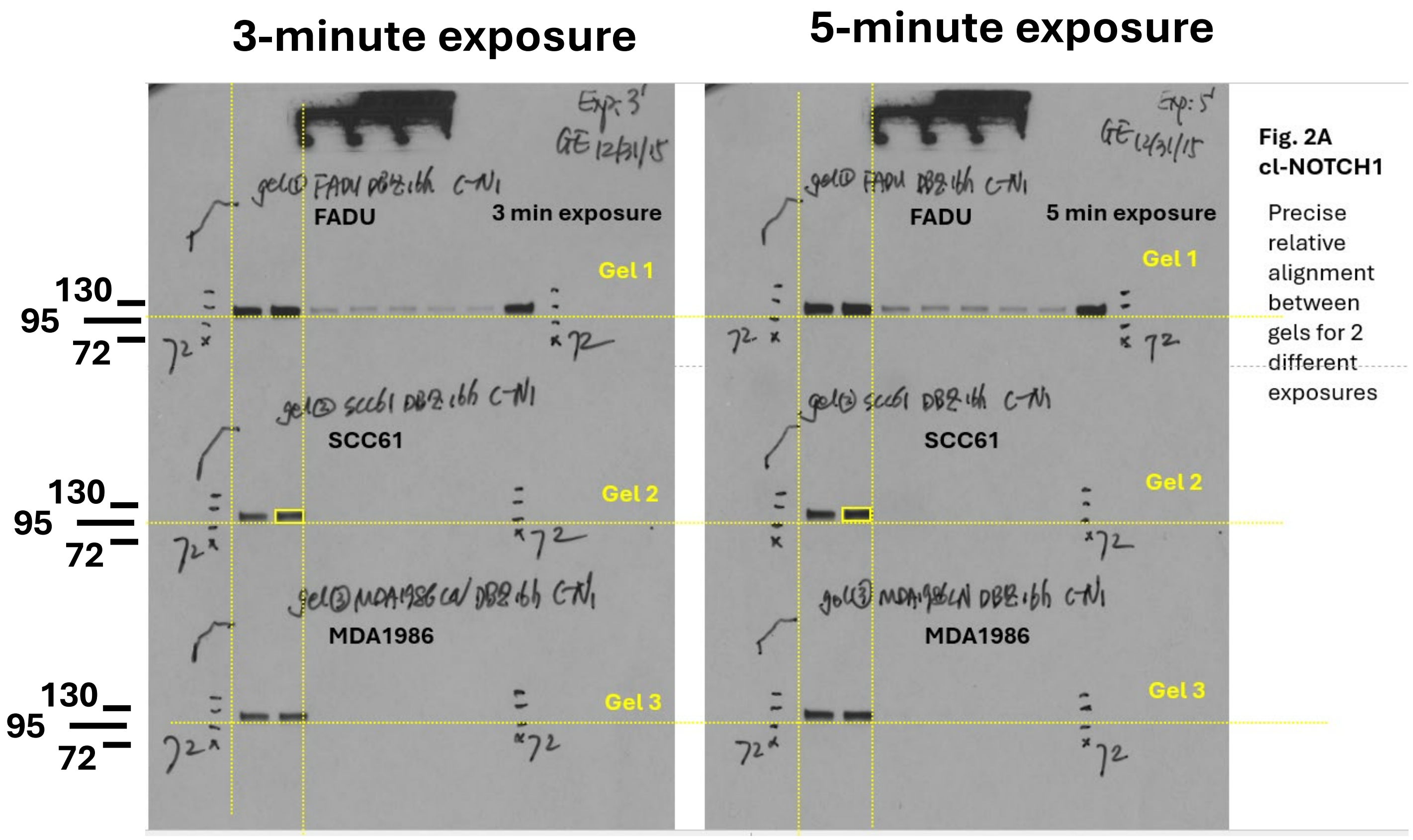

Fig. 2A cl- NOTCH1: SCC15 and PCI-24

Figure 2A

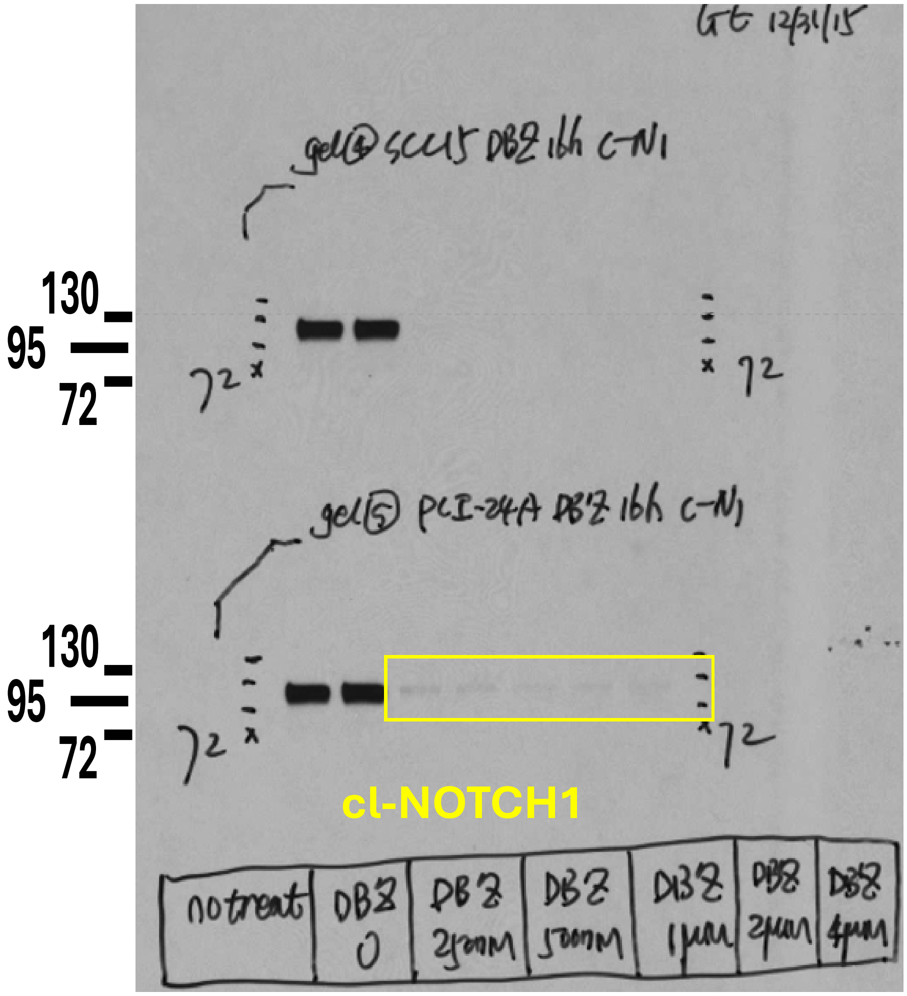

PC-24 samples have Faint bands for high Doses of DZB treatment Compared to SCC15

Fig. 2A Actin: FADU, SCC61, MDA1986LN, SCC15, and PCI-24

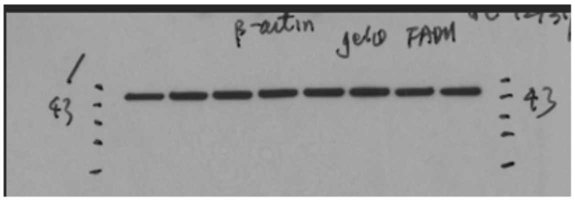

Fig. 2A
Actin

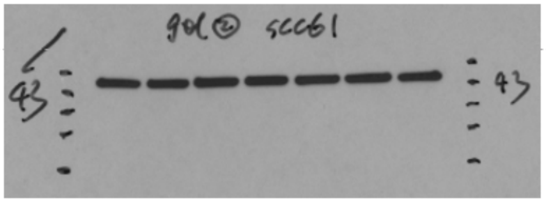

Fig. 2A
Actin

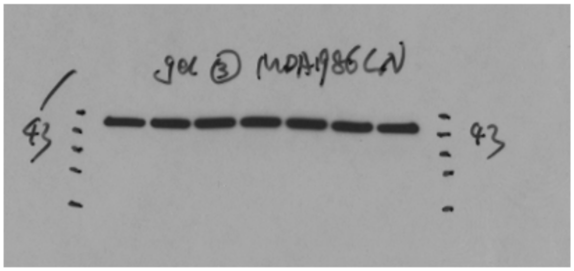

Fig. 2A
Actin

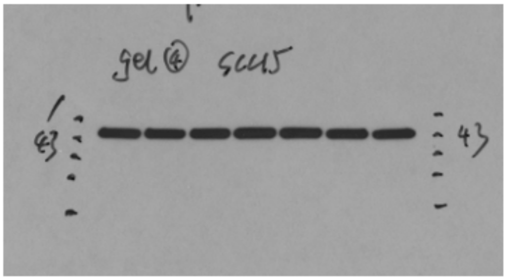

Actin

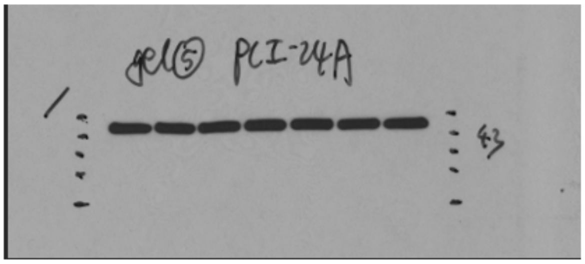

Actin

Figure 3B

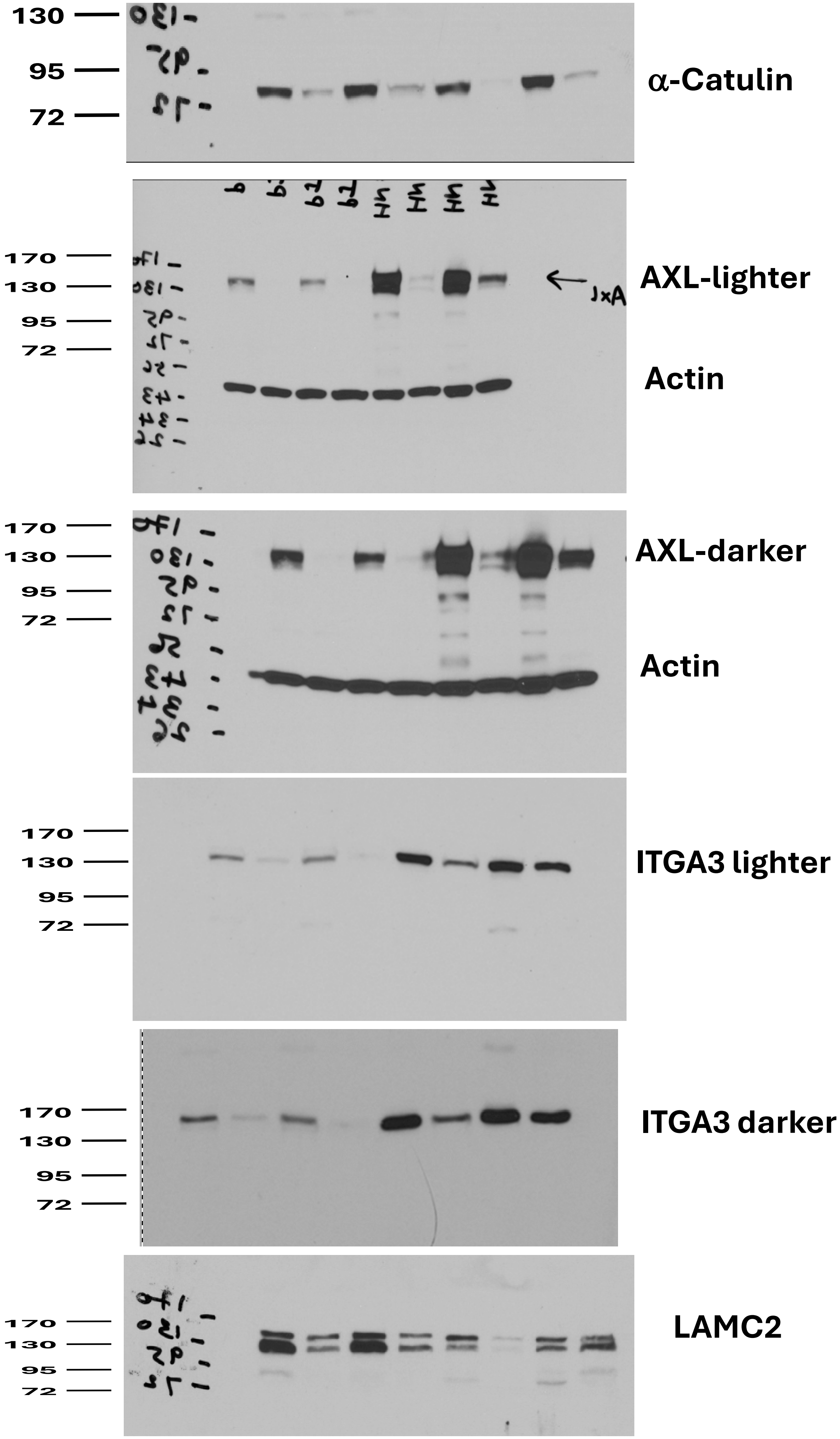
α-Catulin
AXL-lighter
Actin
AXL-darker
Actin
ITGA3 lighter
ITGA3 darker
LAMC2
170
130
95
72

Figure 3C- PJ34

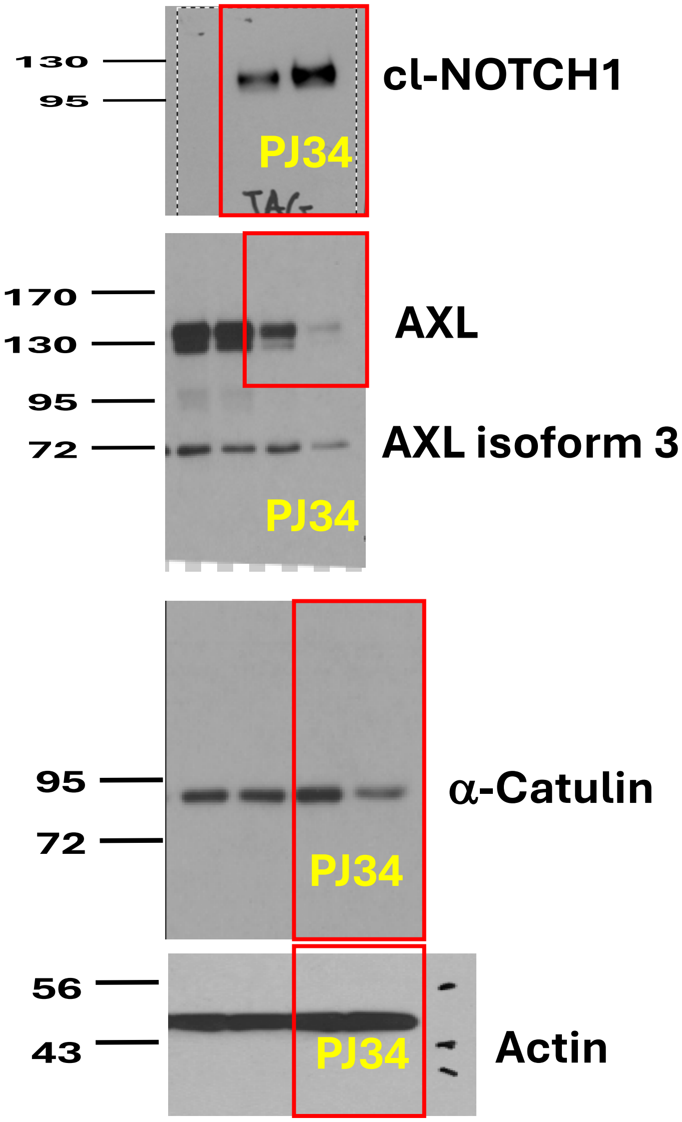

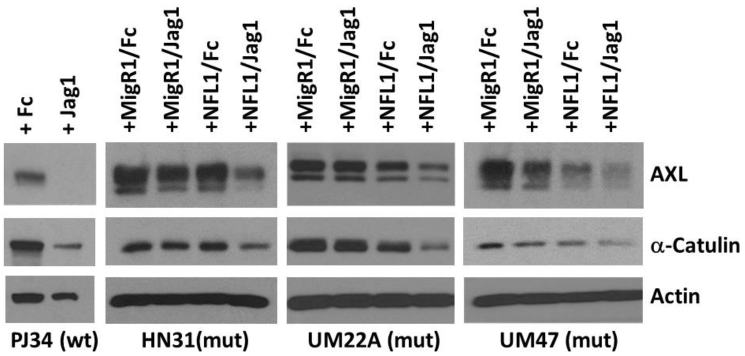

Figure 3C- HN31

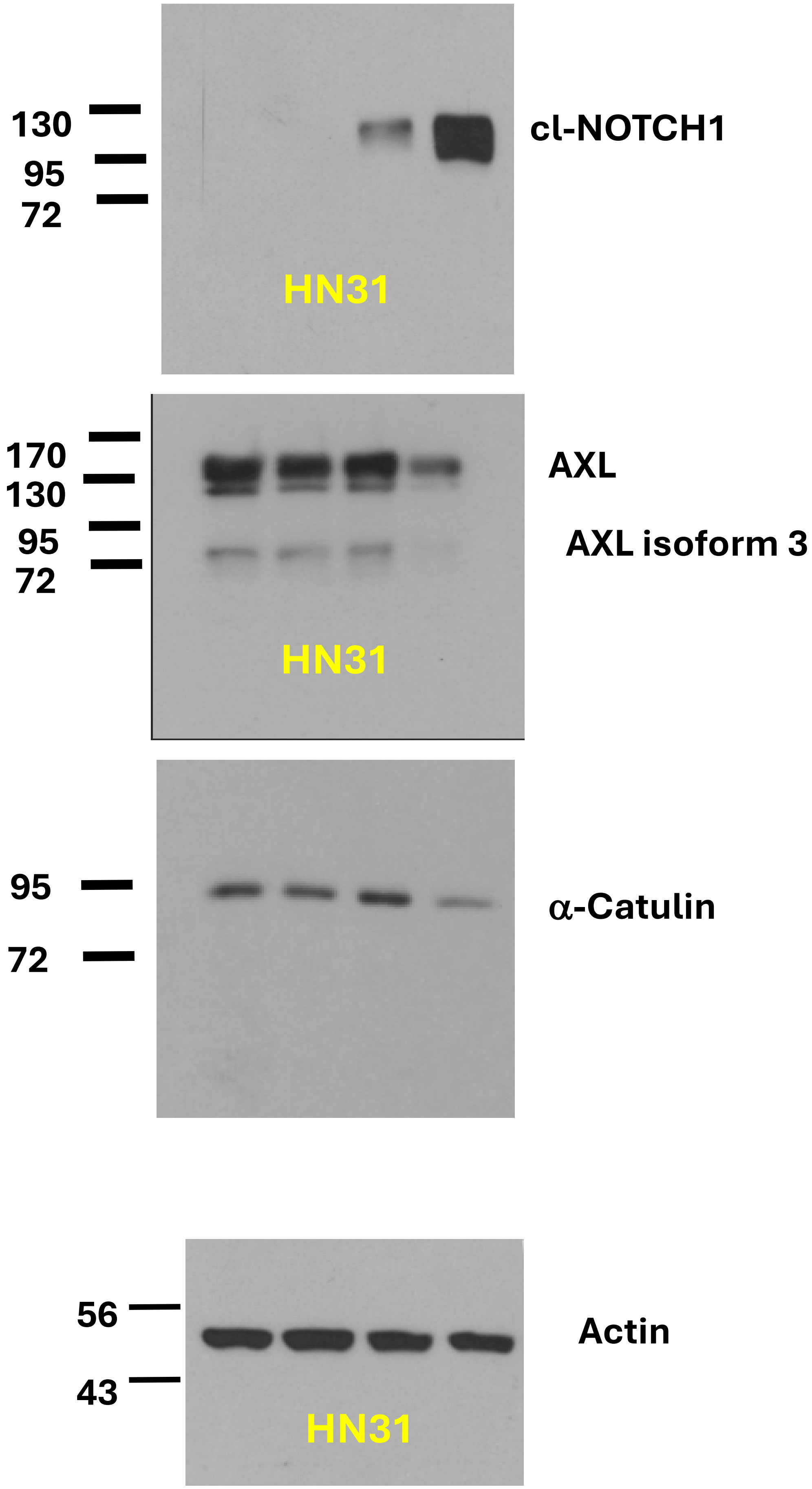

Figure 3C- UM22A

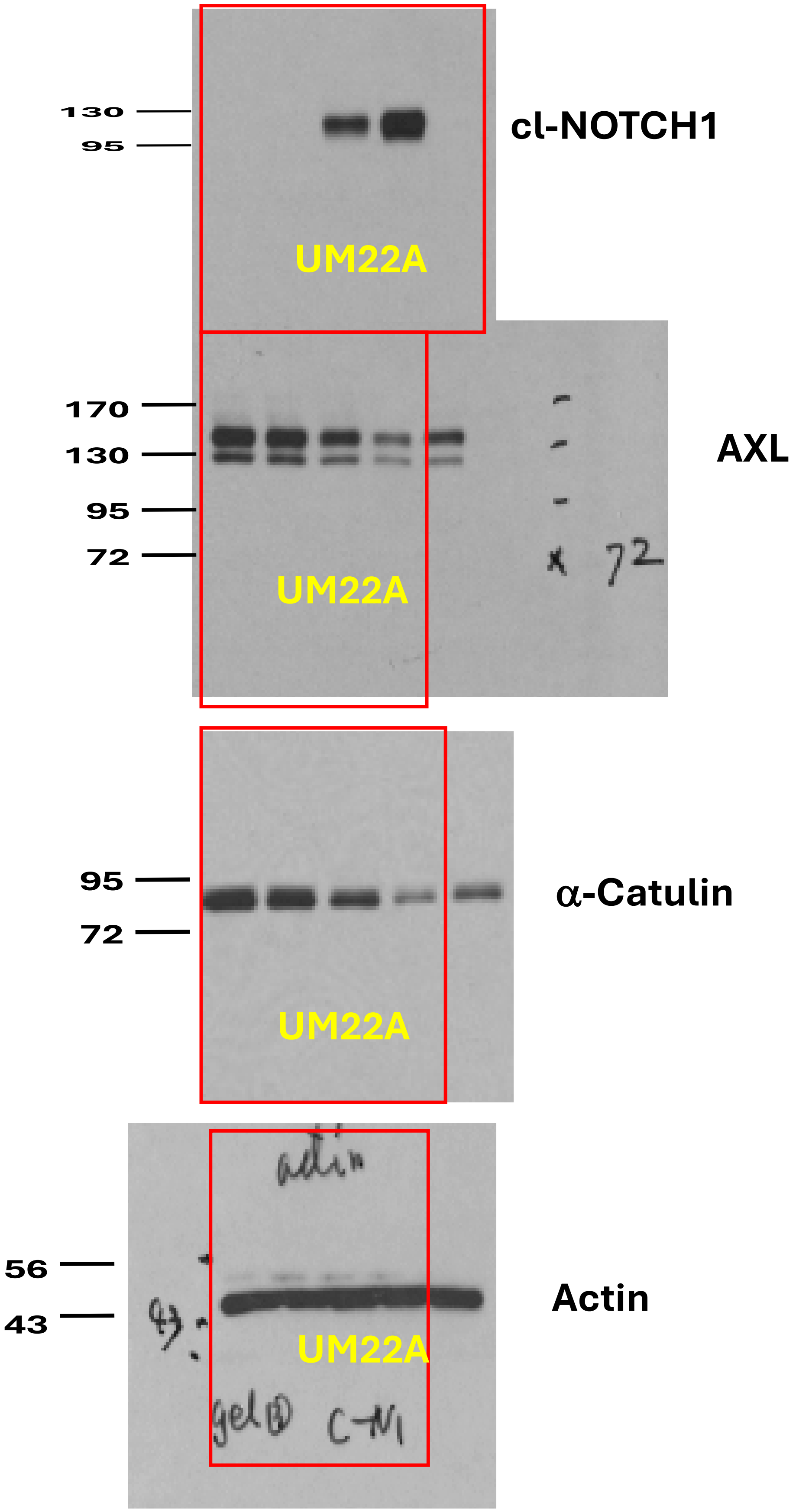

Figure 3C- UM47

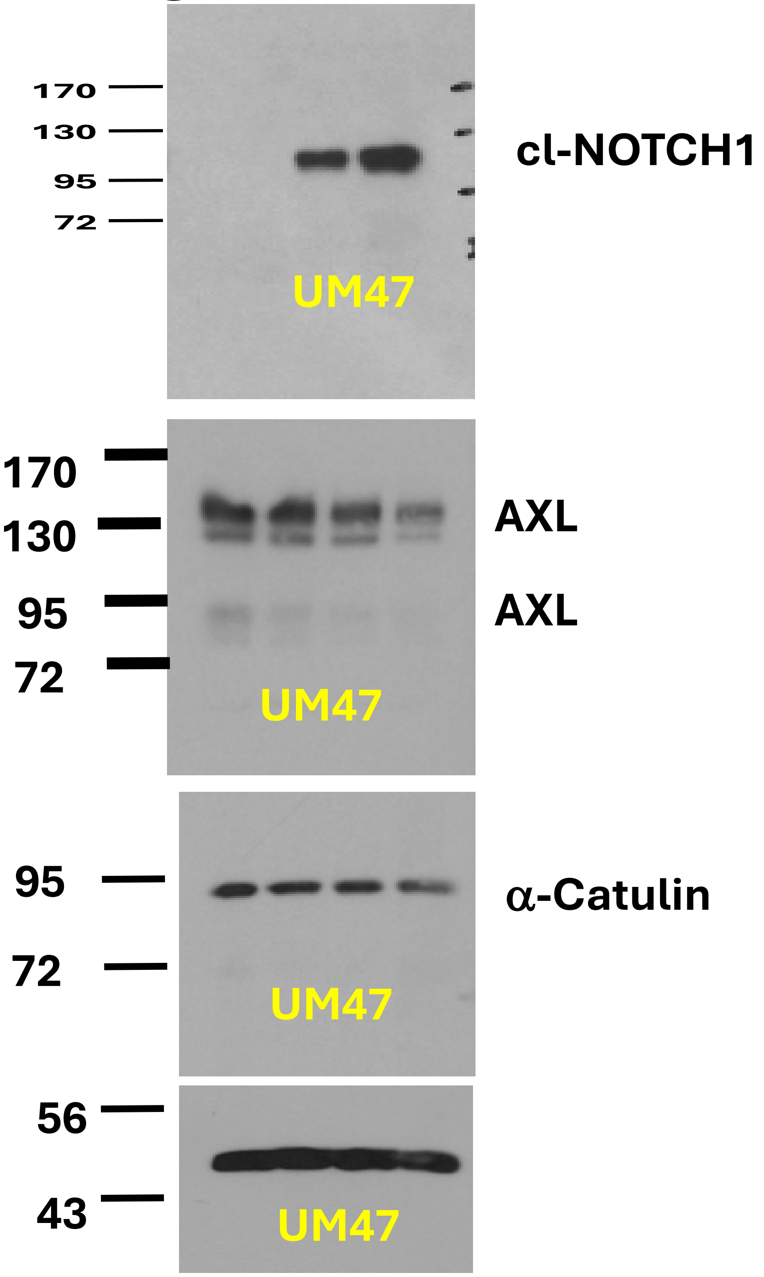

Figure 3D

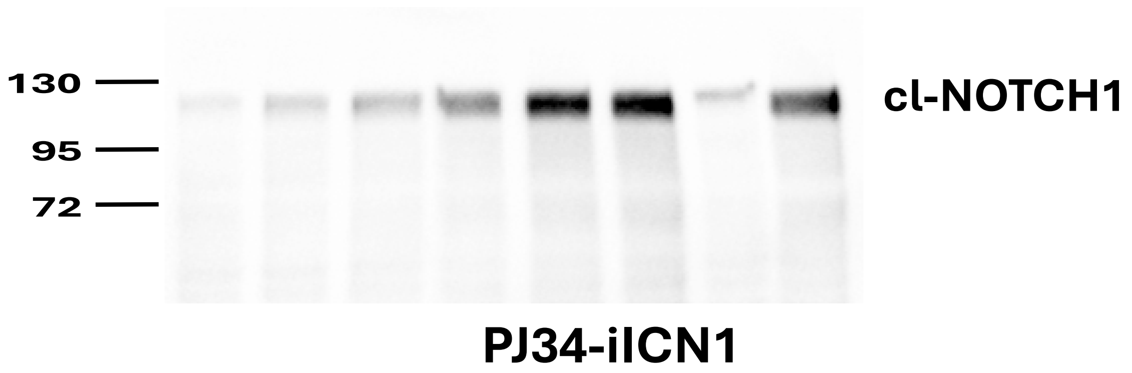

Figure S1 A

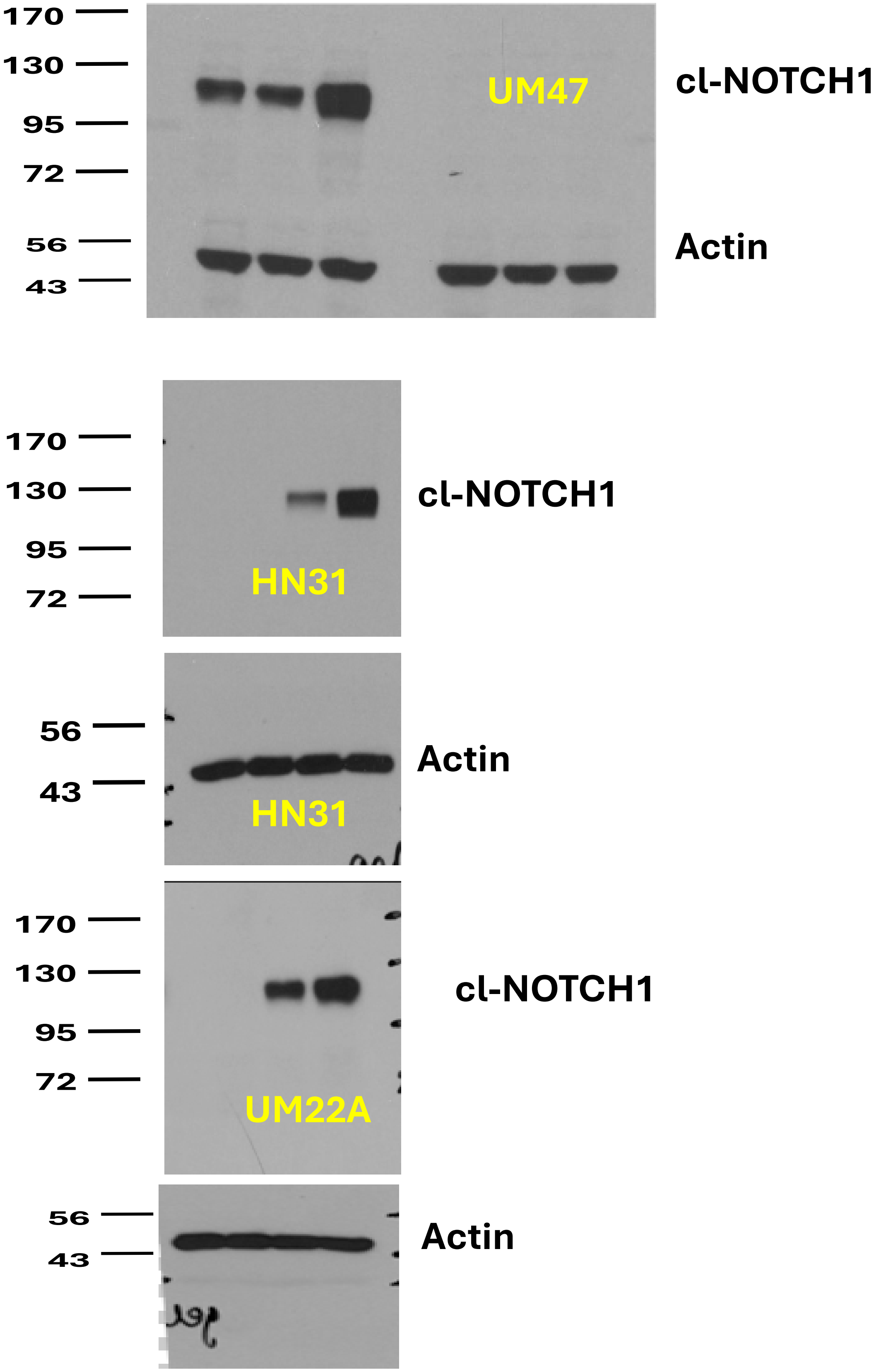

170
130
95
72
56
43
UM47
cl-NOTCH1
Actin
HN31
cl-NOTCH1
56
43
HN31
Actin
UM22A
cl-NOTCH1
Actin

Supplementary Figure 3A

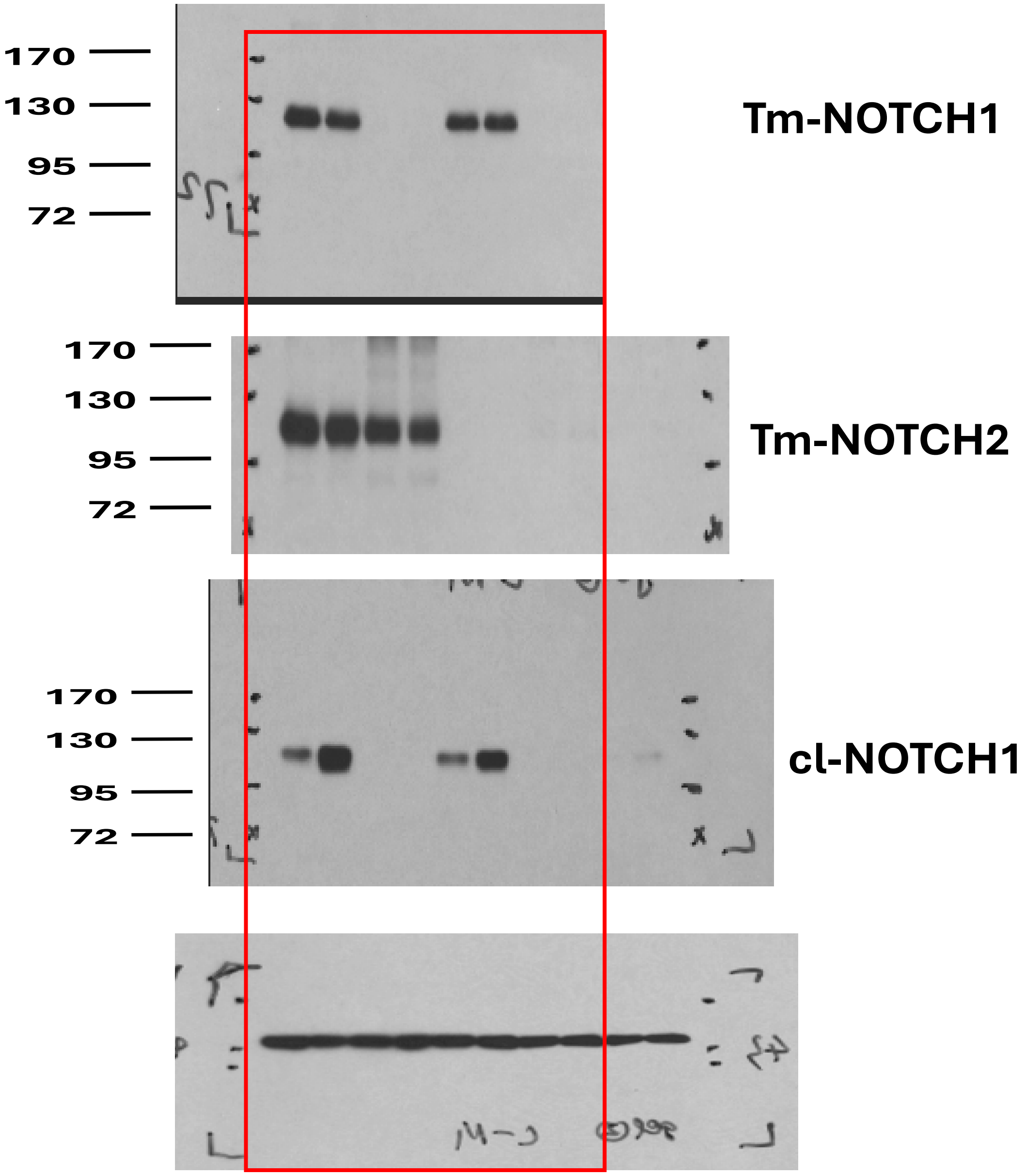

Supplementary Figure S3C

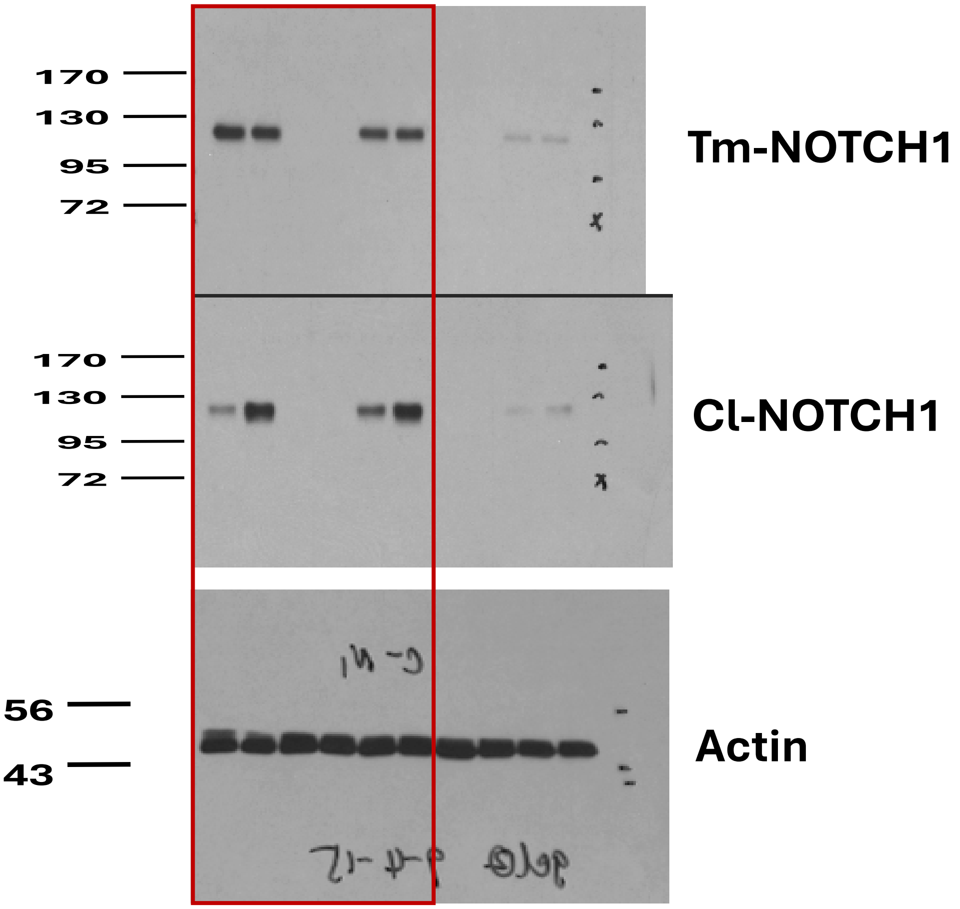

Supplementary Figure S4B

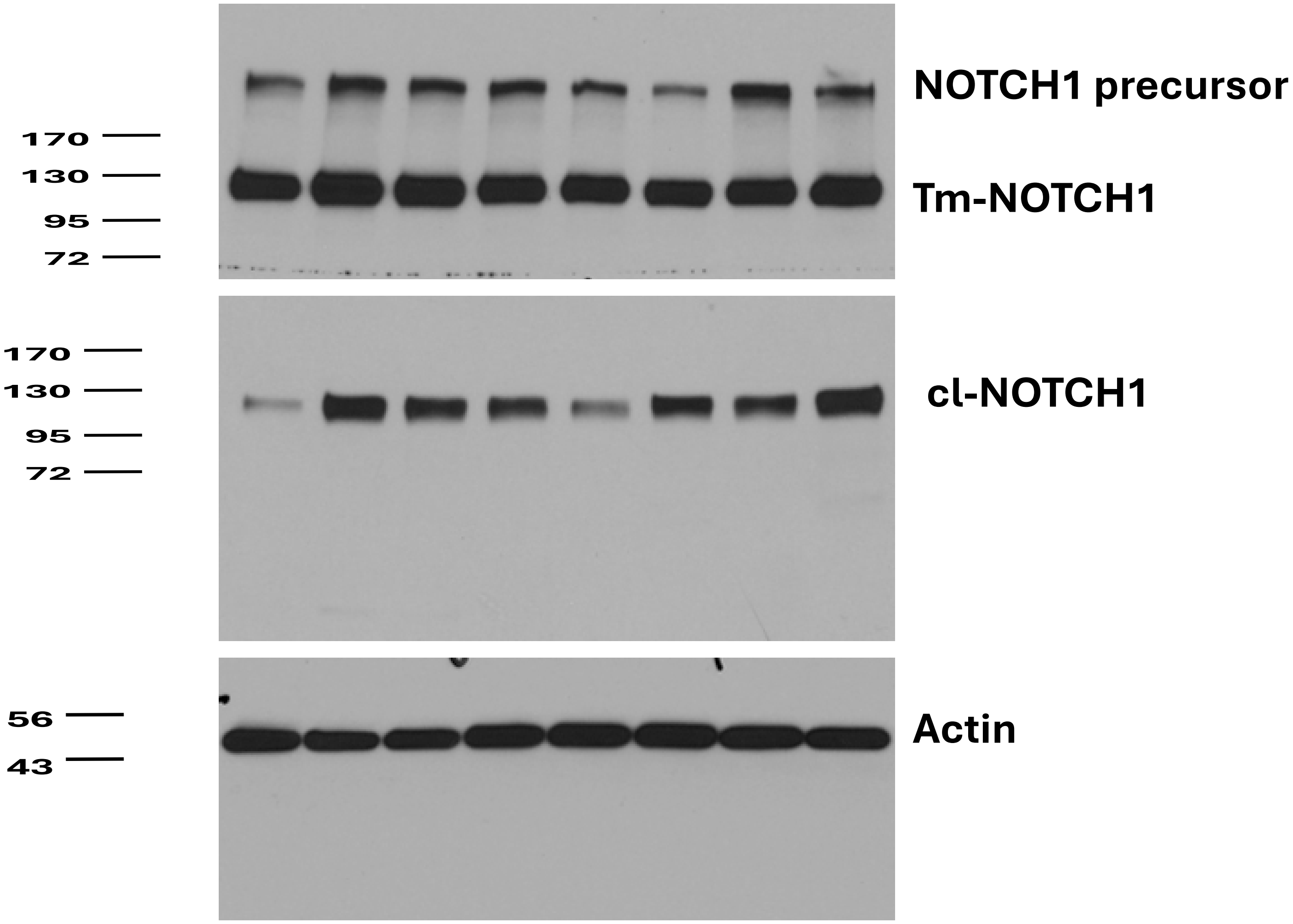

Supplementary Figure S4C

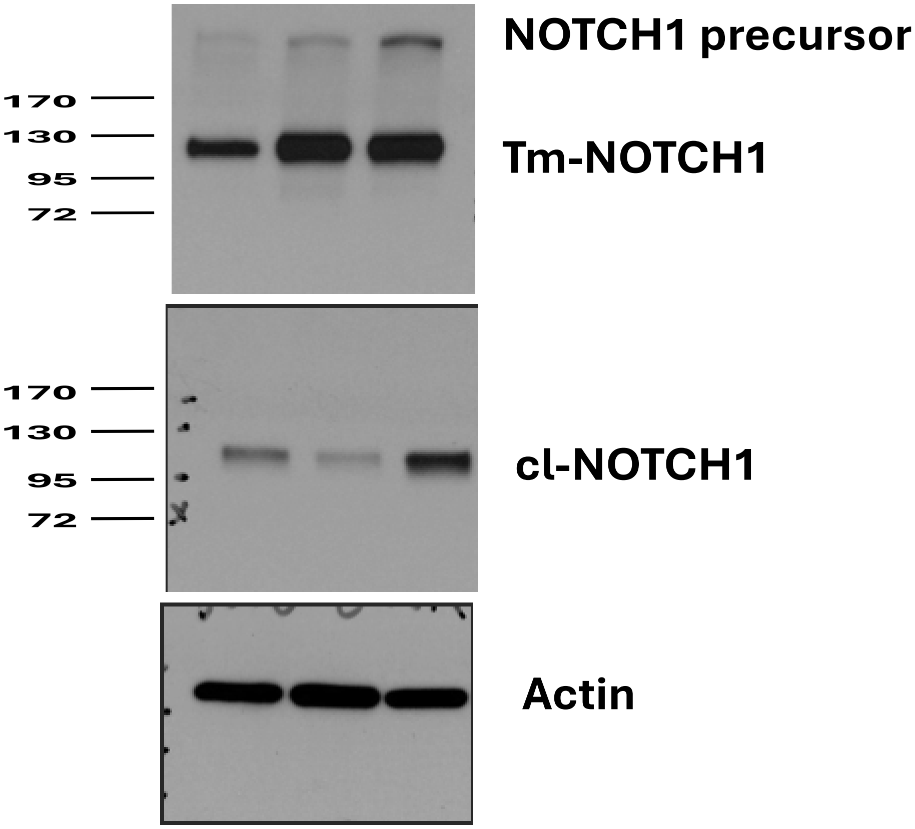

Supplementary Figure 4D-MDA686LN

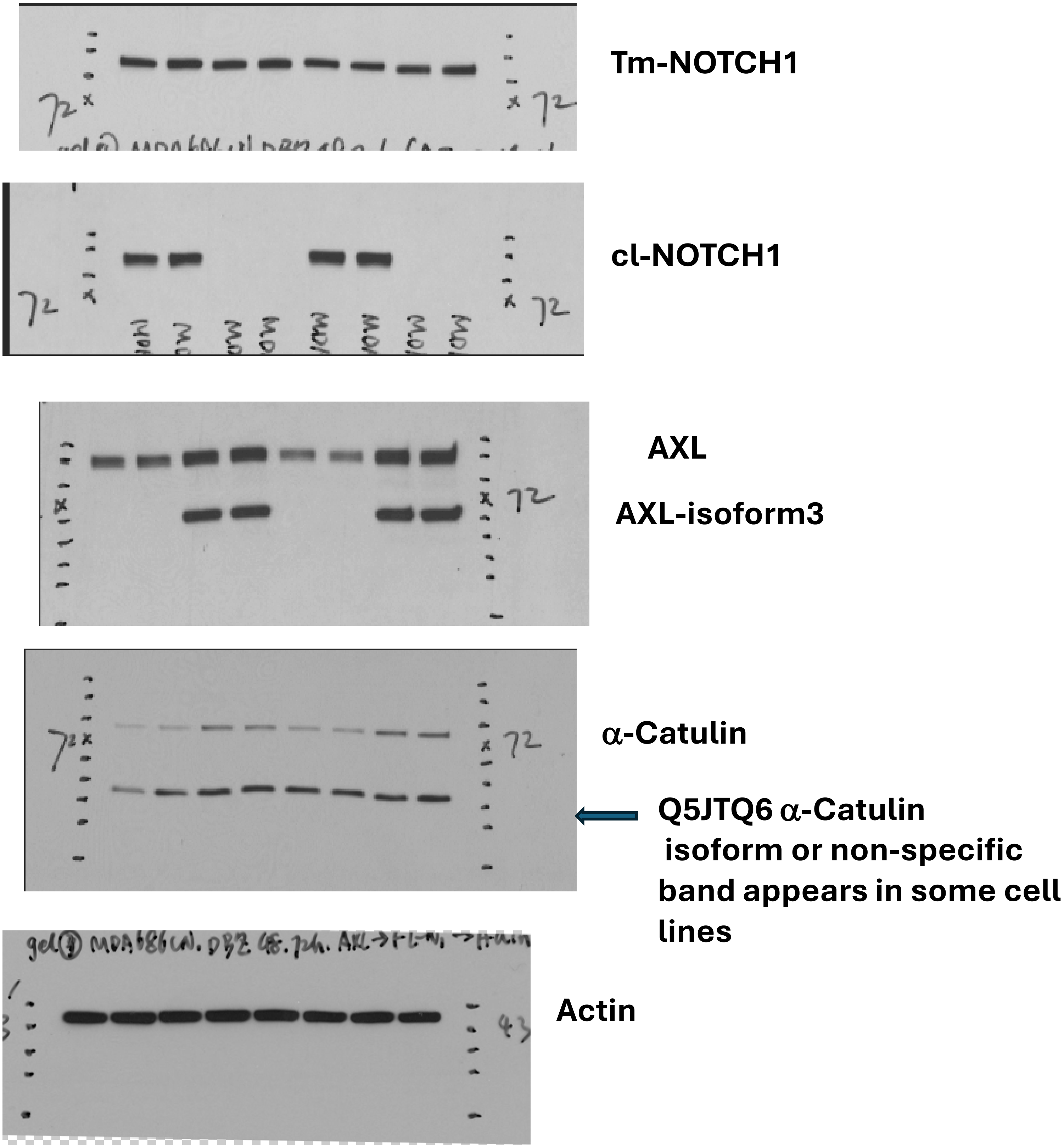

Supplementary Figure S6A and S6E

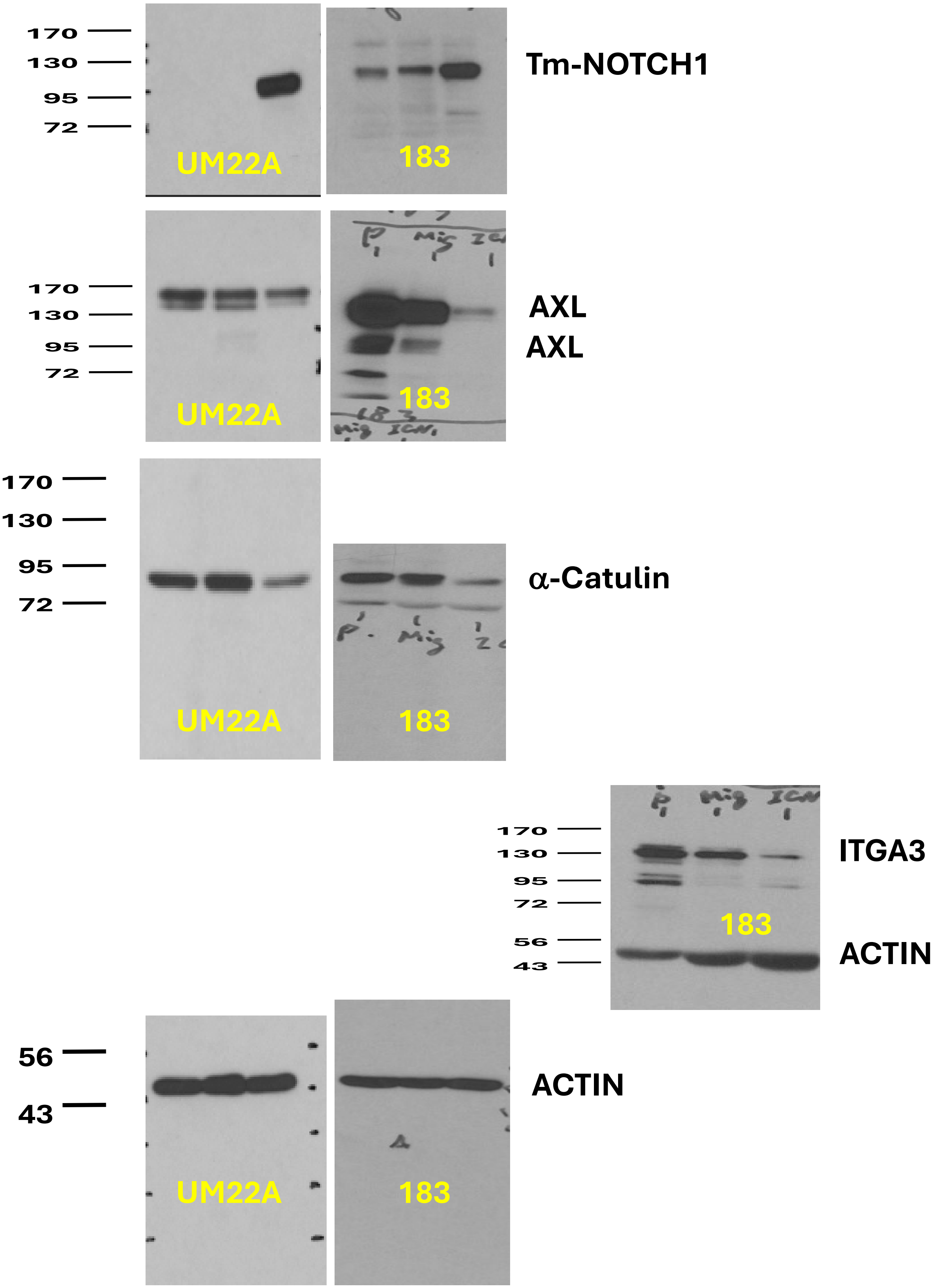

Supplementary Figure S7-FaDu

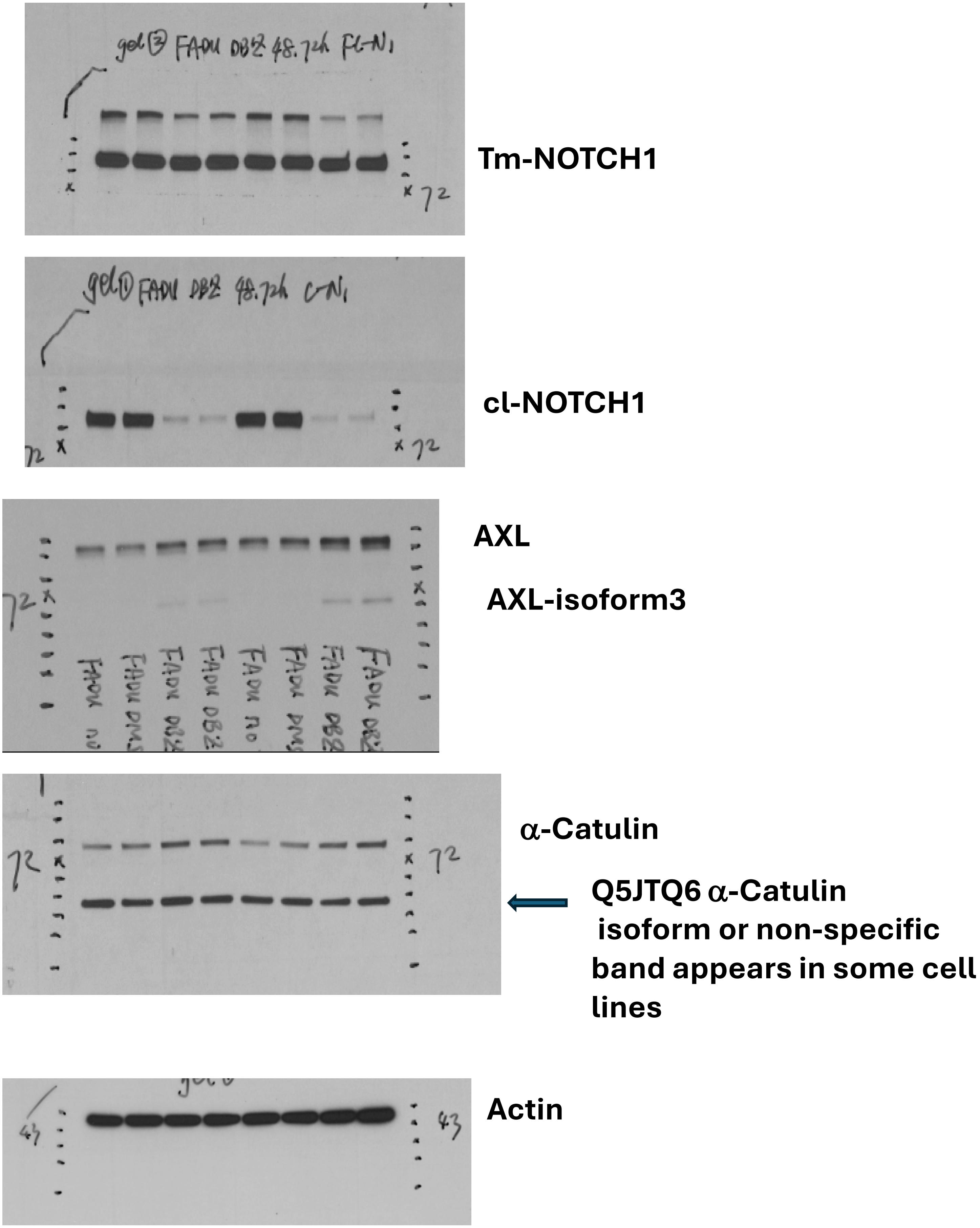

Supplementary Figure S7-PCI24

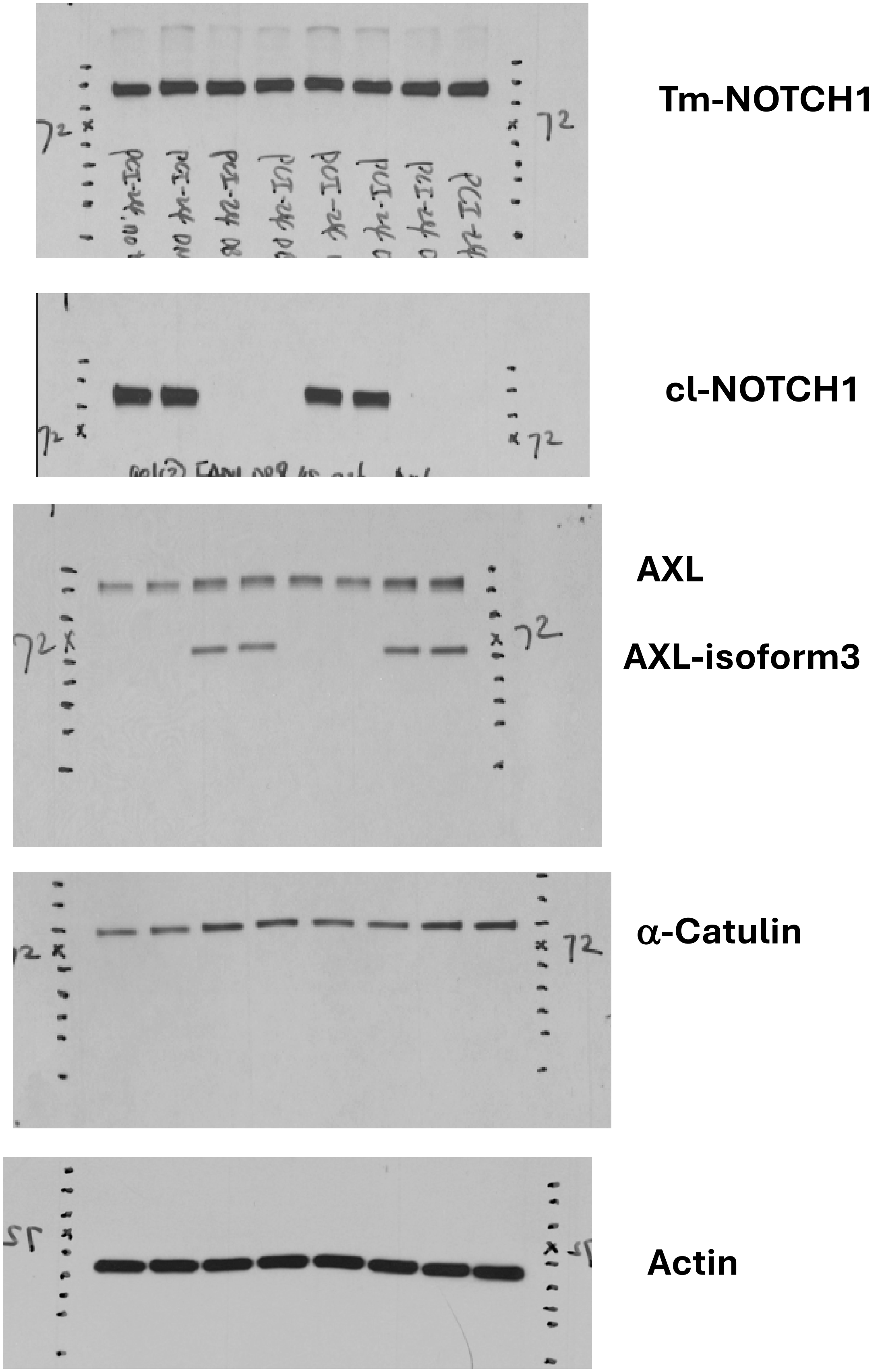

Supplementary Figure S7-SCC61

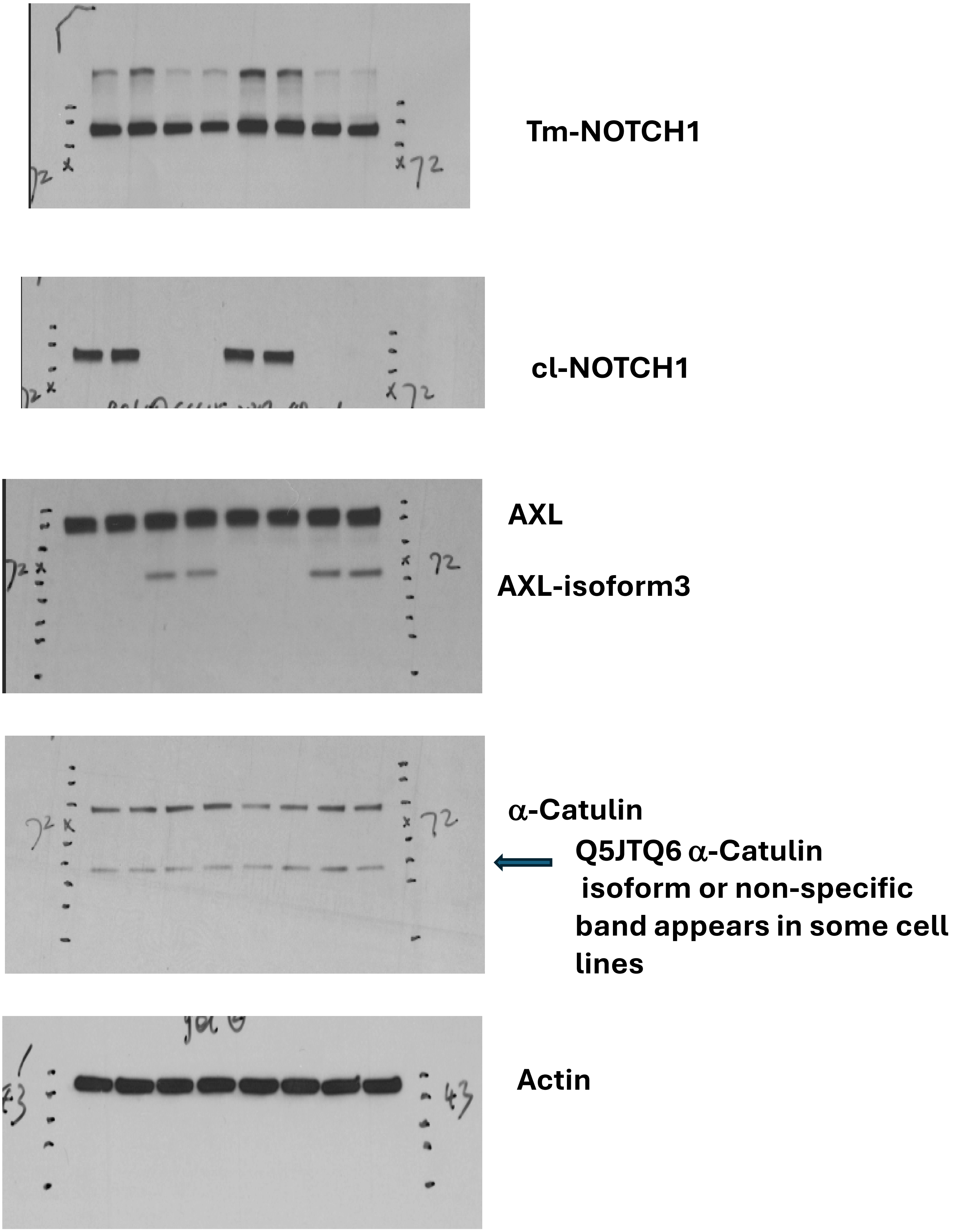

Supplementary Figure S7-SCC15

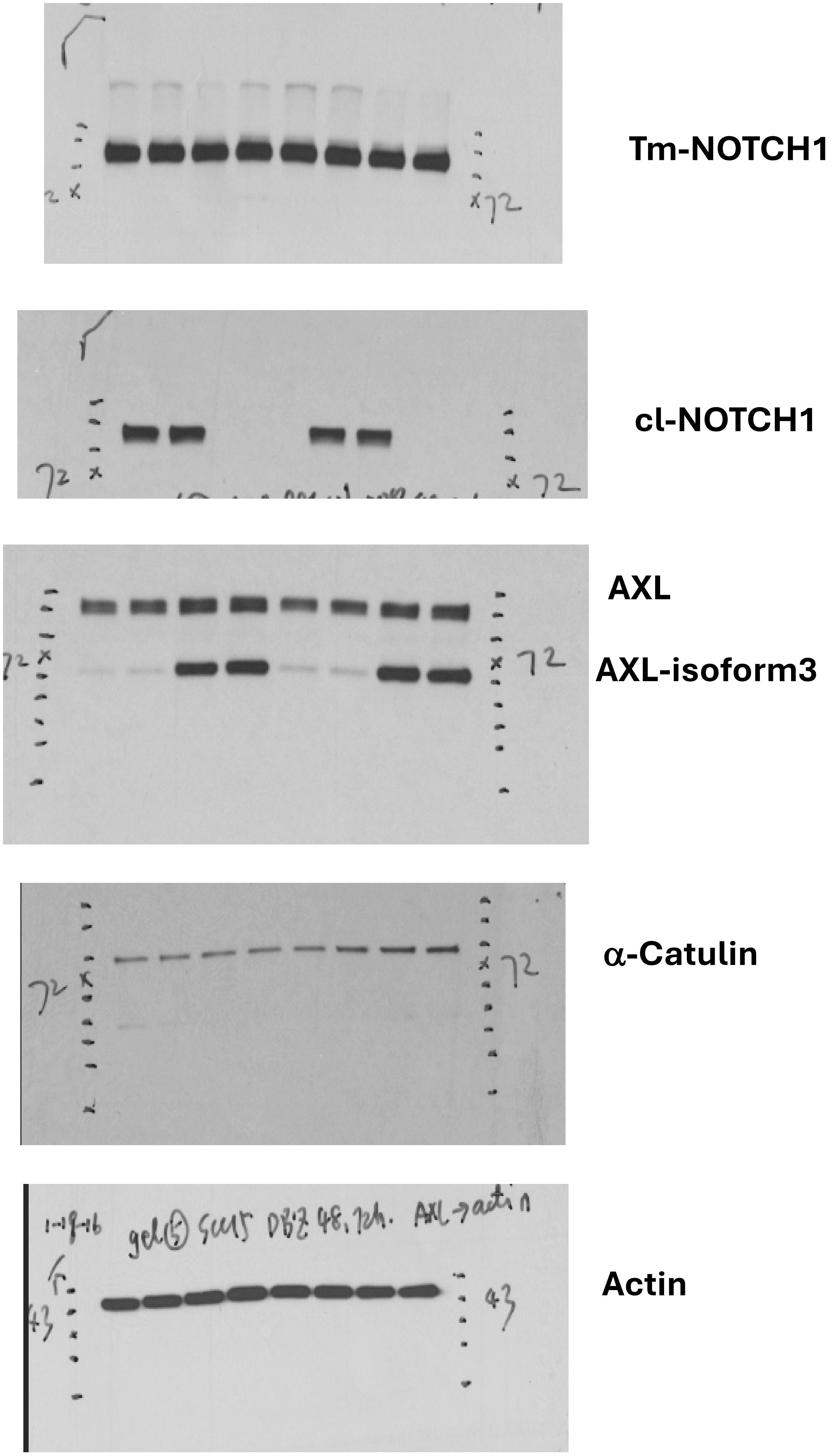

Supplementary Figure S7-MDA1986LN

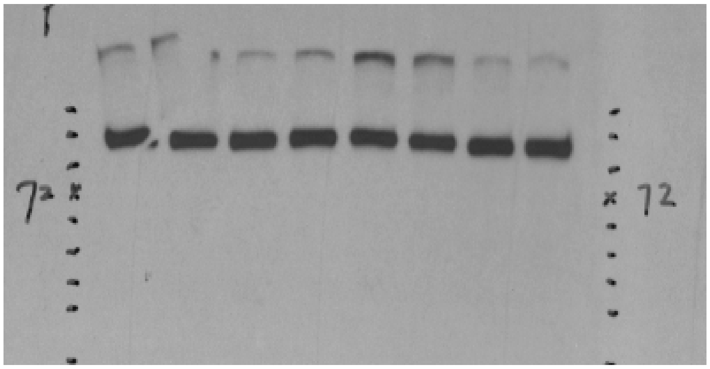

Tm-NOTCH1

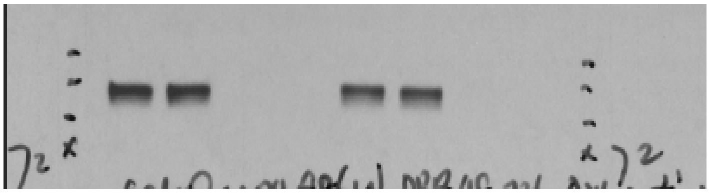

cl-NOTCH1

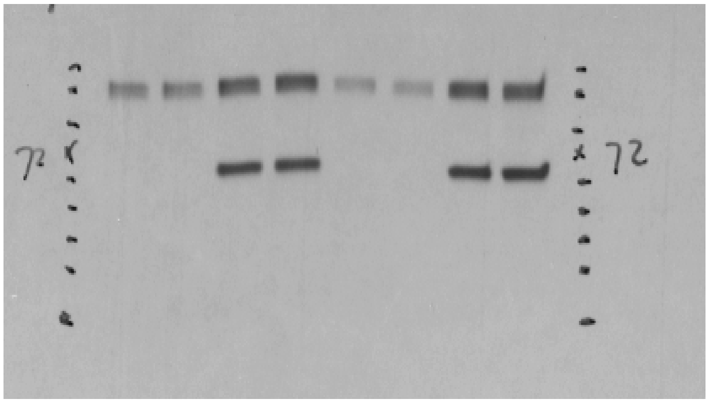

AXL

AXL-isoform3

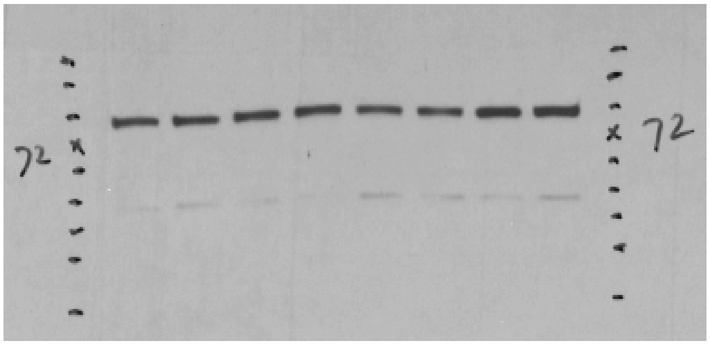

α-Catulin

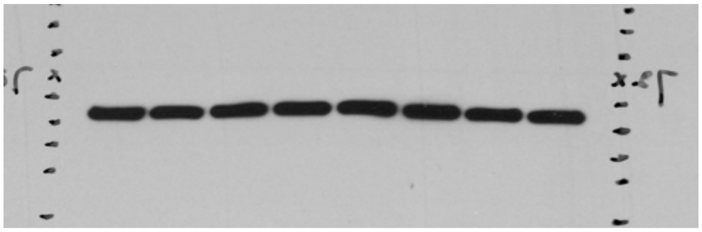

Actin

Supplementary Figure S8A

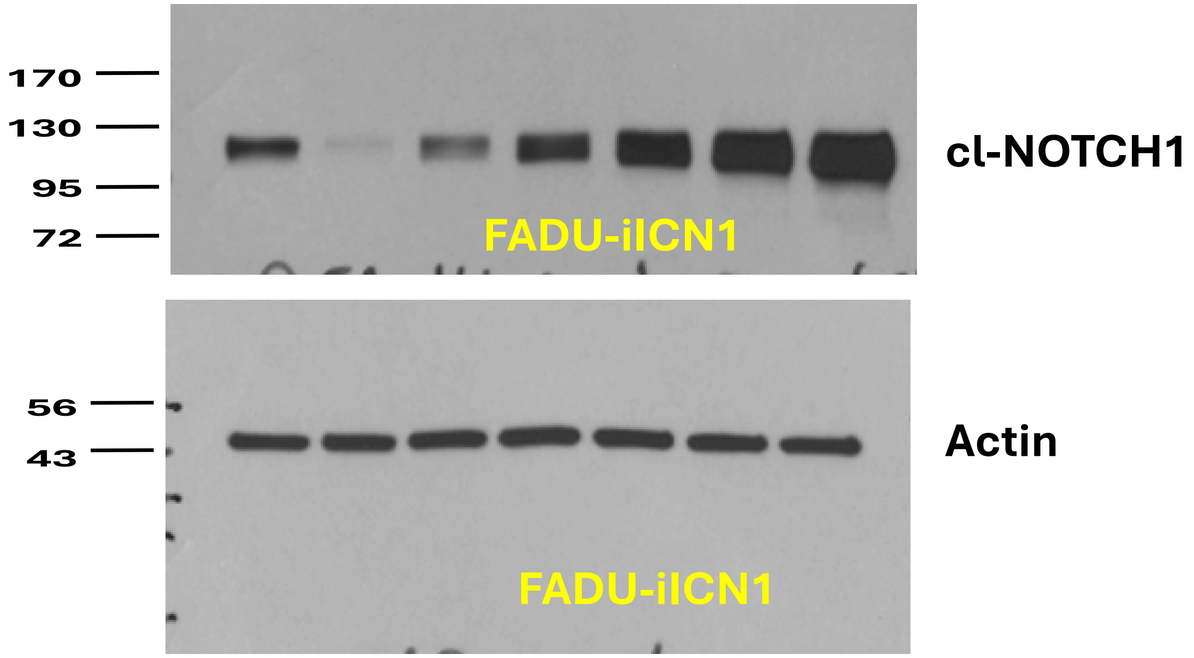

Figure S8C

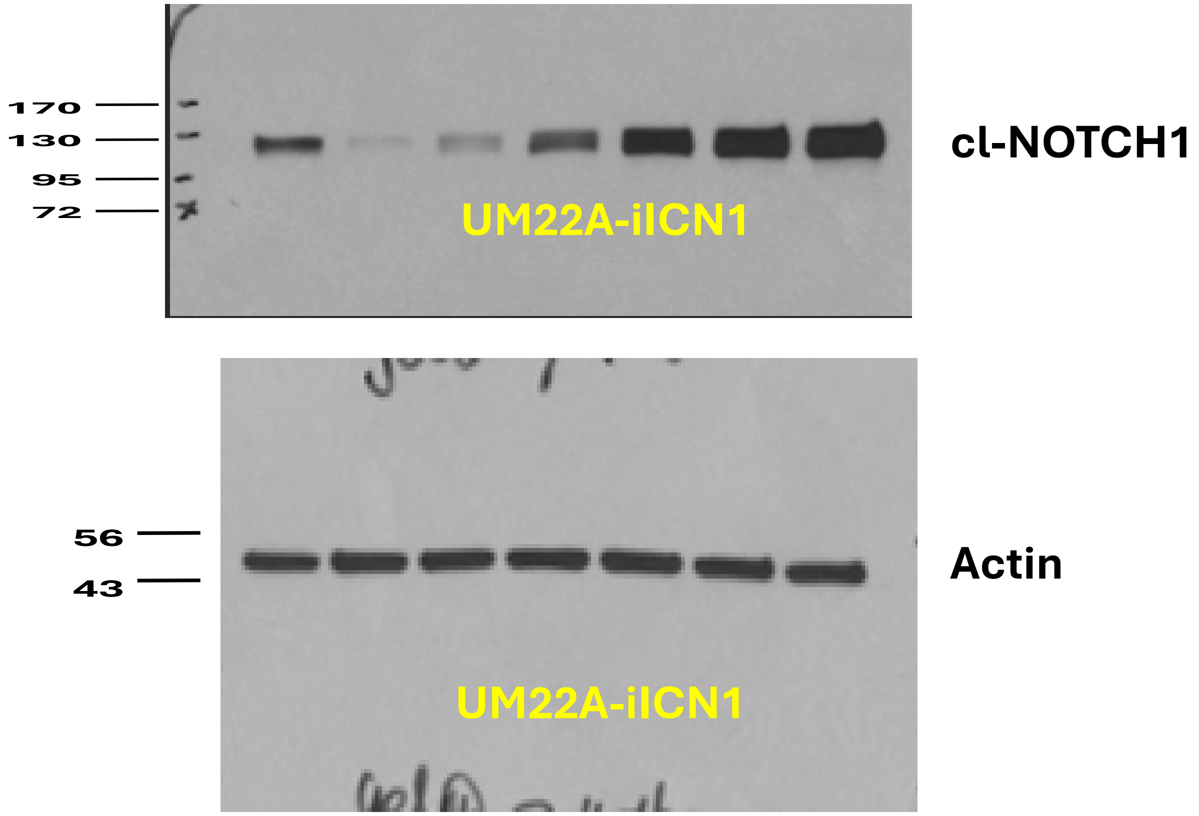

Supplementary Figure S12

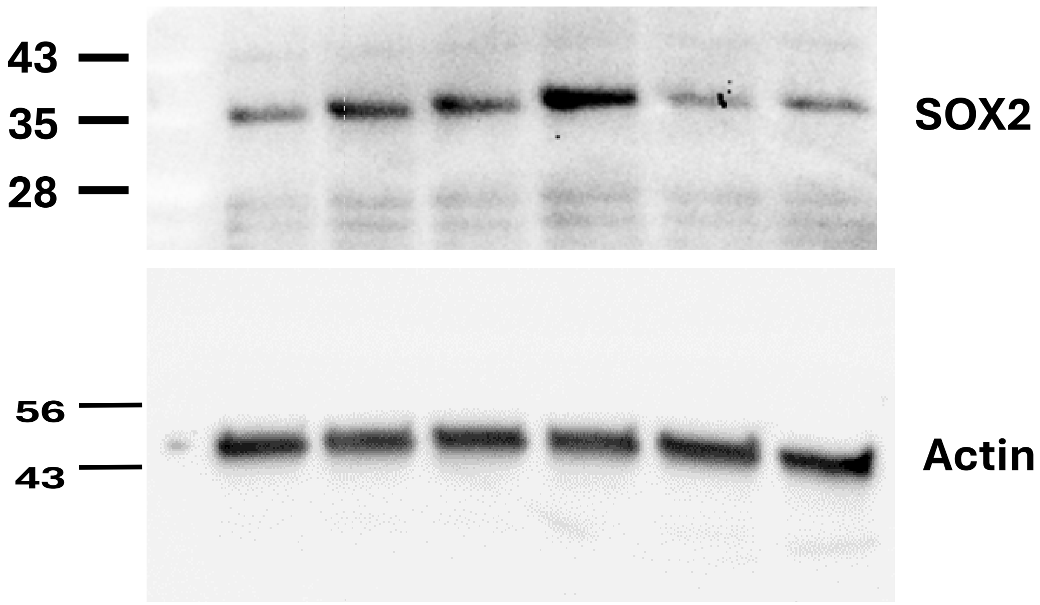

Supplementary Figure S15 A and B

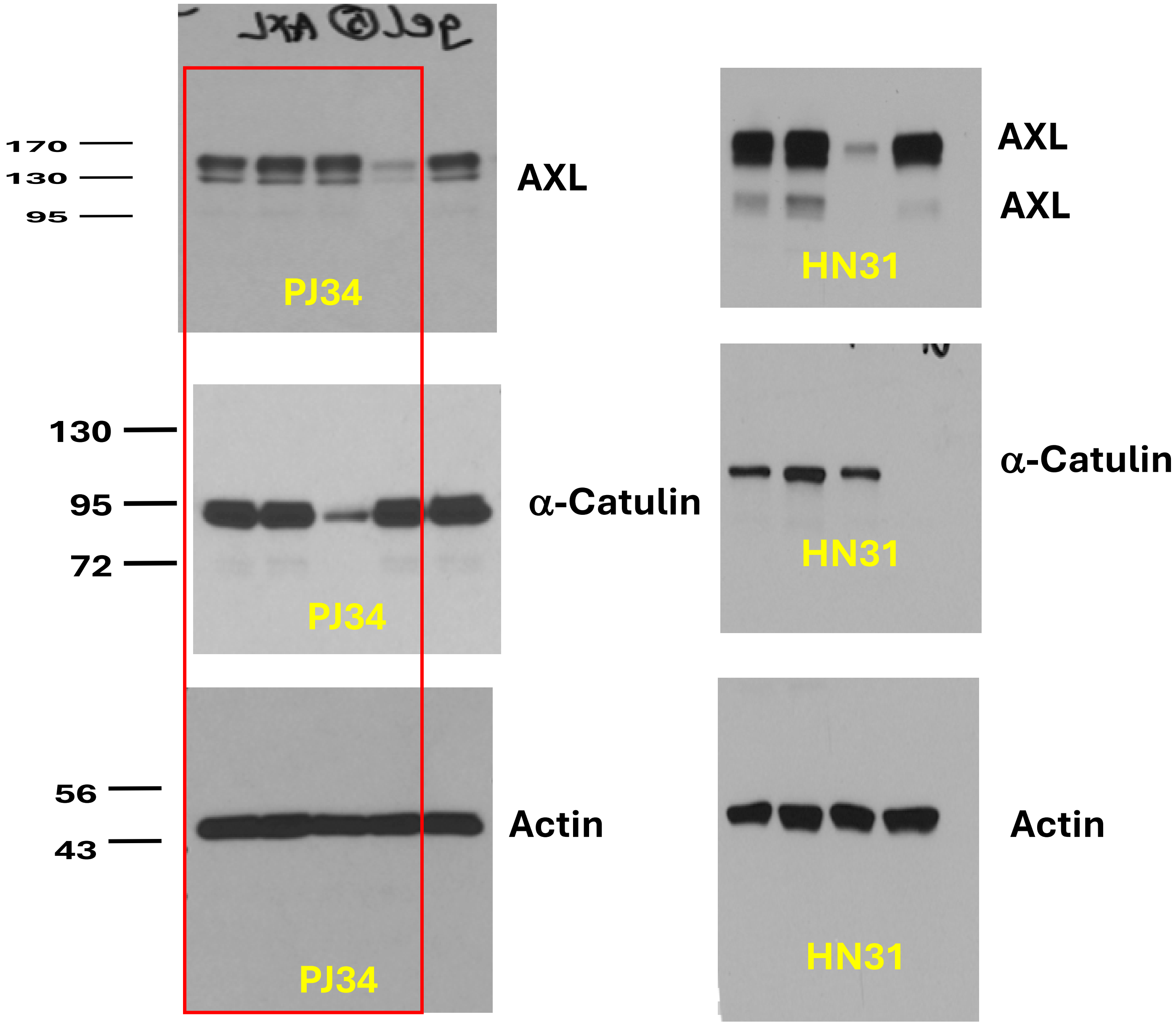

Supplementary Figure S15G

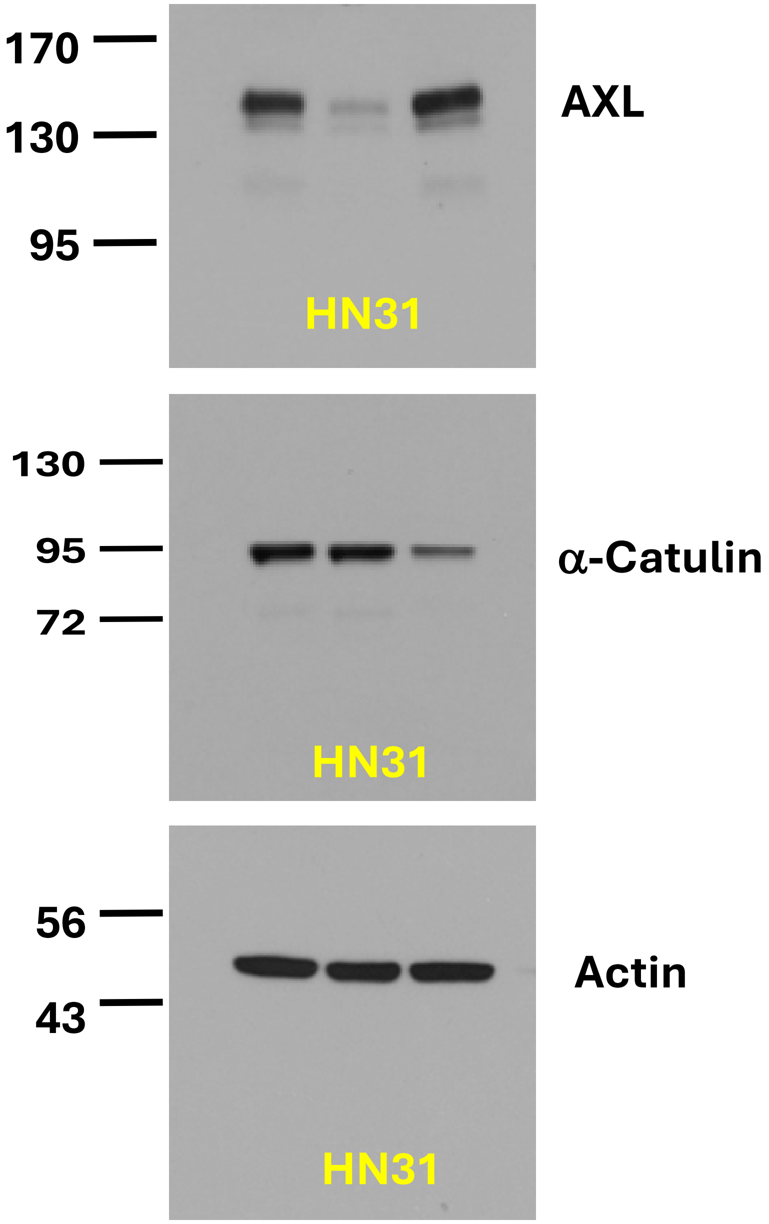

Supplement: Unedited blot and gel images [file jciinsight-11-202414-s186.pdf]
